# Prevalence, Deaths and Disability-Adjusted-Life-Years (DALYs) Due to Type 2 Diabetes and Its Attributable Risk Factors in 204 Countries and Territories, 1990-2019: Results From the Global Burden of Disease Study 2019

**DOI:** 10.3389/fendo.2022.838027

**Published:** 2022-02-25

**Authors:** Saeid Safiri, Nahid Karamzad, Jay S. Kaufman, Arielle Wilder Bell, Seyed Aria Nejadghaderi, Mark J. M. Sullman, Maziar Moradi-Lakeh, Gary Collins, Ali-Asghar Kolahi

**Affiliations:** ^1^ Social Determinants of Health Research Center, Department of Community Medicine, Faculty of Medicine, Tabriz University of Medical Sciences, Tabriz, Iran; ^2^ Research Center for Integrative Medicine in Aging, Aging Research Institute, Tabriz University of Medical Sciences, Tabriz, Iran; ^3^ Nutrition Research Center, Department of Biochemistry and Diet Therapy, School of Nutrition and Food Sciences, Tabriz University of Medical Sciences, Tabriz, Iran; ^4^ Department of Epidemiology, Biostatistics and Occupational Health, Faculty of Medicine, McGill University, Montreal, QC, Canada; ^5^ Health Sciences Integrated Program, Northwestern University, Chicago, IL, United States; ^6^ Department of Global Health and Social Medicine, Harvard University, Boston, MA, United States; ^7^ Systematic Review and Meta-analysis Expert Group (SRMEG), Universal Scientific Education and Research Network (USERN), Tehran, Iran; ^8^ Department of Life and Health Sciences, University of Nicosia, Nicosia, Cyprus; ^9^ Department of Social Sciences, University of Nicosia, Nicosia, Cyprus; ^10^ Preventive Medicine and Public Health Research Center, Iran University of Medical Sciences, Tehran, Iran; ^11^ Centre for Statistics in Medicine, NDORMS, Botnar Research Centre, University of Oxford, Oxford, United Kingdom; ^12^ NIHR Oxford Biomedical Research Centre, Oxford University Hospitals NHS Foundation Trust, Oxford, United Kingdom; ^13^ Social Determinants of Health Research Center, Shahid Beheshti University of Medical Sciences, Tehran, Iran

**Keywords:** type 2 diabetes mellitus, worldwide epidemiology, global epidemiology, prevalence, mortality, burden

## Abstract

**Aim:**

To report the point prevalence, deaths and disability-adjusted-life-years (DALYs) due to type 2 diabetes and its attributable risk factors in 204 countries and territories during the period 1990-2019.

**Methods:**

We used the data of the Global Burden of Disease (GBD) Study 2019 to report number and age-standardised rates per 100 000 population of type 2 diabetes. Estimates were reported with 95% uncertainty intervals (UIs).

**Results:**

In 2019, the global age-standardised point prevalence and death rates for type 2 diabetes were 5282.9 and 18.5 per 100 000, an increase of 49% and 10.8%, respectively, since 1990. Moreover, the global age-standardised DALY rate in 2019 was 801.5 per 100 000, an increase of 27.6% since 1990. In 2019, the global point prevalence of type 2 diabetes was slightly higher in males and increased with age up to the 75-79 age group, decreasing across the remaining age groups. American Samoa [19876.8] had the highest age-standardised point prevalence rates of type 2 diabetes in 2019. Generally, the burden of type 2 diabetes decreased with increasing SDI (Socio-demographic Index). Globally, high body mass index [51.9%], ambient particulate matter pollution [13.6%] and smoking [9.9%] had the three highest proportions of attributable DALYs.

**Conclusion:**

Low and middle-income countries have the highest burden and greater investment in type 2 diabetes prevention is needed. In addition, accurate data on type 2 diabetes needs to be collected by the health systems of all countries to allow better monitoring and evaluation of population-level interventions.

## Introduction

Diabetes mellitus (DM) describes a group of metabolic disorders which are characterized by high blood glucose levels. People with diabetes have an increased risk of developing a number of serious life-threatening health problems, which results in higher medical care costs, reduced quality of life and increased mortality (1). Diabetes is increasing globally (2, 3), and in 2019 the International Diabetes Federation estimated that worldwide there were 463 million people, aged 20 years and older with diabetes and this number is expected to increase to 700.2 million by 2045 (3). A considerable proportion of the burden of diabetes is caused by type 2 diabetes, but there is currently no research on the global prevalence and deaths due to type 2 diabetes alone, as the type-specific prevalence of diabetes is not reported by most research (3), which may be due to unreliability in the ascertainment of diabetes type and low data quality. To the best of our knowledge, the global number of prevalent cases of type 2 diabetes was first estimated in Global Burden of Disease (GBD) 2017, which found that about 463 million people live with type 2 diabetes (4). In addition, one study reported the incidence of type 2 diabetes using GBD 2017 data, but the prevalence and deaths due to the disease were not reported and the regional- and national-level patterns were not presented by development level (5). Moreover, previous research did not present inter-regional variations on the burden of type 2 diabetes that were attributable to different risk factors (5).

Type 2 diabetes imposes a considerable burden on the population’s health and its burden needs to be updated regularly using the most recently available data. Furthermore, many modifiable and non-modifiable risk factors have been found to be associated with type 2 diabetes (6) and the burden of these attributable risk factors need to be reported to help health policy makers with evidence-based decision making. In the last iteration of the GBD study, GBD 2019, the burden of type 2 diabetes and its attributable risk factors is estimated using the most up-to-date data available and this report supersedes the previous GBD studies (7). Thus, the present study aims to report the point prevalence, deaths and disability-adjusted life years (DALYs) due to type 2 diabetes and its attributable risk factors for 204 countries and territories by age, sex and Socio-demographic Index, from 1990 to 2019.

## Methods

### Overview

The GBD project is a comprehensive effort which assesses epidemiological levels and trends associated with diseases and injuries across the globe. In the last iteration of this study, GBD 2019, 369 diseases and injuries and 87 risk factors were estimated in 204 countries and territories, 7 super-regions and 21 regions from 1990 to 2019 (7). The general methodology for estimating the burden of diseases, injuries and risk factors for GBD 2019 has been reported in previous capstone papers (7, 8). The detailed information on fatal and non-fatal estimates can be found at https://vizhub.healthdata.org/gbd-compare/ and http://ghdx.healthdata.org/gbd-results-tool. The present study adhered to the Guidelines for Accurate and Transparent Health Estimates Reporting (GATHER) (**Table S1**) (9).

### Case Definition and Data Sources

The reference case definition for type 2 diabetes was fasting plasma glucose (FPG) ≥ 126 mg/dL (7 mmol/L), or being on drug or insulin treatment for type 2 diabetes. Alternative case definitions used in the data inputs were considered and adjusted prior to the modelling process. The list of alternative case definitions has been previously reported (7). The sequelae used for type 2 diabetes, included uncomplicated type 2 diabetes, diabetic neuropathy, diabetic foot due to neuropathy, diabetic neuropathy and amputation with treatment, diabetic neuropathy and amputation without treatment, moderate and severe vision impairment due to type 2 diabetes and blindness due to type 2 diabetes. The definition of each sequela is presented in **Table S2** and these correspond to E11-E11.1, E11.3-E11.9 codes in the International Classification of Diseases (ICD) version 10 (7).

A systematic review was conducted on the prevalence, incidence and deaths associated with diabetes using the relevant search terms for GBD 2019, which identified 717 records (7). An additional 281 records were identified through the reference lists of the above-mentioned articles, resulting in 998 records. A total of 600 records were excluded during the initial screening process, with the remaining 398 records being assessed for inclusion. Finally, 36 and 12 studies were included in the analysis for combined diabetes (type 2 and type 1 diabetes) and type 1 diabetes, respectively. In addition, the Global Health Data Exchange was searched for multi-country surveys, national surveys and longitudinal studies that measured diabetes or fasting plasma glucose. In order to obtain any remaining sources of information, leaders in the field were contacted to ensure that the database was as comprehensive as possible (7). Diabetes estimates by type were only available in 20% of the articles and the diagnostic criteria were not sufficiently detailed in those separately reporting type 2 diabetes. Thus, the estimates of type 2 diabetes were calculated by subtracting the estimates of type 1 diabetes from the combined estimates of diabetes for each age, sex, and location, from 1990 to 2019 (7).

To incorporate all available data related to population-representative estimates of diabetes, measures of blood sugar (glycated hemoglobin A1c, oral glucose tolerance test, post prandial glucose test) were also included as definitions of diabetes. Mean fasting plasma glucose was also used in populations where data on diabetes was not available. Four types of sources were used to estimate the burden of diabetes: a) estimates of diabetes in a representative population; b) estimates of mean fasting plasma glucose in a representative population; c) individual-level data of fasting plasma glucose measured from surveys, and d) claim and insurance data from the US and Taiwan (7). When a study reported both mean fasting plasma glucose and the prevalence of diabetes, the latter was used. Individual-level data was collapsed and aggregated to produce estimates for each age group, sex, location, and year. We note that 171 counties had data on diabetes and that 1,289 data sources were used in the estimation process (7). A systematic review was also conducted using relevant search terms for outcomes of diabetes, including amputation due to diabetes, diabetic neuropathy and foot ulcers, with relevant papers being extracted and included in the modelling processes (7). More detailed information on the data inputs used in the GBD study can be found at http://ghdx.healthdata.org/gbd-2019/data-input-sources.

### Data Processing and Disease Model

The overall prevalence of diabetes was estimated using DisMod MR‐2.1, a Bayesian metaregression tool. Using data on the prevalence and incidence for diabetes, DisMod-MR produced estimates for the prevalence of diabetes for each age, sex, geographic location, and year. In addition, the “age-standardised prevalence rates of people with obesity” and “year” were used as country-level covariates in DisMod MR‐2.1 (7).

The outcomes of diabetes were also modelled through DisMod MR‐2.1 and their estimates were provided for each age, sex, geographic location, and year. All proportion draws from the neuropathy/foot/amputation models were multiplied by the parent diabetes model so that all estimates were in the same population‐space. This was to ensure that the sum of the prevalence of neuropathy, moderate vision loss, severe vision loss and blindness due to diabetes did not exceed 90% of the prevalence of all diabetes. If the sum exceeded 90%, then the individual outcomes were re-scaled to 90% (7). The vision loss sequala was not directly modelled, as it has been previously estimated as impairment in the GBD study. The calculation process takes these estimates into account when estimating uncomplicated diabetes, amputation due to diabetes, diabetic neuropathy, and diabetic foot. The same checks were also conducted for the prevalence of amputation due to diabetes and the prevalence of foot ulcers due to diabetes, which were not allowed to exceed 90% of the prevalence of neuropathy due to diabetes. This treats foot ulcers and amputation as mutually exclusive categories, by assuming that a patient cannot have both simultaneously. Uncomplicated diabetes was estimated to be the remainder of the diabetes cases, excluding neuropathy and vision loss (7). Finally, the prevalence of type 2 diabetes was calculated by subtracting the prevalence of type 1 diabetes from the combined estimates of diabetes for each age, sex, and location from 1990 to 2019 (7).

Type-specific diabetes mortality was estimated using deaths from vital registration sources using ICD-10 codes only. Diabetes type-specific information was not available in ICD-9 codes, or from deaths determined by verbal autopsy. The Cause of Death Ensemble model (CODEm) was used for estimating deaths due to diabetes (7). An age range of 15-95+ years old was set for type 2 diabetes and the following data manipulations were undertaken as part of the modelling process. The ICD-10 diabetes data were reported as type 1, type 2, or unspecified. A regression model was developed to estimate the fraction of unspecified diabetes mellitus that were type 1 and type 2. The data from 703 country-years was used to inform the regression. This was because these country-years had more than 50% of the deaths classified as type 1 or type 2 AND at least 70% of type-specific deaths in people >25 years were coded as type 2. Since there were separate regressions to estimate the proportion of type 1 and type 2 diabetes mellitus, the predicted proportions were re-scaled to one. Where ICD-10 data was reported, these proportions were then applied to the number of deaths coded as unspecified diabetes in each location, year, and sex. Using this approach, the number of deaths due to type 2 diabetes was estimated.

### Compilation of Results

The disability weights (DWs) for all sequelae were determined using the GBD 2013 European Disability Weights Measurement Study (10). The severity levels, lay descriptions, and associated DWs for each outcome related to diabetes are presented in **Table S2**. The prevalence of each severity category was multiplied by severity-specific DWs to calculate years lived with disability (YLDs).

The years of life lost (YLLs) were then calculated by multiplying the number of deaths in an age group by the remaining life expectancy in that age group, taken from the GBD standard life table. DALYs were then calculated as the sum of YLLs and YLDs. Uncertainty was propagated by sampling 1000 draws at each computational step, combining uncertainty from multiple sources, such as input data, corrections of measurement error and estimates of residual non-sampling error. Uncertainty intervals (UIs) were defined as the 25^th^ and 975^th^ values of the ordered draws. All estimates were presented as counts and rates per 100,000 population. The rates were age-standardised using the GBD standard population (7). Smoothing Splines models were used to examine the association between the burden of type 2 diabetes burden, in terms of DALYs, and the SDI for 21 regions and 204 countries and territories (11). SDI is a composite indicator of lag-dependent income per capita, and is comprised of the gross domestic product per capita that has been smoothed over the preceding 10 years, average years of schooling for the population older than 15 years of age, and total fertility rate under the age of 25. It ranges from 0 (less developed) to 1 (most developed). The mapping of the age-standardised point prevalence, deaths and DALY rates were conducted using R software, version 3.5.2.

### Risk Factors

The risk factors with evidence of causation for type 2 diabetes were included in the present study (7). The percentage of DALYs, due to type 2 diabetes, that were attributable to high body mass index (12), smoking (13), secondhand smoke (13), low physical activity (14), ambient particulate matter pollution (15), household air pollution (16), high temperature (17), low temperature (17), diet high in processed meat (18), diet high in red meat (18), diet high in sugar-sweetened beverages, diet low in fiber (19), diet low in fruit (20), diet low in nuts and seeds (21) and diet low in whole grains (22) were reported. Definitions for these risk factors can be found elsewhere (**Table S3**) (7).

## Results

### Global Level

Globally, there were 437.9 million prevalent cases of type 2 diabetes in 2019, with an age-standardised point prevalence of 5282.9 per 100,000 populations, which represents a 49% increase since 1990. Type 2 diabetes accounted for 1472.9 thousand deaths in 2019, with an age-standardised rate of 18.5 per 100,000 population, which has increased 10.8% since 1990 (**Table 1**). In 2019, the number of DALYs due to type 2 diabetes worldwide was 66.3 million, with an age-standardised rate of 801.5 DALYs per 100,000 population, an increase of 27.6% since 1990 (**Table 1**).

**Table 1:** Prevalent cases, deaths and DALYs for type 2 diabetes in 2019 and percentage change of age- standardised rates (ASRs) per 100,000, by GBD region, from 1990 to 2019 (Generated from data available from http://ghdx.healthdata.org/gbd-results-tool).

	Prevalence (95% UI)			Deaths (95% UI)			DALYs (95% UI)		
	No (95% UI) (in million)	ASRs per 100,000 (95% UI)	Percentage change in ASRs between 1990 and 2019	No (95% UI) (in thousand)	ASRs per 100,000 (95% UI)	Percentage change in ASRs between 1990 and 2019	No (95% UI) (in million)	ASRs per 100,000 (95% UI)	Percentage change in ASRs between 1990 and 2019
**Global**	**437.9 (402**, 477)	**5282.9 (4853.6, 5752.1)**	**49 (47.1, 50.6)**	**1472.9 (1371.9, 1565.9)**	**18.5 (17.2, 19.7)**	**10.8 (4.4, 17.4)**	**66.3 (55.5, 79)**	**801.5 (670.6, 954.4)**	**27.6 (22, 33)**
**High-income Asia Pacific**	**13.2 (12, 14.5)**	**3744.9 (3383.3, 4140.2)**	**26.1 (21.8, 30.2)**	**21.5 (18.5, 23.6)**	**4.2 (3.7, 4.6)**	**-45.8 (-51.6, -42)**	**1.4 (1.1, 1.9)**	**383.2 (285.3, 495.5)**	**0 (-8.9, 8)**
**High-income North America**	**38.2 (35.4, 41.2)**	**6725.2 (6220.5, 7224.4)**	**54.6 (48.8, 61.4)**	**80.3 (73.8, 84.1)**	**12.3 (11.4, 12.9)**	**-7.2 (-10.3, -4.2)**	**4.4 (3.5, 5.4)**	**748.2 (596, 931.1)**	**26.9 (19.4, 33.6)**
**Western Europe**	**40.8 (37, 44.7)**	**5360.8 (4812.9, 5915.5)**	**60.5 (54.3, 67.3)**	**93.4 (82.5, 99.8)**	**8.5 (7.6, 9.1)**	**-34.6 (-37.8, -32)**	**4.2 (3.3, 5.4)**	**515.8 (389.5, 663.9)**	**12.5 (1.8, 21.3)**
**Australasia**	**1.5 (1.4, 1.7)**	**3368.8 (3006.6, 3764.6)**	**72.8 (59.2, 87.4)**	**4.8 (4.2, 5.2)**	**8.7 (7.8, 9.5)**	**-19.8 (-25.3, -14.2)**	**0.2 (0.2, 0.3)**	**419.6 (320.7, 531.7)**	**15.9 (4.6, 27.8)**
**Andean Latin America**	**2.4 (2.2, 2.7)**	**4250.7 (3898.4, 4602.7)**	**68.4 (61.9, 75.2)**	**13.1 (10.9, 15.4)**	**24 (20.1, 28.3)**	**24 (1.8, 48.1)**	**0.5 (0.4, 0.6)**	**873.5 (722.3, 1045.3)**	**33.6 (17.1, 49.3)**
**Tropical Latin America**	**12.3 (11.2, 13.4)**	**4979.1 (4525.8, 5443.8)**	**14.4 (11.2, 18.1)**	**65.3 (59.2, 68.9)**	**28 (25.3, 29.6)**	**-12 (-16.5, -7.9)**	**2.5 (2.1, 2.9)**	**1020.4 (868, 1194.5)**	**-5.7 (-10.1, -1.9)**
**Central Latin America**	**20.8 (19.2, 22.4)**	**8505.6 (7837.2, 9133.4)**	**30.7 (27, 34.3)**	**102.7 (90.5, 115.6)**	**44.6 (39.3, 50.2)**	**2.7 (-9.1, 15)**	**4.2 (3.6, 5)**	**1746.5 (1485.3, 2073.9)**	**10.8 (2.9, 18.8)**
**Southern Latin America**	**4.1 (3.7, 4.4)**	**5031 (4528.4, 5494.2)**	**79.8 (68.4, 91.3)**	**14.8 (13.7, 15.7)**	**17.4 (16.1, 18.5)**	**-11.2 (-16.2, -5.9)**	**0.6 (0.5, 0.7)**	**722.4 (588.2, 878.5)**	**19.1 (9.6, 27.8)**
**Caribbean**	**3.8 (3.6, 4.2)**	**7479.2 (6913.6, 8083.3)**	**42.4 (37.2, 48.3)**	**18.4 (15.5, 21.8)**	**35.5 (29.9, 42.1)**	**-13.3 (-26.1, 1.8)**	**0.8 (0.6, 0.9)**	**1483.1 (1226, 1800.5)**	**5.3 (-6, 16.5)**
**Central Europe**	**10.8 (9.8, 11.7)**	**5619.9 (5099.5, 6110.2)**	**53.4 (49.7, 57.3)**	**26.8 (23.3, 30.6)**	**11.9 (10.4, 13.6)**	**1.7 (-10.7, 14.7)**	**1.5 (1.1, 1.9)**	**730.2 (559, 923.1)**	**25.8 (17, 32.8)**
**Eastern Europe**	**9.1 (8.3, 10.1)**	**2856.6 (2582.5, 3157)**	**31.8 (28.1, 35.6)**	**21.6 (19, 24.1)**	**6.1 (5.4, 6.8)**	**48.3 (32.3, 65.5)**	**1.3 (1, 1.6)**	**376 (295.1, 468.2)**	**33.6 (28.1, 39.2)**
**Central Asia**	**4.4 (4.1, 4.8)**	**5343 (4931.1, 5771.4)**	**94 (86.4, 102.6)**	**16.6 (14.9, 18.5)**	**23.6 (21.4, 26.1)**	**163.8 (138.1, 192.6)**	**0.8 (0.7, 1)**	**1013.8 (841.9, 1219.1)**	**120.2 (106.9, 137.6)**
**North Africa and Middle East**	**32.9 (29.9, 36.2)**	**6753.3 (6170.2, 7394.2)**	**85.5 (80.8, 90.3)**	**95.4 (84.9, 107.4)**	**25.2 (22.4, 28.2)**	**1.7 (-10.4, 14.9)**	**4.8 (3.9, 5.9)**	**1060.8 (872.1, 1279.1)**	**31.2 (18.3, 42.2)**
**South Asia**	**98.1 (88.4, 108.8)**	**6375.2 (5752.2, 7067.8)**	**68.2 (64.2, 72.1)**	**337.9 (301, 379.8)**	**28.1 (25, 31.6)**	**27.5 (8.2, 48.9)**	**15.1 (12.4, 18)**	**1049.7 (869.3, 1244.9)**	**43.4 (30, 57.1)**
**Southeast Asia**	**31.2 (28.7, 34)**	**4875.1 (4493.5, 5285.6)**	**59.7 (54.9, 64.7)**	**214.8 (193, 236.3)**	**38.1 (34.1, 41.7)**	**17.3 (3.3, 32.6)**	**8.1 (7, 9.3)**	**1273.4 (1103.9, 1452.4)**	**27 (14.9, 40)**
**East Asia**	**93.3 (85.4, 101.9)**	**4502.3 (4110.8, 4930)**	**23.2 (16.3, 29.8)**	**182.9 (157.8, 208.4)**	**9.6 (8.3, 11)**	**5.4 (-12.2, 23.7)**	**10.2 (8.1, 12.5)**	**487.6 (387.8, 602.7)**	**9.1 (0.3, 17.5)**
**Oceania**	**1 (0.9, 1.1)**	**11086.1 (10196.7, 12087.4)**	**67.5 (61.7, 73.5)**	**7.8 (6.3, 9.6)**	**121 (100.2, 146.5)**	**38 (8.3, 68.6)**	**0.3 (0.2, 0.4)**	**3703.4 (3060, 4399.3)**	**42.8 (17.7, 70.2)**
**Western Sub-Saharan Africa**	**7 (6.3, 7.7)**	**3279.9 (2962.2, 3612.2)**	**52.1 (49, 55.5)**	**55.1 (47.2, 63.3)**	**35.6 (30.8, 40.2)**	**20.8 (3.4, 40.1)**	**1.9 (1.6, 2.2)**	**994.8 (851, 1148.4)**	**25.2 (9.9, 41)**
**Eastern Sub-Saharan Africa**	**6.1 (5.4, 6.8)**	**3052.3 (2760.6, 3377.6)**	**29.2 (26.5, 32.2)**	**49.1 (43.6, 55.7)**	**36.5 (32.5, 40.9)**	**-11.4 (-25.6, 1.2)**	**1.7 (1.5, 2)**	**1026.8 (889.8, 1182.4)**	**-8.5 (-22, 2.6)**
**Central Sub-Saharan Africa**	**3.3 (2.9, 3.7)**	**4955.9 (4481.2, 5480.5)**	**42.1 (37.2, 47.4)**	**16.9 (13.9, 20.7)**	**38.3 (32.1, 46.2)**	**-10.9 (-27.6, 8.9)**	**0.7 (0.6, 0.9)**	**1265.1 (1049.1, 1533.2)**	**1.3 (-14.3, 18.3)**
**Southern Sub-Saharan Africa**	**3.4 (3.1, 3.7)**	**5605.7 (5133.9, 6083)**	**61 (55.1, 68.1)**	**33.9 (31.4, 36.6)**	**68.5 (63.2, 73.8)**	**60.7 (44.1, 77.7)**	**1.1 (0.9, 1.2)**	**1878 (1679.9, 2098.8)**	**53.1 (41.6, 65.1)**

### Regional Level

In 2019, the age-standardised point prevalence of type 2 diabetes per 100,000 population were highest in Oceania [11086.1], Central Latin America [8505.6] and the Caribbean [7479.2]. Eastern Europe [2856.6], Eastern Sub-Saharan Africa [3052.3] and Western Sub-Saharan Africa [3279.9] observed the lowest age-standardised rates (**Table 1**). Oceania [121.0], Southern Sub-Saharan Africa [68.5)] and Central Latin America [44.6] had the highest age-standardised death rates from type 2 diabetes per 100,000 population. These rates were lowest for High-income Asia Pacific [4.2], Eastern Europe [6.1] and Western Europe [8.5] (**Table 1**). Oceania [3703.4], Southern Sub-Saharan Africa [1878], and Central Latin America [1746.5] also had the highest age-standardised DALY rates from type 2 diabetes per 100,000 population in 2019. In contrast, Eastern Europe [376], High-income Asia Pacific [383.2] and Australasia [419.6] had the lowest age-standardised DALY rates (**Table 1**). The age-standardised point prevalence, deaths and DALY rates of type 2 diabetes per 100,000 population in 2019, for all GBD regions, are presented in **Figures S1**–**3**, respectively.

Almost all regions revealed increases in the age-standardised point prevalence, deaths and DALY rates of type 2 diabetes over the last thirty years. Central Asia [94.0%], North Africa and Middle East [85.5%] and Southern Latin America [79.8%] experienced the largest increases over the duration of this study (**Table 1**). In the same period, the largest increases in the age-standardised death rates of type 2 diabetes were found in Central Asia [163.8%], Southern Sub-Saharan Africa [60.7%] and Eastern Europe [48.3%] (**Table 1**). In contrast, High-income Asia-Pacific [-45.8%], Western Europe [-34.6%)] and Australasia [-19.8%] were the regions with the largest decreases. In addition, Central Asia [120.2%], Southern Sub-Saharan Africa [53.1%)] and South Asia [43.4%] had the largest increases in the age-standardised DALY rates of type 2 diabetes from 1990 to 2019. Tropical Latin America [-5.7%] was the only region in which a decrease in the age-standardised DALY rate was found (**Table 1**). The percentage change, from 1990 to 2019, in the age-standardised point prevalence, death and DALY rates for type 2 diabetes are presented in **Figures S4**–**6**, respectively.

The number of prevalent cases of type 2 diabetes increased from 148.5 million in 1990 to 437.9 million in 2019, with South Asia, East Asia and Western Europe having the largest number of prevalent cases in 2019 (**Figure S7** and **Table S4**). Likewise, the number of deaths due to type 2 diabetes increased remarkably from 606.4 thousand in 1990 to 1472.9 thousand in 2019, with South Asia, Southeast Asia and East Asia having the highest numbers of deaths due to type 2 diabetes in 2019 (**Figure S8** and **Table S5**).

### National Level

**Figure 1:**
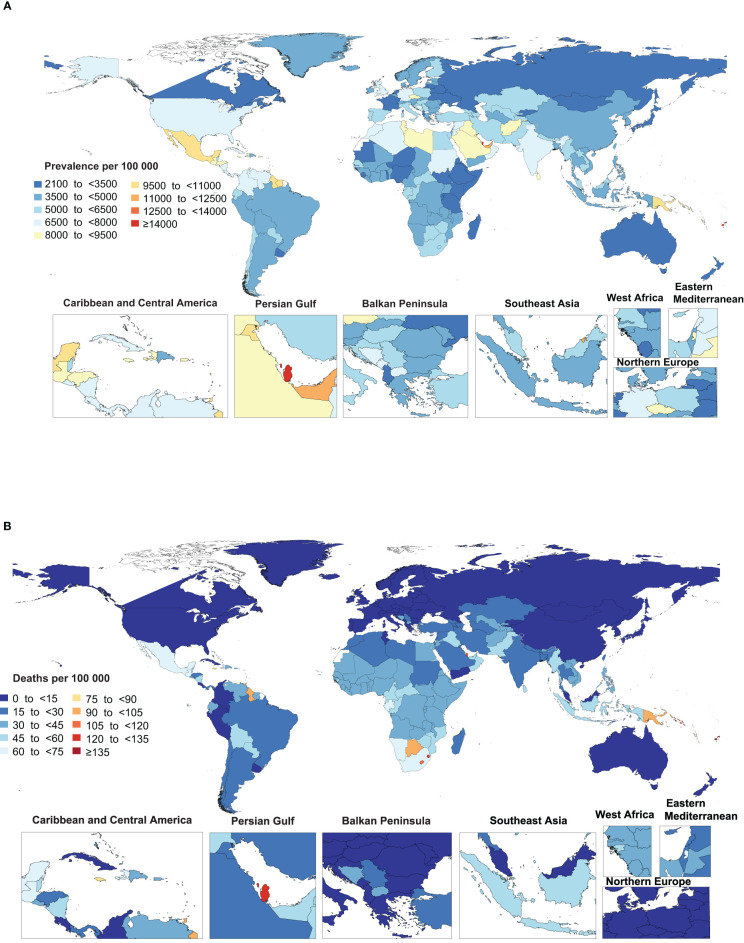
Age-standardized point prevalence **(A)** and death **(B)** rate of type 2 diabetes per 100,000 population in 2019, by country. (Generated from data available from http://ghdx.healthdata.org/gbd-results-tool).

In 2019, the national age-standardised point prevalence of type 2 diabetes ranged from 2174.5 to 19876.8 cases per 100,000 population. American Samoa [19876.8], the Marshall Islands [19523.9] and Niue [16454.2] had the three highest age-standardised point prevalences of type 2 diabetes per 100,000 population in 2019. In contrast, Mongolia [2174.5], Sierra Leone [2420.3] and Ethiopia [2476.5] had the lowest rates (**Figure 1A** and **Table S4**).

The national age-standardised death rates of type 2 diabetes in 2019 varied from 2 to 257.4 cases per 100,000 population. The highest rates were observed in Fiji [257.4], Kiribati [204.0] and the Federated States of Micronesia [169.1], with lowest rates being found in Japan [2.0], Belarus [2.2] and Singapore [2.4] (**Figure 1B** and **Table S5**).

The national age-standardised DALY rate of type 2 diabetes in 2019 ranged from 278.2 to 6884.3 cases per 100,000 population. The highest rates were observed in Fiji [6884.3], Kiribati [6161.4] and the Federated States of Micronesia [4896.3]. Conversely, the lowest rates were seen in France [278.2], Belarus [278.7] and Japan [313.8] (**Figure S9** and **Table S6**).

**Figure 2:**
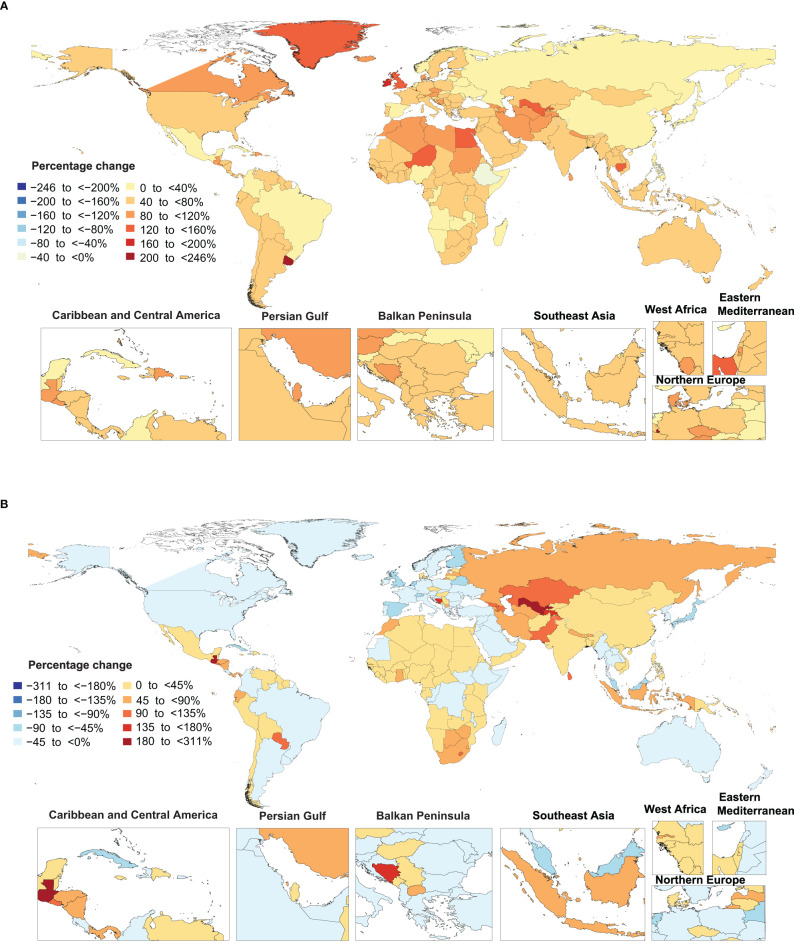
Percentage change in age-standardized point prevalence **(A)** and deaths **(B)** rate of type 2 diabetes per 100,000 population from 1990 to 2019, by country. (Generated from data available from http://ghdx.healthdata.org/gbd-results-tool).

The percentage change in age-standardised point prevalence from 1990 to 2019 differed substantially between countries, with Luxembourg [245.7%], Uruguay [228.8%] and Ireland [179.7%] showing the largest increases during the measurement period. In contrast, Ethiopia [-7.6%] was the only country that showed a decrease in the age-standardised point prevalence during the study duration (**Figure 2A** and **Table S4**).

Uzbekistan [310.4%], Cabo Verde [270.2%] and Guatemala [262.9%] showed the largest increases in the age-standardised death rates of type 2 diabetes during the measurement period. In contrast, Singapore [-86.9%], Japan [-67.6%] and Cyprus [-58%] showed the largest decreases in the age-standardised death rates of type 2 diabetes over the last 30 years (**Figure 2B** and **Table S5**).

Uzbekistan [219.8%], Guatemala [185.9%] and Cabo Verde [150.1%] showed the largest increases in the age-standardised DALY rates of type 2 diabetes during the measurement period. In contrast, Ethiopia [-41%], Singapore [-40.5%] and Cyprus [-38.0%] showed the largest decreases in the age-standardised DALY rate of type 2 diabetes during the same period (**Figure S10** and0 **Table S6**).

### Age and Sex Patterns

**Figure 3:**
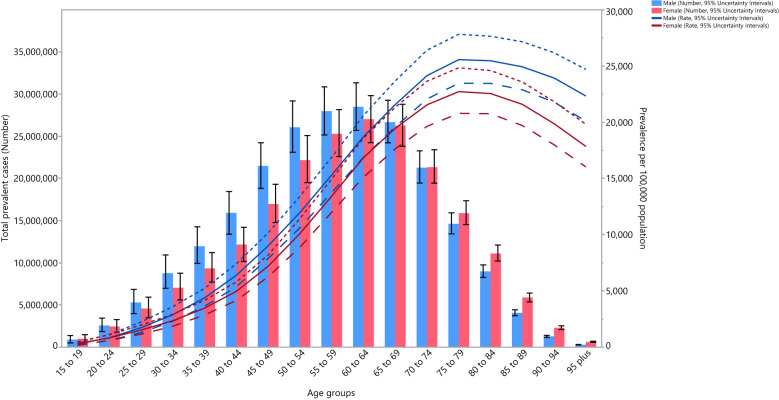
Global number of prevalent cases and prevalence of type 2 diabetes per 100,000 population by age and sex in 2019; Dotted and dashed lines indicate 95% upper and lower uncertainty intervals, respectively. (Generated from data available from http://ghdx.healthdata.org/gbd-results-tool).

In 2019, the global point prevalence of type 2 diabetes was slightly higher in males and increased with age up to the 75-79 years age group, and then decreased with increasing age. Similarly, the number of prevalent cases increased with age and peaked in those aged 60-64 years, for both males and females, and then decreased with age (**Figure 3**).

In 2019, the global death rate of type 2 diabetes was slightly higher in males, peaking in those aged 95^+^ years. The number of deaths reached its highest in the 70-74 and 80-84-year age groups for males and females, respectively, after which there was a decline with increasing age (**Figure S11**).

The global DALY rate of type 2 diabetes was slightly higher in males and increased with age up to the 85-89 and 80-84 age groups for males and females, respectively, and then decreased with increasing age. Similarly, the number of DALYs increased with age, peaking in the 60-64 and 65-69 age groups for males and females, respectively, and then decreased as age increased (**Figure S12**).

Moreover, in 2019, the global number and rate of YLDs due to type 2 diabetes were higher than YLLs up to the 65-69 years age group, and then the number and YLL rates were much higher up to the oldest age group (**Figure S13**).

### Association With the Socio-Demographic Index (SDI)

At the regional level there was no clear association between SDI and the age-standardised DALY rate of type 2 diabetes, suggesting that the burden of type 2 diabetes does not vary according to socio-economic development. However, in general the burden of type 2 diabetes decreased with increasing SDI. Oceania, Southern Sub-Saharan Africa, Central Latin America, Caribbean and High-income North America had higher than expected DALY rates, based upon their socio-demographic development (as measured by the SDI), from 1990 to 2019. In contrast, Eastern Europe, East Asia, High-income Asia-Pacific and Australasia had lower than expected burdens within this period (**Figure S14**).

**Figure 4:**
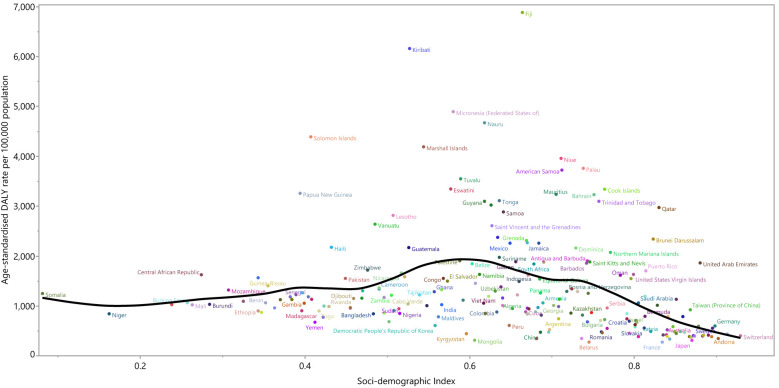
Age-standardized DALY rates of type 2 diabetes for 204 countries and territories by SDI, in 2019; Expected values based on the Socio-demographic Index and disease rates in all locations are shown as the black line. Each point shows the observed age-standardized DALY rate for each country in 2019. DALY=disability adjusted life years. SDI= Socio-demographic Index (Generated from data available from http://ghdx.healthdata.org/gbd-results-tool).

At a country-level, in 2019, the burden of type 2 diabetes increased slightly with increasing socio-economic development up to an SDI of around 0.6 and then decreased as SDI increased (**Figure 4**). Countries and territories, such as Fiji, Kiribati, the Federated States of Micronesia, Nauru, and the Solomon Islands had much higher than expected burdens, whereas countries and territories such as Belarus, China. Mongolia, Kirgizstan, and Peru had much lower than expected burdens (**Figure 4**).

### Risk Factors

**Figure 5:**
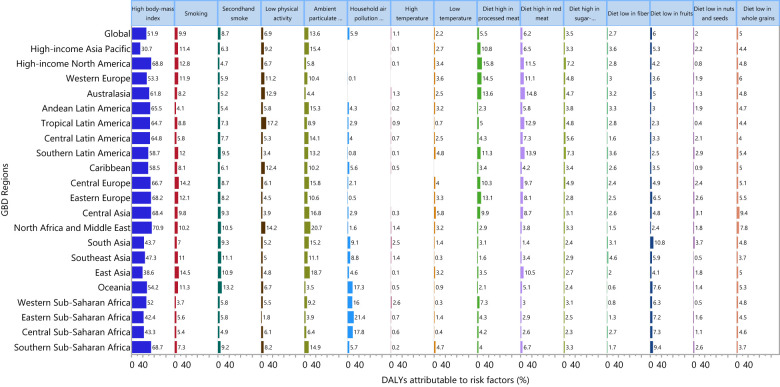
Percentage of DALYs due to type 2 diabetes attributable to risk factors for 21 GBD regions in 2019. DALY, disability adjusted life years (Generated from data available from http://ghdx.healthdata.org/gbd-results-tool).

Although the proportion of DALYs due to type 2 diabetes attributable to the individual risk factors differed across the GBD regions, globally high body mass index [51.9%], ambient particulate matter pollution [13.6%] and smoking [9.9%] had the three highest proportions of attributable DALYs (**Figure 5**). For males, high body mass index [49.3%] smoking [15.8%] and ambient particulate matter [14.2%] had the highest proportions of attributable DALYs due to type 2 diabetes. Similarly, the three main contributors for females were: high body mass index [54.6%], ambient particulate matter pollution [13.1%] and second-hand smoke [10.7%] (**Figures S15**, **S16**).

The proportion of DALYs due to type 2 diabetes attributable to the individual risk factors also differed by age group, with particularly large differences being observed for high body mass index and smoking. The highest proportions of attributable DALYs were in the 35-39 age group for high body mass index and the 45-49 age group for smoking (**Figure S17**). For males, the proportion of DALYs due to type 2 diabetes attributable to high body mass index and smoking were highest in the 35-39 and 55-59 age groups, respectively (**Figure S18**). For females, the proportion of DALYs was highest for high body mass index in the 35-39 age group, but there was no notable variation for smoking (**Figure S19**).

## Discussion

In 2019, there were 437.9 million prevalent cases, 1.5 million deaths and 66.3 million DALYs due to type 2 diabetes. Substantial increases were observed in the age-standardised prevalence, death and DALY rates of 49%, 10.8% and 27.6%, respectively. The findings of the present study and the International Diabetes Federation estimates could not be directly compared, as the present study reported the burden of type 2 diabetes, while the International Diabetes Federation presented type 1 and type 2 diabetes combined. However, both studies documented large increases in the burden associated with diabetes (3).

Although the increase in type 2 diabetes was observed at the global level, regions with middle development levels, such as Oceania, Central Latin America and Caribbean had the highest burden from type 2 diabetes. In 2019, middle and low income countries and territories, such as Fiji, Kiribati, the Federated States of Micronesia and Nauru, had the highest burden of type 2 diabetes. The genetics of these populations have not changed, but the increase is happening within the context of individuals that are genetically predisposed to have T2D. Furthermore, changes in lifestyle and behavior are likely to be driven by changes in the wider environment, such as the Westernization in some countries or changes to the built up environment, transport infrastructure, food manufacturing, etc. High body mass index, or having a BMI > 25, is the most important risk factor for type 2 diabetes, as 51.9% of DALYs due to type 2 diabetes were attributable to this risk factor. Previous research has reported that the prevalence of those with a BMI > 25 is increasing for all ages at the global level (23, 24) and further increases are expected in the absence of effective control strategies. Decreasing the proportion of the population with a BMI > 25 would be particularly beneficial to control type 2 diabetes in regions such as North Africa and Middle East, High-income North America and Southern Sub-Saharan Africa, as based on our finding the burden of type 2 diabetes attributable to high body mass index was highest for these regions.

Ambient particulate matter has previously been found to be associated with an elevated risk of type 2 diabetes (25), and several mechanisms have been proposed. Evidence shows that particulate matter exposure is associated with elevated systemic inflammation and oxidative stress, endoplasmic reticulum stress, impaired endothelial function, cardiac autonomic nervous system dysfunction, and mitochondrial dysfunction (15). The present study found that about 13.6% of the burden was attributable to this risk factor. A study reported that the global prevalence of exposure to ambient particulate matter was about 26% in 2019, which has increased substantially from 15.7% in 1990 and thus control measures are needed to reduce exposure (7). It is well known that the status of particulate matter pollution is highly significant in some Asian countries. As such, various tactics and techniques have been used in those areas to reduce or suppress pollution in recent years. In China, the Ministry of Environmental Protection (MEP) reported that the mean concentration of PM2.5 in 74 cities was 76 μg m−3, which is far higher than the standard guidelines (26). In addition, ‘The Airborne Pollution Prevention and Control Action Plan (2013–17)’ has been established by the government of China to reduce PM pollution by installing emissions-cutting exhaust filters, reducing coal use, tightening vehicle emission standards, etc. (27) Similar prevention programs are needed in countries with the highest burden, in order to reduce the burden of disease attributable to ambient particulate matter.

Smoking was the third largest risk factor for type 2 diabetes globally, with 9.9% of the burden of type 2 diabetes being attributable to this risk factor. It has been suggested that smoking impacts body weight and composition, peripheral insulin sensitivity, and pancreatic β cell function, which may be the potential mechanisms behind the relationship between smoking and diabetes (28). Although the daily smoking prevalence is decreasing across the world, and it has been reported to have decreased by 28.4% and 34.4% for men and women, respectively, from 1990 to 2015 (29), its attributable burden remains substantial and needs to be further decreased. The present study shows that smoking prevention programs would particularly beneficial in regions such as East Asia, Central Europe and High-income North America Sub-Saharan Africa, as the attributable burden of smoking on type 2 diabetes was found to be 14.5%, 14.2% and 12.8%, respectively.

In addition, 6.9% of the type 2 diabetes burden was found to be attributable to physical inactivity. A recent study estimated that the global age-standardised prevalence of physical inactivity was 3.54% and that this figure was increasing in most of the countries and so we can expect this burden to increase in the future (7).

Poor diets, including those high in red and processed meat and those low in fruit and whole grains, are another risk factor that makes a substantial contribution to the burden of type 2 diabetes. Improvements in diets are needed to reduce their contribution to the burden of type 2 diabetes as much as possible. Population-level dietary interventions, include such things as mass media campaigns, food and menu labeling, food pricing strategies (subsidies and taxation), and worksite wellness programmes are needed. However, the effectiveness of these interventions have yet to be evaluated for several types of poor diet, including diets low in whole grains, along with those high in red and processed meat (30).

Although the management of life-style factors may be associated with decreased incidence of type 2 diabetes, timely diagnosis, health care utilization, and quality of care for patients with type 2 diabetes may also reduce the burden of diabetes in a community or country. A study on patients with type 2 diabetes from 49 countries, outside of North America and Western Europe, found that the proportion of patients with HbA1c<7% decreased from 36% to 30.1% between 2005 and 2017 (31, 32). Another multicenter study, from outside the USA and Europe, found that only 20 to 30% of patients with type 2 diabetes had the recommended HbA1c level (<7%) (33).

On average, only 20 to 50% of patients were treated with organ-protective drugs (notably statins and renin– angiotensin system inhibitors), or underwent periodic eye and foot examinations and blood or urine testing, in accordance with international recommendations (33).

In contrast, some high income countries have made substantial improvements in health care services, complication rates and deaths. For example, a study in the United States found that acute myocardial infarction events, death from hyperglycaemic crisis, stroke incidence and lower extremity amputation have decreased by 67.8%, 64.4%, 52.7% and 51.4%, respectively, during the period 1990-2010 (34). In addition, there were substantial variations between and within low- and high-income countries, in terms of the awareness, diagnosis, and treatments to control diabetes (35). However, the increasing incidence of cardiovascular disease and death rates in low-income countries (e.g., India), compared with the decreasing rate of cardiovascular disease in North America, might reflect differences in resources, capacity, access to healthcare, and healthcare organisation (32). The United States spent about 53% of the global healthcare expenditure on diabetes, while India spent less than 1% of the world’s total expenditure, despite having one of the largest populations of patients with diabetes. In total, all 18 countries included in the African region, as defined by the International Diabetes Federation, spent only 0.3% of the global expenditure on diabetes (36). This means that diabetes prevention and management programs need to be appropriately funded, especially in low-income countries, in order to reduce the burden and complications associated with type 2 diabetes. In addition, strong leadership and political support, underpinned by high quality data, are needed to continuously monitor and evaluate prevention programs. Hence, our findings on the global burden of type 2 diabetes may be useful for informing policy and decision making for the appropriate management of type 2 diabetes.

### Strengths and Limitations of the Study

The present study provides the most up-to-date information on the burden of type 2 diabetes and its attributable risk factors. In addition, several of the complications associated with type 2 diabetes were also considered in estimating the burden of this disease and these were expressed as DALYs, while previous studies have only reported the prevalence associated with both types of diabetes combined (3).

The current study had several limitations. Firstly, there was no data for some countries and so the burden of type 2 diabetes for those countries was estimated using the GBD modelling process. This study highlights the need for better data in health systems to allow the monitoring of diseases and risk factors and evaluation of the effectiveness of population-based interventions. Secondly, the attributable risk factors of type 2 diabetes in this report were assumed to be independent and joint distributions were not considered, which could have inflated our estimated PAFs. Thirdly, subnational estimates have not been provided for several countries, although several studies have indicated large variations within these countries for diseases and risk factors. Fourthly, “*chronic kidney disease due to type 2 diabetes”* was not reported here, as this has been separately estimated and reported in the GBD study (7). Finally, the comorbidities of type 2 diabetes could not be taken into account when estimating the burden of diabetes.

### Conclusions and Policy Implications

There is large inter-country variation in the burden of type 2 diabetes and this burden has increased substantially since 1990. Low and middle income countries have the highest burden and more investment in type 2 diabetes prevention is needed. In addition, accurate data on type 2 diabetes needs to be collected by the health systems of all countries to allow better monitoring and evaluation of population-level interventions.

## Data Availability Statement

The data used for these analyses are all publicly available at https://vizhub.healthdata.org/gbd-compare/ and http://ghdx.healthdata.org/gbd-results-tool.

## Ethics Statement

The present report was reviewed and approved by the Ethics Committee of Shahid Beheshti University of Medical Sciences, Tehran, Iran (IR.SBMU.RETECH.REC.1400.076).

## Author Contributions

SS and A-AK designed the study. SS analyzed the data and performed the statistical analyses. SS, NK, JK, AWB, SN, MS, GC, and A-AK drafted the initial manuscript. All authors reviewed the drafted manuscript for critical content. All authors approved the final version of the manuscript.

## Funding

The Bill and Melinda Gates Foundation, who were not involved in any way in the preparation of this manuscript, funded the GBD study. The Shahid Beheshti University of Medical Sciences, Tehran, Iran (Grant No. 25675-4-2) also supported the present report.

## Author Disclaimer

This study is based on publicly available data and solely reflects the opinion of its authors and not that of the Institute for Health Metrics and Evaluation.

## Conflict of Interest

The authors declare that the research was conducted in the absence of any commercial or financial relationships that could be construed as a potential conflict of interest.

## Publisher’s Note

All claims expressed in this article are solely those of the authors and do not necessarily represent those of their affiliated organizations, or those of the publisher, the editors and the reviewers. Any product that may be evaluated in this article, or claim that may be made by its manufacturer, is not guaranteed or endorsed by the publisher.

## Supplementary Material

Supplementary Table 1 Guidelines for accurate and transparent health estimates reporting (GATHER) checklist.

Supplementary Table 2 Sequelae for type 2 diabetes and corresponding disability weights in the GBD 2019 Study.

Supplementary Table 3 Definition of risk factors attributable to type 2 diabetes in the GBD 2019 Study.

Supplementary Table 4 Prevalent cases of type 2 diabetes in 1990 and 2019 for both sexes and percentage change in the age-standardised rates (ASRs) per 100,000 by location (Generated from data available from http://ghdx.healthdata.org/gbd-results-tool).

Supplementary Table 5 Deaths due to type 2 diabetes in 1990 and 2019 for both sexes and percentage change in the age-standardised rates (ASRs) per 100,000 by location (Generated from data available from http://ghdx.healthdata.org/gbd-results-tool).

Supplementary Table 6 DALYs due to type 2 diabetes in 1990 and 2019 for both sexes and percentage change in the age-standardised rates (ASRs) per 100,000 by location (Generated from data available from http://ghdx.healthdata.org/gbd-results-tool).

Supplementary Figure 1 The age-standardized prevalence of type 2 diabetes in 2019 for 21 Global Burden of Disease regions by sex. (Generated from data available from http://ghdx.healthdata.org/gbd-results-tool).

Supplementary Figure 2 The age-standardized death rates of type 2 diabetes in 2019 for the 21 Global Burden of Disease regions by sex. (Generated from data available from http://ghdx.healthdata.org/gbd-results-tool).

Supplementary Figure 3 The age-standardized DALY rates of type 2 diabetes in 2019 for the 21 Global Burden of Disease regions by sex. DALY=disability adjusted life years. (Generated from data available from http://ghdx.healthdata.org/gbd-results-tool).

Supplementary Figure 4 The percentage change in age-standardized prevalence of type 2 diabetes from 1990 to 2019 for the 21 Global Burden of Disease regions by sex. (Generated from data available from http://ghdx.healthdata.org/gbd-results-tool).

Supplementary Figure 5 The percentage change in age-standardized death rates of type 2 diabetes from 1990 to 2019 for the 21 Global Burden of Disease regions by sex. (Generated from data available from http://ghdx.healthdata.org/gbd-results-tool).

Supplementary Figure 6 The percentage change in age-standardized DALY rates of type 2 diabetes from 1990 to 2019 for the 21 Global Burden of Disease regions by sex. DALY=disability adjusted life years. (Generated from data available from http://ghdx.healthdata.org/gbd-results-tool).

Supplementary Figure 7 Number of prevalent cases of type 2 diabetes from 1990 to 2019 for the 21 Global Burden of Disease regions. (Generated from data available from http://ghdx.healthdata.org/gbd-results-tool).

Supplementary Figure 8 Number of deaths due to type 2 diabetes from 1990 to 2019 for 21 Global Burden of Disease regions. (Generated from data available from http://ghdx.healthdata.org/gbd-results-tool).

Supplementary Figure 9 Age-standardized DALY rate of type 2 diabetes per 100,000 population in 2019, by country. DALY=disability adjusted life years. (Generated from data available from http://ghdx.healthdata.org/gbd-results-tool).

Supplementary Figure 10 Percentage change in age-standardized DALY rate of type 2 diabetes per 100,000 population from 1990 to 2019, by country. DALY=disability adjusted life years. (Generated from data available from http://ghdx.healthdata.org/gbd-results-tool).

Supplementary Figure 11 Global number of deaths and death rate of type 2 diabetes per 100,000 population, by age and sex in 2019; Dotted and dashed lines indicate 95% upper and lower uncertainty intervals, respectively. (Generated from data available from http://ghdx.healthdata.org/gbd-results-tool).

Supplementary Figure 12 Global number of DALYs and DALY rate of type 2 diabetes per 100,000 population by age and sex, 2019; Dotted and dashed lines indicate 95% upper and lower uncertainty intervals, respectively. DALY=disability adjusted life years. (Generated from data available from http://ghdx.healthdata.org/gbd-results-tool).

Supplementary Figure 13 Global number and rate of YLDs and YLLs due to type 2 diabetes per 100,000 population by age, 2019; Dotted and dashed lines indicate 95% upper and lower uncertainty intervals, respectively. DALY=disability adjusted life years, YLD= years lived with disability, YLLs= years of life lost. (Generated from data available from http://ghdx.healthdata.org/gbd-results-tool).

Supplementary Figure 14 Age-standardized DALY rates of type 2 diabetes for the 21 Global Burden of Disease regions by Socio-demographic Index, 1990–2019; Expected values based on Socio-demographic Index and disease rates in all locations are shown as the black line. Twenty-eight points are plotted for each GBD region and show the observed age-standardized DALY rates from 1990 to 2019 for that region. DALY=disability adjusted life years. (Generated from data available from http://ghdx.healthdata.org/gbd-results-tool).

Supplementary Figure 15 Percentage of DALYs due to type 2 diabetes attributable to risk factors for the 21 GBD regions, males, in 2019. DALY=disability adjusted life years (Generated from data available from http://ghdx.healthdata.org/gbd-results-tool).

Supplementary Figure 16 Percentage of DALYs due to type 2 diabetes attributable to risk factors for the 21 GBD regions, females, in 2019. DALY=disability adjusted life years (Generated from data available from http://ghdx.healthdata.org/gbd-results-tool).

Supplementary Figure 17 Percentage of DALYs due to type 2 diabetes attributable to each risk factor, by age, in 2019. DALY=disability adjusted life years (Generated from data available from http://ghdx.healthdata.org/gbd-results-tool).

Supplementary Figure 18 Percentage of DALYs due to type 2 diabetes attributable to each risk factor by age, for males, in 2019. DALY=disability adjusted life years (Generated from data available from http://ghdx.healthdata.org/gbd-results-tool).

Supplementary Figure 19 Percentage of DALYs due to type 2 diabetes attributable to each risk factor by age, for females, in 2019. DALY=disability adjusted life years (Generated from data available from http://ghdx.healthdata.org/gbd-results-tool).

## Abbreviations

DM, Diabetes mellitus; GBD, Global Burden of Disease; DALY, disability-adjusted life year; GATHER, Guidelines for Accurate and Transparent Health Estimates Reporting; FPG, fasting plasma glucose; ICD, International Classification of Diseases; CODEm, Cause of Death Ensemble model; DW, disability weight; YLD, years lived with disability; YLL, years of life lost; UI, Uncertainty intervals; SDI, Socio-demographic Index; BMI, Body mass index; MEP, Ministry of Environmental Protection; PAF, Population-attributable fraction.
