# Supplementary material for: Prevalence, Deaths and Disability-Adjusted-Life-Years (DALYs) Due to Type 2 Diabetes and Its Attributable Risk Factors in 204 Countries and Territories, 1990-2019: Results From the Global Burden of Disease Study 2019
Source: Front Endocrinol (Lausanne). 2022 Feb 25;13:838027. doi: 10.3389/fendo.2022.838027 (PMC8915203; doi:10.3389/fendo.2022.838027)
Supplement: Supplementary Table 1 — Guidelines for accurate and transparent health estimates reporting (GATHER) checklist. [file DataSheet_1.zip › Supplementary Figures/Supplementary Figure S9.PDF]

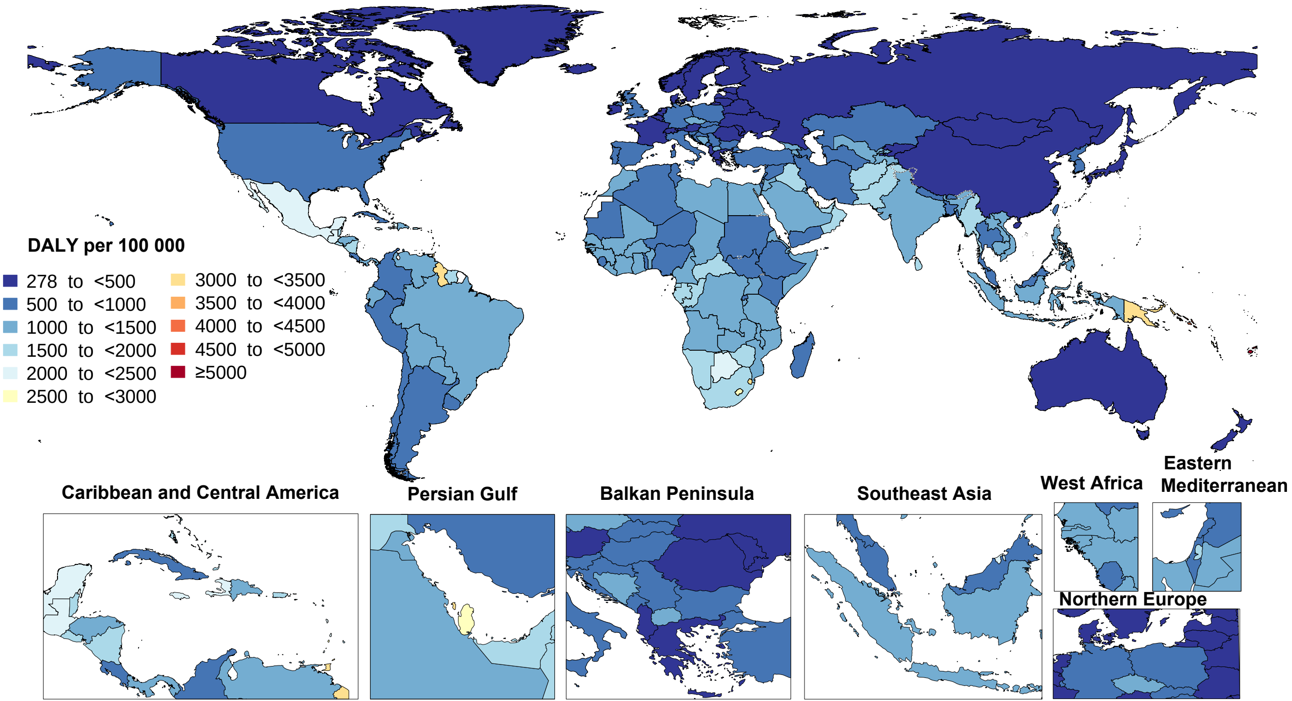
DALY per 100 000
278 to <500
500 to <1000
1000 to <1500
1500 to <2000
2000 to <2500
2500 to <3000
3000 to <3500
3500 to <4000
4000 to <4500
4500 to <5000
≥5000
Caribbean and Central America
Persian Gulf
Balkan Peninsula
Southeast Asia
West Africa
Eastern Mediterranean
Northern Europe
