# Supplementary material for: Prevalence, Deaths and Disability-Adjusted-Life-Years (DALYs) Due to Type 2 Diabetes and Its Attributable Risk Factors in 204 Countries and Territories, 1990-2019: Results From the Global Burden of Disease Study 2019
Source: Front Endocrinol (Lausanne). 2022 Feb 25;13:838027. doi: 10.3389/fendo.2022.838027 (PMC8915203; doi:10.3389/fendo.2022.838027)
Supplement: Supplementary Table 1 — Guidelines for accurate and transparent health estimates reporting (GATHER) checklist. [file DataSheet_1.zip › Supplementary Tables/Supplementary Table S2.DOC]

| **Table S2: Sequelae for type 2 diabetes and corresponding disability weights in the GBD 2019 Study** | | |
| --- | --- | --- |
| **Severity level** | **Lay description** | **DW**  **(95% CI)** |
| Uncomplicated type 2 Diabetes | Has a chronic disease that requires medication every day and causes some worry, but minimal interference with daily activities. | 0.049  (0.031 – 0.072) |
| Diabetic neuropathy | Has pain, tingling, and numbness in the arms, legs,  hands, and feet. The person sometimes gets cramps and  muscle weakness. | 0.133  (0.089 – 0.187) |
| Diabetic neuropathy with  diabetic foot | Has a sore on the foot that is swollen and causes some difficulty in walking. | a |
| Diabetic neuropathy with  treated amputation | Has lost part of one leg, leaving pain and tingling in the stump.  The person has an artificial leg that helps in moving around. | a |
| Diabetic neuropathy with  untreated amputation | Has lost part of one leg, leaving pain and tingling in the stump.  The person does not have an artificial leg, has frequent sores,  and uses crutches. | a |
| Moderate vision loss due to type 2 diabetes | Has vision problems that make it difficult to recognize faces or  objects across a room. | 0.031  (0.019 – 0.049) |
| Severe vision loss due to type 2 diabetes | Has severe vision loss, which causes difficulty in daily  activities, some emotional impact (for example worry), and  some difficulty going outside the home without assistance. | 0.184  (0.125 – 0.259) |
| Blindness due to type 2 diabetes | Is completely blind, which causes great difficulty in some  daily activities, worry and anxiety, and great difficulty going outside the home without assistance. | 0.187  (0.124 – 0.26) |
| ^a^ The DWs are produced from a combination of two health states: neuropathy and diabetic foot/amputation.  **GBD: Global Burden of Disease**  **DW: Disability weight** | | |
