# Supplementary material for: Prevalence, Deaths and Disability-Adjusted-Life-Years (DALYs) Due to Type 2 Diabetes and Its Attributable Risk Factors in 204 Countries and Territories, 1990-2019: Results From the Global Burden of Disease Study 2019
Source: Front Endocrinol (Lausanne). 2022 Feb 25;13:838027. doi: 10.3389/fendo.2022.838027 (PMC8915203; doi:10.3389/fendo.2022.838027)
Supplement: Supplementary Table 1 — Guidelines for accurate and transparent health estimates reporting (GATHER) checklist. [file DataSheet_1.zip › Supplementary Tables/Supplementary Table S3.DOC]

| **Table S3:** Definition of risk factors attributable to type 2 diabetes in the GBD 2019 Study | | |
| --- | --- | --- |
| **Risk factor** | **Case definition** | **Reference** |
| High body-mass  index | High body-mass index (BMI) for adults (ages 20+) is defined as BMI greater than 20 to 25 kg/m2. High BMI for children (ages 1–19) is defined as being overweight or obese based on International Obesity Task Force standards. | ^1^ |
| Smoking | As in GBD 2017, the prevalence of current smoking and the prevalence of former smoking were estimated using data from nationally representative cross-sectional household surveys. Current smokers were defined as individuals who currently use any smoked tobacco product on a daily or occasional basis. Former smokers were defined as individuals who quit using all smoked tobacco products for at least six months, where possible, or according to the definition used by the survey. | ^1^ |
| Secondhand smoke | Secondhand smoke exposure was defined as current exposure to secondhand tobacco smoke at home, at work, or in other public places. Household composition was used as a proxy for non-occupational secondhand smoke exposure and made the assumption that all persons living with a daily smoker are exposed to tobacco smoke. Surveys were used to estimate the proportion of individuals exposed to secondhand smoke at work. Only non-smokers were considered to be exposed to secondhand smoke. Non-smokers were defined as all persons who are not daily smokers. Ex-smokers and occasional smokers were considered to be non-smokers in this analysis. Exposure was evaluated for both children and adults. | ^1^ |
| Low physical activity | Physical activity performed by adults older than 25 years of age was measured, for a duration of at least ten minutes at a time, across all domains of life (leisure/recreation, work/household and transport). Frequency, duration and intensity of activity was used to calculate total metabolic equivalent-minutes per week. MET (Metabolic Equivalent) is the ratio of the working metabolic rate to the resting metabolic rate. One MET is equivalent to 1 kcal/kg/hour and is equal to the energy cost of sitting quietly. A MET is also defined as the oxygen uptake in ml/kg/min with one MET equal to the oxygen cost of sitting quietly, around 3.5 ml/kg/min. | ^1^ |
| Ambient particulate matter pollution | Exposure to ambient particulate matter pollution is defined as the population-weighted annual average mass concentration of particles with an aerodynamic diameter less than 2.5 micrometers (PM2.5) in a cubic meter of air. This measurement is reported in μg/m3. | ^1^ |
| Household air pollution | Exposure to household air pollution from solid fuels (HAP) is estimated from both the proportion of individuals using solid cooking fuels and the level of PM2.5 air pollution exposure for these individuals. Solid fuels in this analysis included coal, wood, charcoal, dung, and agricultural residues. | ^1^ |
| Non-optimal temperature | The exposure to non-optimal temperatures is defined as the same day exposure to ambient temperatures that are either warmer or colder than the temperature associated with the minimum mortality risk. Specifically, the theoretical minimum risk exposure level (TMREL) was defined for temperature as the temperature that is associated with the lowest overall mortality attributable to the risk, in a given location and year. Given varying exposure-response curves for the different mean annual temperature zones, as well as spatially and temporally varying cause compositions, TMRELs was estimated by year and location and did not use a globally uniform TMREL. High temperature (heat) exposure was defined as exposure to temperatures warmer than this TMREL and low temperature (cold) was defined as temperatures colder than this TMREL. | ^1^ |
| Diet high in processed  meat | Any intake (in grams per day) of meat preserved by smoking, curing,  salting, or the addition of chemical preservatives. | ^1^ |
| Diet high in red meat | Any intake (in grams per day) of red meat including beef, pork, lamb, and goat, but excluding poultry, fish, eggs, and all processed meats. | ^1^ |
| Diet high in sugar sweetened  Beverages (SSBs) | Any intake (in grams per day) of beverages with ≥50 kcal per 226.8 gram serving, including carbonated beverages, sodas, energy drinks, fruit drinks, but excluding 100% fruit and vegetable juices. | ^1^ |
| Diet low in fibre | Average daily consumption (in grams per day) of less than 21-22 grams of fibre from all sources, including fruits, vegetables, grains, legumes, and pulses. | ^1^ |
| Diet low in fruit | Average daily consumption (in grams per day) of less than 310-340 grams of fruit, including fresh, frozen, cooked, canned, or dried fruit, excluding fruit juices and salted or pickled fruits. | ^1^ |
| Diet low in nuts and seeds | Average daily consumption (in grams per day) of less than 10-19 grams of nuts and seeds, including tree nuts, seeds and peanuts. | ^1^ |
| Diet low in whole grains | Average daily consumption (in grams per day) of less than 140-160 grams of whole grains (bran, germ, and endosperm in their natural proportion) from breakfast cereals, bread, rice, pasta, biscuits, muffins, tortillas, pancakes, and other sources | ^1^ |

**Reference (s):**
