# Supplementary material for: Prevalence, Deaths and Disability-Adjusted-Life-Years (DALYs) Due to Type 2 Diabetes and Its Attributable Risk Factors in 204 Countries and Territories, 1990-2019: Results From the Global Burden of Disease Study 2019
Source: Front Endocrinol (Lausanne). 2022 Feb 25;13:838027. doi: 10.3389/fendo.2022.838027 (PMC8915203; doi:10.3389/fendo.2022.838027)
Supplement: Supplementary Table 1 — Guidelines for accurate and transparent health estimates reporting (GATHER) checklist. [file DataSheet_1.zip › Supplementary Tables/Supplementary Table S4.DOC]

| **Table S4: Prevalent cases of type 2 diabetes in 1990 and 2019 and the percentage change of age-standardised rates (ASRs) per 100,000 by location**  **(Generated from data available from http://ghdx.healthdata.org/gbd-results-tool)** | | | | | |
| --- | --- | --- | --- | --- | --- |
|  | **1990** | | **2019** | | **Percentage change in ASRs per 100,000** |
|  | **No (95% UI)** | **ASRs per 100,000 (95% UI)** | **No (95% UI)** | **ASRs per 100,000 (95% UI)** |  |
| **Global** | **148454494 (135461385 , 162602314)** | **3546.5 (3243.7 , 3862.7)** | **437906572 (402043333 , 477018182)** | **5282.9 (4853.6 , 5752.1)** | **49 (47.1 , 50.6)** |
| **High-income North America** | **14376106 (13089254 , 15599096)** | **4349 (3956.3 , 4725.3)** | **38227777 (35420171 , 41150184)** | **6725.2 (6220.5 , 7224.4)** | **54.6 (48.8 , 61.4)** |
| **Canada** | **491523 (442127 , 537109)** | **1513.3 (1356.5 , 1659.2)** | **2025746 (1768729 , 2281508)** | **3202.3 (2783.5 , 3629.1)** | **111.6 (88.4 , 133.6)** |
| **Greenland** | **528 (452 , 613)** | **1427.4 (1251.9 , 1628.3)** | **2647 (2352 , 2968)** | **3676.3 (3256.9 , 4112.1)** | **157.5 (137.5 , 181.6)** |
| **United States of America** | **13883727 (12644726 , 15092804)** | **4657.6 (4239.7 , 5067.1)** | **36198778 (33619916 , 38939480)** | **7145.6 (6622.1 , 7671.7)** | **53.4 (47.5 , 60.5)** |
| **Australasia** | **446945 (403896 , 492639)** | **1950 (1761.3 , 2150.9)** | **1520272 (1362209 , 1688561)** | **3368.8 (3006.6 , 3764.6)** | **72.8 (59.2 , 87.4)** |
| **Australia** | **379532 (342466 , 419475)** | **1985.1 (1786.7 , 2195.3)** | **1307261 (1165523 , 1461180)** | **3429.9 (3053.3 , 3853.7)** | **72.8 (56.6 , 90.3)** |
| **New Zealand** | **67413 (59188 , 76295)** | **1779.2 (1563.8 , 2015.4)** | **213011 (192544 , 233940)** | **3053.5 (2723 , 3387.7)** | **71.6 (59.2 , 86.3)** |
| **High-income Asia Pacific** | **6092466 (5530302 , 6653348)** | **2970.7 (2698.7 , 3245)** | **13222172 (12034544 , 14509722)** | **3744.9 (3383.3 , 4140.2)** | **26.1 (21.8 , 30.2)** |
| **Brunei Darussalam** | **12439 (11546 , 13549)** | **10415.1 (9782.7 , 11172.6)** | **45732 (42086 , 49876)** | **12355.3 (11470.5 , 13343.8)** | **18.6 (13 , 25.3)** |
| **Japan** | **4831871 (4342190 , 5329455)** | **2855.2 (2567.9 , 3150.3)** | **8761940 (7858624 , 9712894)** | **3359 (3000.8 , 3742.5)** | **17.6 (13 , 22.7)** |
| **Singapore** | **131779 (120708 , 142628)** | **5259.7 (4839.1 , 5657)** | **436918 (393395 , 480057)** | **5435.7 (4887.5 , 5973.3)** | **3.3 (-4.5 , 12.5)** |
| **Republic of Korea** | **1116377 (1023256 , 1213719)** | **3339 (3069.5 , 3624.5)** | **3977581 (3634911 , 4347289)** | **4592.6 (4191.1 , 5017.9)** | **37.5 (27.7 , 47.1)** |
| **Western Europe** | **18172423 (16552307 , 19755867)** | **3339.3 (3028.1 , 3637.9)** | **40769763 (37016368 , 44749461)** | **5360.8 (4812.9 , 5915.5)** | **60.5 (54.3 , 67.3)** |
| **Andorra** | **1313 (1149 , 1486)** | **2271.4 (1995.7 , 2562.3)** | **5452 (4822 , 6146)** | **4070.1 (3594.5 , 4596.7)** | **79.2 (69.6 , 89.4)** |
| **Austria** | **230486 (207787 , 254222)** | **2075 (1866.2 , 2296)** | **608369 (537363 , 674266)** | **3839.5 (3378 , 4291.1)** | **85 (70.8 , 97.8)** |
| **Belgium** | **375620 (333465 , 416981)** | **2599.5 (2294.5 , 2896.3)** | **818787 (730733 , 908035)** | **4202 (3716.6 , 4686.5)** | **61.7 (50.4 , 76.1)** |
| **Cyprus** | **42011 (37908 , 46314)** | **4958.4 (4460 , 5475.9)** | **111583 (100949 , 122816)** | **5823.9 (5254.4 , 6449.1)** | **17.5 (8.5 , 27)** |
| **Denmark** | **136628 (122231 , 151619)** | **1874.6 (1668.5 , 2090.4)** | **358367 (323082 , 392890)** | **3641.5 (3261.8 , 4014.4)** | **94.3 (79.4 , 111)** |
| **Finland** | **240695 (220894 , 261514)** | **3545.1 (3248.5 , 3859.4)** | **578472 (520888 , 636778)** | **5892.7 (5270.9 , 6500.2)** | **66.2 (52.3 , 78.8)** |
| **France** | **1223043 (1111542 , 1334412)** | **1563.8 (1420.3 , 1707.9)** | **2889707 (2583225 , 3211293)** | **2487.6 (2199.8 , 2796.4)** | **59.1 (46.7 , 72.3)** |
| **Germany** | **5230171 (4705663 , 5855073)** | **4395.1 (3954.5 , 4915.8)** | **10536968 (9455168 , 11512652)** | **6632.7 (5963.9 , 7285.4)** | **50.9 (37.6 , 63.4)** |
| **Greece** | **377199 (338424 , 416237)** | **2576 (2305.2 , 2840.4)** | **854678 (757607 , 947326)** | **4442.4 (3937.6 , 4956.4)** | **72.5 (57.7 , 86.8)** |
| **Iceland** | **5951 (5261 , 6733)** | **2167.7 (1910.9 , 2453.4)** | **21323 (19067 , 23766)** | **4286.7 (3836.3 , 4775.3)** | **97.7 (85.5 , 111.1)** |
| **Ireland** | **57058 (50654 , 64035)** | **1459.7 (1293.3 , 1644.5)** | **286069 (254137 , 320941)** | **4083.2 (3624 , 4600.9)** | **179.7 (157.9 , 205.9)** |
| **Israel** | **136434 (122605 , 150646)** | **2858.1 (2568.4 , 3152.3)** | **514764 (465085 , 563450)** | **4741.9 (4284.7 , 5185.3)** | **65.9 (54.9 , 77.4)** |
| **Italy** | **3129787 (2789238 , 3467185)** | **3714.9 (3295.5 , 4119.8)** | **6372951 (5666595 , 7073822)** | **5301.9 (4660.2 , 5922.3)** | **42.7 (37.3 , 48.3)** |
| **Luxembourg** | **9470 (8506 , 10532)** | **1816 (1623.7 , 2025.5)** | **58997 (53222 , 65418)** | **6277.4 (5658.5 , 6966.7)** | **245.7 (220.2 , 275.2)** |
| **Malta** | **17962 (16216 , 19817)** | **4216.6 (3804.4 , 4655.5)** | **47914 (43158 , 52568)** | **5917.7 (5313.3 , 6553)** | **40.3 (30.9 , 50.9)** |
| **Monaco** | **1232 (1094 , 1376)** | **2067.5 (1822.7 , 2339.8)** | **3137 (2811 , 3500)** | **4077.8 (3617.5 , 4573.6)** | **97.2 (85.6 , 109.3)** |
| **Netherlands** | **496374 (446928 , 542040)** | **2583.1 (2318.6 , 2828.8)** | **1007365 (895486 , 1111944)** | **3384.1 (2986 , 3765)** | **31 (20.2 , 43.3)** |
| **Norway** | **208807 (187087 , 231585)** | **3483.2 (3098.7 , 3882.9)** | **403804 (361757 , 447512)** | **4813.8 (4290.7 , 5371.8)** | **38.2 (34.2 , 41.9)** |
| **Portugal** | **537414 (481227 , 595370)** | **3987.2 (3574.9 , 4411.2)** | **1283080 (1149936 , 1421289)** | **6307.3 (5611 , 7002.9)** | **58.2 (46 , 74.4)** |
| **San Marino** | **766 (679 , 866)** | **2425.6 (2148.2 , 2758.5)** | **2438 (2171 , 2741)** | **4424.1 (3916.4 , 4975.3)** | **82.4 (72 , 93.1)** |
| **Spain** | **2128839 (1950056 , 2297661)** | **3999.7 (3670.2 , 4323.9)** | **4478925 (4063655 , 4983022)** | **5439.9 (4890 , 6084.1)** | **36 (24.7 , 48)** |
| **Sweden** | **358297 (316932 , 400744)** | **2694.7 (2368.1 , 3040.5)** | **733954 (651329 , 813496)** | **4198.9 (3690.8 , 4692.6)** | **55.8 (44 , 69.4)** |
| **Switzerland** | **292315 (264001 , 321465)** | **2985 (2675.8 , 3299.1)** | **641985 (573883 , 722041)** | **4233.2 (3774.9 , 4779.6)** | **41.8 (30.7 , 54.2)** |
| **United Kingdom** | **2919462 (2634095 , 3227207)** | **3556.3 (3196.3 , 3937.3)** | **8115131 (7343654 , 8974234)** | **7948.8 (7120.8 , 8881.2)** | **123.5 (112.5 , 134.7)** |
| **Southern Latin America** | **1302515 (1193791 , 1410106)** | **2798.6 (2566.7 , 3028)** | **4069924 (3674650 , 4445697)** | **5031 (4528.4 , 5494.2)** | **79.8 (68.4 , 91.3)** |
| **Argentina** | **920101 (847459 , 998804)** | **2835.1 (2612.4 , 3073.3)** | **2542873 (2284577 , 2792432)** | **4865.4 (4368.3 , 5352.8)** | **71.6 (58.7 , 85.9)** |
| **Chile** | **343813 (308476 , 379764)** | **3341.1 (3003.8 , 3693.1)** | **1365507 (1231284 , 1518266)** | **5743.2 (5170.2 , 6383)** | **71.9 (58 , 88.6)** |
| **Uruguay** | **38548 (34127 , 43070)** | **995.1 (873 , 1117.9)** | **161339 (143730 , 178310)** | **3271.5 (2909.2 , 3630.4)** | **228.8 (198.6 , 266.8)** |
| **Eastern Europe** | **5879700 (5323106 , 6502304)** | **2167.6 (1966.9 , 2399.9)** | **9148099 (8294619 , 10074401)** | **2856.6 (2582.5 , 3157)** | **31.8 (28.1 , 35.6)** |
| **Belarus** | **287348 (258252 , 317715)** | **2284.9 (2051.7 , 2535.9)** | **386610 (341322 , 430065)** | **2641.6 (2323.2 , 2949.2)** | **15.6 (5 , 26.4)** |
| **Estonia** | **48718 (43703 , 53883)** | **2485.8 (2229.4 , 2755.7)** | **81002 (73238 , 89309)** | **3724.2 (3352.9 , 4117.6)** | **49.8 (40.6 , 60.4)** |
| **Latvia** | **80631 (72908 , 88786)** | **2376.4 (2145.1 , 2624.7)** | **124003 (112189 , 136383)** | **3755.4 (3380.5 , 4130.7)** | **58 (46.8 , 70.6)** |
| **Lithuania** | **97276 (88094 , 106930)** | **2228.1 (2016.6 , 2454.1)** | **139597 (126148 , 153520)** | **2936.1 (2632.3 , 3238.5)** | **31.8 (22.3 , 41.6)** |
| **Republic of Moldova** | **142928 (129443 , 157045)** | **3170.7 (2877.5 , 3481)** | **229993 (208037 , 254507)** | **4208.3 (3798.5 , 4677.2)** | **32.7 (21.3 , 44.4)** |
| **Russian Federation** | **3594733 (3231290 , 3974827)** | **2030.6 (1824.7 , 2249.3)** | **6050962 (5467935 , 6683099)** | **2732.5 (2456.7 , 3023.9)** | **34.6 (31.5 , 37.5)** |
| **Ukraine** | **1628066 (1451613 , 1823142)** | **2429.8 (2162.6 , 2727.8)** | **2135932 (1928884 , 2368249)** | **3125.6 (2804.3 , 3473.6)** | **28.6 (18.4 , 40)** |
| **Central Europe** | **5324949 (4863580 , 5786429)** | **3663.7 (3342.5 , 3984.4)** | **10798050 (9798146 , 11706176)** | **5619.9 (5099.5 , 6110.2)** | **53.4 (49.7 , 57.3)** |
| **Albania** | **44827 (39690 , 50482)** | **1988.3 (1770.9 , 2209.5)** | **125640 (111834 , 140033)** | **3140.2 (2798.8 , 3504.5)** | **57.9 (48.4 , 69)** |
| **Bosnia and Herzegovina** | **150096 (134857 , 166666)** | **3484.5 (3144.6 , 3859.8)** | **399382 (360150 , 442589)** | **7063.5 (6363.2 , 7829.5)** | **102.7 (89.1 , 116.2)** |
| **Bulgaria** | **408696 (369460 , 446832)** | **3407.2 (3092 , 3723.9)** | **628875 (567680 , 695822)** | **4974.9 (4494.7 , 5494.6)** | **46 (36.1 , 56.4)** |
| **Croatia** | **237810 (215868 , 260707)** | **3764.5 (3424.5 , 4119.6)** | **434595 (390007 , 484056)** | **5647.4 (5008 , 6298.6)** | **50 (40.3 , 61.9)** |
| **Czechia** | **633807 (571272 , 700476)** | **4780.5 (4302.2 , 5289.6)** | **1617707 (1464609 , 1768061)** | **8673.4 (7832 , 9529.7)** | **81.4 (69.1 , 93.3)** |
| **Hungary** | **530171 (474217 , 588223)** | **3781.9 (3384.7 , 4190.8)** | **983167 (885312 , 1079229)** | **5782.6 (5202.1 , 6368)** | **52.9 (41.1 , 65)** |
| **Montenegro** | **26196 (23654 , 28869)** | **4144.7 (3752.7 , 4563.9)** | **60738 (54629 , 66694)** | **6504 (5837.5 , 7138.1)** | **56.9 (48.2 , 66.8)** |
| **North Macedonia** | **79653 (71577 , 87553)** | **4120.8 (3709.8 , 4520.8)** | **231211 (208820 , 255347)** | **7254.6 (6563 , 8008.5)** | **76 (64.4 , 87.8)** |
| **Poland** | **1745189 (1587511 , 1903193)** | **4031.4 (3675 , 4398)** | **3543454 (3204724 , 3882317)** | **5672.1 (5109.8 , 6240.1)** | **40.7 (36.5 , 44.8)** |
| **Romania** | **720409 (641256 , 802633)** | **2617 (2338.2 , 2911.8)** | **1282495 (1140490 , 1423663)** | **3914.6 (3475.8 , 4351.5)** | **49.6 (38 , 60.7)** |
| **Serbia** | **474518 (426547 , 525645)** | **4142.7 (3735.7 , 4574.1)** | **935134 (838062 , 1019501)** | **6426.2 (5770.6 , 7031.4)** | **55.1 (44.3 , 67.5)** |
| **Slovakia** | **189347 (170398 , 209931)** | **3213.2 (2887.6 , 3557.5)** | **387182 (347926 , 427384)** | **4477.6 (4021.7 , 4942.4)** | **39.4 (30 , 49.8)** |
| **Slovenia** | **84229 (76350 , 93010)** | **3488.6 (3165 , 3846.2)** | **168471 (152237 , 185594)** | **4504.6 (4051.8 , 4992.5)** | **29.1 (19.9 , 37.8)** |
| **Central Asia** | **1393965 (1270741 , 1528425)** | **2754.4 (2518.9 , 3000.5)** | **4444184 (4078009 , 4824783)** | **5343 (4931.1 , 5771.4)** | **94 (86.4 , 102.6)** |
| **Armenia** | **88260 (80486 , 96606)** | **3026 (2761.4 , 3302.8)** | **215458 (196712 , 236867)** | **5336.2 (4884 , 5867.9)** | **76.3 (64.9 , 87)** |
| **Azerbaijan** | **149343 (134506 , 164190)** | **2695.7 (2437.1 , 2957.8)** | **564090 (502333 , 632824)** | **5265.9 (4731.3 , 5866.3)** | **95.3 (78.3 , 111.8)** |
| **Georgia** | **179188 (161528 , 197938)** | **2925.4 (2643.3 , 3238.3)** | **345872 (314662 , 377282)** | **6415.3 (5824.6 , 7025.9)** | **119.3 (101.9 , 136.3)** |
| **Kazakhstan** | **481295 (437425 , 532106)** | **3547 (3236.5 , 3909.3)** | **1134029 (1026603 , 1255103)** | **6156.4 (5581 , 6780.7)** | **73.6 (60.4 , 88.9)** |
| **Kyrgyzstan** | **69331 (62046 , 76629)** | **2152.8 (1938.1 , 2366.5)** | **172058 (152830 , 192118)** | **3268.7 (2903 , 3622.3)** | **51.8 (40.7 , 64.7)** |
| **Mongolia** | **16617 (14517 , 18867)** | **1380.7 (1215.7 , 1551)** | **62287 (54612 , 71158)** | **2174.5 (1924.3 , 2470.5)** | **57.5 (49.3 , 66.7)** |
| **Tajikistan** | **79562 (72134 , 87731)** | **2530.4 (2296.1 , 2773.3)** | **354713 (318605 , 392596)** | **5505.9 (5004.6 , 6058.5)** | **117.6 (99.8 , 133.7)** |
| **Turkmenistan** | **55806 (49496 , 62701)** | **2538.9 (2258.4 , 2824.9)** | **213269 (192591 , 234686)** | **4749.6 (4295.1 , 5215.5)** | **87.1 (74 , 100.7)** |
| **Uzbekistan** | **274563 (245620 , 306006)** | **2141.8 (1932.6 , 2358.5)** | **1382407 (1259994 , 1508879)** | **5270.3 (4832.1 , 5731.8)** | **146.1 (128.2 , 167.3)** |
| **Central Latin America** | **6052152 (5580476 , 6547024)** | **6507.7 (6018 , 7003.3)** | **20824339 (19192738 , 22360008)** | **8505.6 (7837.2 , 9133.4)** | **30.7 (27 , 34.3)** |
| **Colombia** | **1054739 (950384 , 1171391)** | **5422.4 (4880 , 5995.1)** | **3551204 (3229892 , 3887271)** | **6746.9 (6135.1 , 7385.1)** | **24.4 (13.9 , 34.6)** |
| **Costa Rica** | **92365 (83663 , 101417)** | **4900.6 (4450.6 , 5378.2)** | **377378 (338255 , 415540)** | **7256.1 (6505.2 , 7985.1)** | **48.1 (37.1 , 59.7)** |
| **El Salvador** | **122779 (111866 , 133453)** | **3816.5 (3463.9 , 4155.3)** | **441050 (402608 , 480172)** | **7458.2 (6801.6 , 8136.9)** | **95.4 (82.9 , 107.2)** |
| **Guatemala** | **172269 (156814 , 187762)** | **4170.4 (3824.3 , 4545.9)** | **1082309 (991416 , 1166017)** | **8784.6 (8076.8 , 9535.3)** | **110.6 (94.6 , 126.8)** |
| **Honduras** | **125502 (112717 , 139033)** | **5365.4 (4828 , 5954)** | **565312 (508084 , 627526)** | **8222.5 (7387.6 , 9114.1)** | **53.3 (44.1 , 62.4)** |
| **Mexico** | **3786616 (3483822 , 4101007)** | **7930.1 (7305.5 , 8559.4)** | **11931554 (10941718 , 12850481)** | **9710.4 (8919.6 , 10456.8)** | **22.5 (17.7 , 28)** |
| **Nicaragua** | **91413 (83435 , 100751)** | **5185.5 (4742.4 , 5686.3)** | **393508 (359447 , 428083)** | **7845 (7172.2 , 8508.9)** | **51.3 (41.5 , 62.8)** |
| **Panama** | **73550 (66596 , 80984)** | **4602.4 (4164.9 , 5066.8)** | **319652 (289604 , 349031)** | **7671 (6949.9 , 8372.1)** | **66.7 (54.4 , 80.5)** |
| **Venezuela (Bolivarian Republic of)** | **532918 (484706 , 583561)** | **4930.8 (4469.2 , 5393.9)** | **2162373 (1980757 , 2355141)** | **7222.7 (6628.6 , 7846.8)** | **46.5 (36.5 , 58.3)** |
| **Andean Latin America** | **553488 (505577 , 600964)** | **2524.1 (2315.5 , 2729.1)** | **2443455 (2239816 , 2652085)** | **4250.7 (3898.4 , 4602.7)** | **68.4 (61.9 , 75.2)** |
| **Bolivia (Plurinational State of)** | **109900 (98077 , 122289)** | **3154.7 (2809.8 , 3525.9)** | **446442 (402357 , 494721)** | **4783.1 (4312.9 , 5293.4)** | **51.6 (43.1 , 60.1)** |
| **Ecuador** | **175992 (160522 , 192449)** | **3065.7 (2790.4 , 3336.5)** | **850140 (772256 , 922170)** | **5460.5 (4973.1 , 5924.7)** | **78.1 (66.6 , 88.2)** |
| **Peru** | **267596 (240954 , 294517)** | **2109.6 (1911.8 , 2311.4)** | **1146872 (1039202 , 1257301)** | **3517.4 (3186.1 , 3858.5)** | **66.7 (55.9 , 77.6)** |
| **Caribbean** | **1437301 (1335167 , 1548089)** | **5253.4 (4886.2 , 5650.4)** | **3848753 (3562148 , 4157743)** | **7479.2 (6913.6 , 8083.3)** | **42.4 (37.2 , 48.3)** |
| **Antigua and Barbuda** | **2949 (2714 , 3218)** | **5631.9 (5158.5 , 6190.9)** | **8809 (7983 , 9689)** | **8405.1 (7656.4 , 9217)** | **49.2 (39.8 , 58.4)** |
| **Barbados** | **15147 (14060 , 16254)** | **5540.1 (5129.3 , 5971.1)** | **35013 (32140 , 38104)** | **7715.7 (7093.5 , 8413.1)** | **39.3 (30.4 , 48.3)** |
| **Belize** | **3759 (3429 , 4097)** | **3684.3 (3368.4 , 4023.3)** | **20242 (18387 , 22197)** | **6435.4 (5857.2 , 7024.9)** | **74.7 (63.1 , 86.8)** |
| **Bermuda** | **2223 (2023 , 2440)** | **3455.5 (3143.1 , 3785.9)** | **5611 (5057 , 6193)** | **4918 (4437.4 , 5416.4)** | **42.3 (34.2 , 51.6)** |
| **Bahamas** | **9130 (8283 , 10091)** | **5234.4 (4744.5 , 5756.3)** | **32256 (29281 , 35511)** | **7796.2 (7093.3 , 8538.3)** | **48.9 (39.1 , 58.9)** |
| **Cuba** | **566921 (517835 , 621711)** | **5425.8 (4957.2 , 5939.6)** | **1225468 (1112382 , 1351323)** | **7040.6 (6395.6 , 7763.8)** | **29.8 (20.4 , 40.2)** |
| **Dominica** | **3913 (3588 , 4284)** | **5836.4 (5334 , 6411.6)** | **8765 (7992 , 9624)** | **10188.8 (9296.2 , 11171.5)** | **74.6 (64.5 , 84.7)** |
| **Dominican Republic** | **102423 (93370 , 112408)** | **2374.5 (2169.9 , 2597)** | **432246 (395382 , 473662)** | **4384.4 (4005.6 , 4801.5)** | **84.6 (73.1 , 96.9)** |
| **Grenada** | **4104 (3777 , 4459)** | **5987.5 (5513 , 6535.9)** | **10852 (9924 , 11853)** | **9388.4 (8612.3 , 10230.9)** | **56.8 (48 , 66.7)** |
| **Guyana** | **30786 (28047 , 33742)** | **6913.6 (6313.1 , 7563.8)** | **74218 (68046 , 80687)** | **10782.4 (9910.1 , 11679)** | **56 (46.4 , 68)** |
| **Haiti** | **217286 (195859 , 239906)** | **5730.4 (5170.5 , 6342.4)** | **728907 (654820 , 806066)** | **8745.1 (7881.5 , 9685.2)** | **52.6 (45.9 , 60.3)** |
| **Jamaica** | **85722 (79180 , 92498)** | **4825.1 (4443.7 , 5221.5)** | **253669 (231703 , 275970)** | **8492.8 (7756.2 , 9233.1)** | **76 (63.2 , 88.1)** |
| **Puerto Rico** | **231160 (211336 , 254637)** | **6404.3 (5856.4 , 7054.2)** | **560737 (513309 , 609120)** | **9442.6 (8635.6 , 10253.7)** | **47.4 (37.5 , 59.6)** |
| **Saint Kitts and Nevis** | **2225 (2035 , 2426)** | **6285.7 (5754.2 , 6867.4)** | **5876 (5339 , 6481)** | **8378.4 (7652.9 , 9189.4)** | **33.3 (25.1 , 40.9)** |
| **Saint Lucia** | **7768 (7155 , 8474)** | **8502.7 (7801.9 , 9297.4)** | **24203 (22167 , 26372)** | **11197.4 (10265.1 , 12173.4)** | **31.7 (23.3 , 40)** |
| **Saint Vincent and the Grenadines** | **5157 (4735 , 5609)** | **6960.8 (6385.3 , 7585.7)** | **13698 (12573 , 14911)** | **10154.3 (9339.9 , 11037)** | **45.9 (36.6 , 56.3)** |
| **Suriname** | **16871 (15320 , 18516)** | **5972.7 (5420.9 , 6561.6)** | **64148 (58778 , 69849)** | **10360.1 (9521.4 , 11231.4)** | **73.5 (64.2 , 83.5)** |
| **Trinidad and Tobago** | **77453 (70371 , 83980)** | **8740.7 (7920 , 9491)** | **200165 (184133 , 219153)** | **10897 (10024.5 , 11896.7)** | **24.7 (12.3 , 37.2)** |
| **United States Virgin Islands** | **4424 (3985 , 4906)** | **4791.8 (4331.9 , 5272.5)** | **13493 (12218 , 14991)** | **8092.6 (7346.8 , 8966.4)** | **68.9 (59.5 , 78.9)** |
| **Tropical Latin America** | **4355643 (3982988 , 4739345)** | **4351.8 (3988.1 , 4716.8)** | **12302104 (11179011 , 13438075)** | **4979.1 (4525.8 , 5443.8)** | **14.4 (11.2 , 18.1)** |
| **Brazil** | **4281780 (3913093 , 4658149)** | **4383.1 (4016.3 , 4749.8)** | **12007018 (10906961 , 13128882)** | **4977.8 (4523 , 5445)** | **13.6 (10.2 , 17.3)** |
| **Paraguay** | **73863 (66525 , 81097)** | **3086.5 (2791.8 , 3383.7)** | **295085 (267649 , 325748)** | **5070.8 (4606.5 , 5582.3)** | **64.3 (53 , 77)** |
| **East Asia** | **35806794 (32149407 , 39877899)** | **3653.8 (3299.6 , 4032)** | **93251505 (85377465 , 101871859)** | **4502.3 (4110.8 , 4930)** | **23.2 (16.3 , 29.8)** |
| **China** | **34667456 (31084397 , 38665214)** | **3669.1 (3308.9 , 4056.1)** | **90017578 (82336589 , 98508874)** | **4496.4 (4097.9 , 4928.8)** | **22.5 (15.5 , 29.3)** |
| **Democratic People's Republic of Korea** | **495699 (443636 , 554530)** | **2782 (2507.3 , 3100.4)** | **1380706 (1237081 , 1527854)** | **4271.2 (3833.2 , 4724)** | **53.5 (45.2 , 62.2)** |
| **Taiwan (Province of China)** | **643639 (599158 , 695791)** | **3733.9 (3482.6 , 4014.4)** | **1853220 (1751755 , 1961557)** | **4879.5 (4591.4 , 5166.8)** | **30.7 (23.7 , 39)** |
| **Southeast Asia** | **8677020 (7923910 , 9510256)** | **3051.8 (2790.3 , 3341.6)** | **31222039 (28746774 , 33964768)** | **4875.1 (4493.5 , 5285.6)** | **59.7 (54.9 , 64.7)** |
| **Cambodia** | **117758 (106708 , 130304)** | **2314.7 (2101.3 , 2555.6)** | **702652 (635402 , 775219)** | **5504.4 (4995.8 , 6071.6)** | **137.8 (124.5 , 151.9)** |
| **Indonesia** | **2933481 (2638537 , 3249353)** | **2579.8 (2333.3 , 2856.6)** | **10076892 (9104852 , 11121542)** | **4168.3 (3768.3 , 4595.1)** | **61.6 (56.1 , 67.5)** |
| **Lao People's Democratic Republic** | **85596 (77025 , 94060)** | **3733 (3362.9 , 4112.8)** | **307268 (276531 , 341931)** | **6273.6 (5682.5 , 6909.1)** | **68.1 (60.2 , 76.4)** |
| **Malaysia** | **456119 (421245 , 493864)** | **4369.1 (4055.3 , 4692.8)** | **1814552 (1631971 , 2002846)** | **6399.5 (5748.1 , 7051.7)** | **46.5 (36.4 , 55.4)** |
| **Maldives** | **3169 (2844 , 3504)** | **3243.4 (2932.2 , 3556.1)** | **17963 (16105 , 19979)** | **4870.9 (4387.7 , 5361.7)** | **50.2 (41.9 , 58.3)** |
| **Mauritius** | **45177 (41848 , 48579)** | **5589.2 (5176.3 , 6011.7)** | **188882 (172179 , 204328)** | **10846.5 (9954.6 , 11722)** | **94.1 (82.4 , 107.6)** |
| **Myanmar** | **980685 (885520 , 1084990)** | **3837.2 (3470.1 , 4238.3)** | **2913712 (2702321 , 3151289)** | **5973.3 (5527.9 , 6446.9)** | **55.7 (46.1 , 66.3)** |
| **Philippines** | **1205144 (1098695 , 1321321)** | **3499.8 (3194.2 , 3821.4)** | **3655605 (3340742 , 3983744)** | **4342.9 (3985.5 , 4718.8)** | **24.1 (19.4 , 29.3)** |
| **Sri Lanka** | **491913 (449640 , 537977)** | **4276.7 (3915.8 , 4643.5)** | **2347597 (2144174 , 2561192)** | **9171.2 (8386.3 , 9993.7)** | **114.4 (101.2 , 129.2)** |
| **Seychelles** | **2337 (2113 , 2571)** | **4071.5 (3680.5 , 4479.2)** | **10935 (9900 , 12094)** | **9605.1 (8773 , 10531.6)** | **135.9 (124.2 , 149.6)** |
| **Thailand** | **1216382 (1106658 , 1335886)** | **3084.7 (2812.2 , 3378)** | **4751798 (4346870 , 5205472)** | **4733.8 (4329.9 , 5194.3)** | **53.5 (41.3 , 66.7)** |
| **Timor-Leste** | **8679 (7800 , 9639)** | **2479.5 (2230.8 , 2748.8)** | **42595 (38513 , 46723)** | **4992.6 (4502.7 , 5477.3)** | **101.4 (91.4 , 112)** |
| **Viet Nam** | **1119045 (1013020 , 1232949)** | **2673 (2425.7 , 2949.8)** | **4350684 (3992508 , 4738162)** | **4567 (4184.4 , 4958.4)** | **70.9 (61.6 , 80.6)** |
| **Oceania** | **254116 (230984 , 280448)** | **6619.6 (6054.8 , 7260.3)** | **1021936 (935451 , 1121700)** | **11086.1 (10196.7 , 12087.4)** | **67.5 (61.7 , 73.5)** |
| **American Samoa** | **3399 (3094 , 3781)** | **11722.1 (10719.3 , 12865.4)** | **10228 (9343 , 11255)** | **19876.8 (18211.1 , 21795.3)** | **69.6 (60 , 78.9)** |
| **Cook Islands** | **963 (879 , 1059)** | **6945.3 (6341.6 , 7605)** | **2912 (2671 , 3173)** | **12652 (11600.7 , 13829.6)** | **82.2 (71.8 , 94)** |
| **Micronesia (Federated States of)** | **3156 (2865 , 3478)** | **5568.9 (5084.2 , 6107.9)** | **10245 (9323 , 11331)** | **12220.6 (11129.4 , 13417.2)** | **119.4 (108.2 , 132.1)** |
| **Fiji** | **40624 (36968 , 44942)** | **9012.1 (8262.2 , 9879.1)** | **133370 (123374 , 144474)** | **15960.3 (14887.5 , 17141.5)** | **77.1 (66.8 , 88.5)** |
| **Guam** | **5057 (4589 , 5669)** | **5174.6 (4721 , 5740)** | **14887 (13555 , 16477)** | **8009.3 (7306.2 , 8856.6)** | **54.8 (46.5 , 63)** |
| **Kiribati** | **3531 (3209 , 3893)** | **7475.1 (6841 , 8209)** | **11432 (10375 , 12673)** | **13023.5 (11884.1 , 14337)** | **74.2 (64.4 , 83.2)** |
| **Marshall Islands** | **2375 (2139 , 2651)** | **11022.4 (9957.5 , 12154.9)** | **8804 (7984 , 9826)** | **19523.9 (17853.4 , 21563.3)** | **77.1 (69.4 , 86.2)** |
| **Nauru** | **320 (289 , 355)** | **5932.1 (5406.2 , 6498.5)** | **703 (634 , 779)** | **10961.1 (10008.9 , 12007.3)** | **84.8 (75.6 , 94.2)** |
| **Niue** | **183 (166 , 202)** | **8750.7 (7942.4 , 9674.1)** | **335 (308 , 367)** | **16454.2 (15067.8 , 18027.2)** | **88 (78.9 , 98.7)** |
| **Northern Mariana Islands** | **1759 (1576 , 1986)** | **5968.7 (5424.8 , 6568.3)** | **5731 (5191 , 6337)** | **10270.2 (9386.9 , 11276.9)** | **72.1 (63.4 , 81)** |
| **Palau** | **808 (736 , 891)** | **7035.4 (6420.3 , 7730.7)** | **3127 (2853 , 3474)** | **13400.5 (12286.2 , 14763.5)** | **90.5 (81.9 , 99.7)** |
| **Papua New Guinea** | **152860 (137868 , 169985)** | **6237.4 (5673.9 , 6893.6)** | **677666 (613635 , 748070)** | **10224 (9308.2 , 11242.5)** | **63.9 (56.1 , 72.1)** |
| **Samoa** | **6396 (5857 , 6996)** | **6485.5 (5947.6 , 7102)** | **18301 (16637 , 20196)** | **11281.3 (10254.8 , 12397.3)** | **73.9 (65 , 82.2)** |
| **Solomon Islands** | **9445 (8488 , 10499)** | **5278.4 (4767.4 , 5838.9)** | **44478 (40565 , 49138)** | **10588.8 (9711 , 11571.4)** | **100.6 (89.4 , 111.4)** |
| **Tokelau** | **80 (73 , 88)** | **6122.9 (5555.3 , 6739.9)** | **148 (135 , 164)** | **11074.5 (10107.3 , 12252.9)** | **80.9 (72.3 , 90.1)** |
| **Tonga** | **3951 (3596 , 4354)** | **6452.7 (5893.9 , 7089.4)** | **8706 (7959 , 9528)** | **10513.2 (9629.8 , 11489.1)** | **62.9 (54.9 , 70.9)** |
| **Tuvalu** | **464 (420 , 513)** | **6216 (5650.9 , 6855.3)** | **1261 (1140 , 1398)** | **11643.4 (10557.9 , 12902.5)** | **87.3 (79.3 , 96)** |
| **Vanuatu** | **4682 (4185 , 5255)** | **5672.6 (5097.4 , 6327.3)** | **21336 (19355 , 23522)** | **10478.5 (9534.3 , 11518.1)** | **84.7 (74.9 , 95.6)** |
| **North Africa and Middle East** | **6959611 (6320252 , 7662966)** | **3640.4 (3313.8 , 3996)** | **32927679 (29948035 , 36227971)** | **6753.3 (6170.2 , 7394.2)** | **85.5 (80.8 , 90.3)** |
| **Afghanistan** | **330512 (295972 , 368753)** | **4458.7 (4001.2 , 4972.7)** | **1389719 (1229482 , 1569720)** | **8537.4 (7658.3 , 9562.5)** | **91.5 (82.3 , 100.1)** |
| **Algeria** | **546325 (488835 , 610518)** | **4048 (3641.2 , 4517.6)** | **2810041 (2505864 , 3150617)** | **7675.1 (6860.5 , 8599.1)** | **89.6 (78.4 , 101.2)** |
| **Bahrain** | **16561 (14839 , 18328)** | **7546 (6822.4 , 8259.7)** | **187048 (169696 , 207117)** | **14234.9 (13261.9 , 15287.2)** | **88.6 (73.6 , 103.5)** |
| **Egypt** | **775872 (699913 , 860055)** | **2392.2 (2172.6 , 2629.7)** | **4082798 (3672577 , 4558285)** | **5657.6 (5105.8 , 6302.3)** | **136.5 (119.8 , 154.2)** |
| **Iran (Islamic Republic of)** | **974556 (867290 , 1092764)** | **3285.6 (2940.8 , 3652.4)** | **5035012 (4514277 , 5574025)** | **6312.9 (5690.2 , 6959.5)** | **92.1 (85.4 , 99.6)** |
| **Iraq** | **499111 (449622 , 550396)** | **5595.5 (5041.5 , 6185.8)** | **2330391 (2127199 , 2547889)** | **8564.6 (7852.9 , 9298.9)** | **53.1 (41.1 , 65.7)** |
| **Jordan** | **81163 (74323 , 89072)** | **5243.6 (4824.3 , 5714.5)** | **592099 (534664 , 652247)** | **7628.8 (6944.1 , 8318)** | **45.5 (35 , 55.9)** |
| **Kuwait** | **58101 (51753 , 64787)** | **6685.2 (6107.3 , 7339.9)** | **375781 (332354 , 428212)** | **10250.1 (9240.2 , 11339.5)** | **53.3 (42.1 , 63.9)** |
| **Lebanon** | **106905 (96886 , 118263)** | **4447.1 (4035 , 4924.8)** | **401584 (360406 , 448278)** | **7653.3 (6871 , 8524.3)** | **72.1 (61.2 , 82)** |
| **Libya** | **99974 (89656 , 110772)** | **4770.4 (4282.1 , 5283.8)** | **545926 (486543 , 612688)** | **9292 (8328.3 , 10384.7)** | **94.8 (83 , 105.6)** |
| **Morocco** | **515583 (459272 , 574640)** | **3419.6 (3061.9 , 3803.5)** | **2331322 (2085396 , 2596446)** | **6918 (6211.8 , 7684)** | **102.3 (91.7 , 113.5)** |
| **Palestine** | **42912 (38612 , 47562)** | **4569.7 (4130.8 , 5053)** | **225249 (203113 , 245840)** | **8319.6 (7549.9 , 9013)** | **82.1 (67.8 , 97.1)** |
| **Oman** | **36478 (32624 , 40863)** | **4248.8 (3825.3 , 4693.4)** | **192772 (170019 , 219061)** | **7423.3 (6666.4 , 8199.8)** | **74.7 (64.5 , 84.7)** |
| **Qatar** | **14781 (13050 , 16832)** | **8552.6 (7728.4 , 9442.2)** | **274291 (244493 , 309556)** | **16312.4 (15050 , 17723.2)** | **90.7 (75.2 , 107.8)** |
| **Saudi Arabia** | **394590 (358027 , 434591)** | **5380.7 (4914.3 , 5869.4)** | **2486355 (2216629 , 2808991)** | **9453.1 (8563.1 , 10498.6)** | **75.7 (65.4 , 87.1)** |
| **Sudan** | **372731 (332103 , 417992)** | **3621.7 (3234.6 , 4040)** | **1592344 (1420144 , 1790129)** | **7272 (6509.8 , 8134.3)** | **100.8 (91.6 , 110.8)** |
| **Syrian Arab Republic** | **260867 (236014 , 290479)** | **4341.8 (3942.2 , 4797.3)** | **906551 (806813 , 1009546)** | **6832.5 (6133.8 , 7554.8)** | **57.4 (47.8 , 67.7)** |
| **Tunisia** | **234482 (210287 , 259720)** | **4352.6 (3907.5 , 4816.6)** | **1061155 (950830 , 1181080)** | **8162.2 (7324.5 , 9059.8)** | **87.5 (78.5 , 97.1)** |
| **Turkey** | **1396535 (1276620 , 1529149)** | **3582.8 (3284.4 , 3912.6)** | **4614950 (4151327 , 5055521)** | **5082 (4584.6 , 5564.4)** | **41.8 (29.5 , 54.5)** |
| **United Arab Emirates** | **43887 (38439 , 49682)** | **6855.8 (6171.6 , 7585.2)** | **719240 (625472 , 821821)** | **11098.2 (10089 , 12249.4)** | **61.9 (52.2 , 72.8)** |
| **Yemen** | **153005 (135510 , 172394)** | **2720.2 (2418.6 , 3042)** | **739598 (652478 , 833372)** | **4686.9 (4150.6 , 5245.5)** | **72.3 (63.6 , 82)** |
| **South Asia** | **25337429 (22623586 , 28422675)** | **3790.9 (3402.6 , 4205)** | **98136964 (88391917 , 108843988)** | **6375.2 (5752.2 , 7067.8)** | **68.2 (64.2 , 72.1)** |
| **Bangladesh** | **1773156 (1610378 , 1945357)** | **3139.7 (2866.6 , 3439.6)** | **6458462 (5929591 , 7070819)** | **4588.3 (4226.2 , 5006.6)** | **46.1 (39 , 53.5)** |
| **Bhutan** | **9311 (8279 , 10433)** | **2997.2 (2677.9 , 3326.1)** | **32153 (29198 , 35390)** | **5111.5 (4660.3 , 5609.4)** | **70.5 (62.5 , 79.4)** |
| **India** | **20787242 (18449001 , 23430262)** | **3878.7 (3462.8 , 4311.1)** | **81850597 (73601998 , 91024198)** | **6605.5 (5949.4 , 7342.3)** | **70.3 (65.8 , 74.6)** |
| **Nepal** | **333501 (297022 , 374844)** | **3005.1 (2697.4 , 3332.4)** | **1350744 (1211777 , 1501682)** | **5524.1 (4969.3 , 6120)** | **83.8 (74.8 , 93)** |
| **Pakistan** | **2434219 (2168210 , 2722429)** | **3775.5 (3385.5 , 4203.1)** | **8445008 (7538820 , 9482314)** | **6273 (5629.4 , 7003.9)** | **66.1 (60.1 , 72.7)** |
| **Southern Sub-Saharan Africa** | **1038467 (935213 , 1148487)** | **3481.2 (3157.6 , 3820.7)** | **3389939 (3098493 , 3696155)** | **5605.7 (5133.9 , 6083)** | **61 (55.1 , 68.1)** |
| **Botswana** | **20992 (18717 , 23423)** | **3339.6 (3004.5 , 3694.4)** | **87824 (78753 , 97404)** | **5685.4 (5139.8 , 6264.5)** | **70.2 (60.4 , 80.9)** |
| **Lesotho** | **29720 (26493 , 33378)** | **2883 (2581.7 , 3225.5)** | **71058 (64007 , 78903)** | **5026.1 (4548.1 , 5576.9)** | **74.3 (64.2 , 85.4)** |
| **Namibia** | **26466 (23796 , 29414)** | **3464.9 (3121.3 , 3838.1)** | **69688 (62535 , 77359)** | **4535.7 (4079.1 , 5034.1)** | **30.9 (23.4 , 39.2)** |
| **South Africa** | **793701 (716183 , 879209)** | **3519.7 (3191.9 , 3876.2)** | **2658457 (2435602 , 2893910)** | **5610.2 (5149.2 , 6091.5)** | **59.4 (52.5 , 67.7)** |
| **Eswatini** | **11809 (10594 , 13202)** | **3605.7 (3253.3 , 3999.4)** | **37994 (34261 , 41762)** | **5846.8 (5302.3 , 6410.5)** | **62.2 (52.8 , 72.4)** |
| **Zimbabwe** | **155779 (138113 , 172958)** | **3436.8 (3087 , 3798.9)** | **464917 (415848 , 513378)** | **5784.7 (5174.9 , 6407)** | **68.3 (58.1 , 79.4)** |
| **Western Sub-Saharan Africa** | **2021192 (1802277 , 2250337)** | **2156.3 (1930.7 , 2383.7)** | **6980587 (6275393 , 7737550)** | **3279.9 (2962.2 , 3612.2)** | **52.1 (49 , 55.5)** |
| **Benin** | **52452 (46508 , 58097)** | **2439.7 (2180 , 2704.7)** | **232795 (208583 , 258796)** | **4141.6 (3729.6 , 4575)** | **69.8 (60.6 , 79.5)** |
| **Burkina Faso** | **118726 (105548 , 131858)** | **2575.5 (2305.4 , 2851)** | **402196 (362622 , 444673)** | **3833.5 (3460.5 , 4214.8)** | **48.8 (40.7 , 57.9)** |
| **Cameroon** | **103399 (91991 , 115617)** | **2154.5 (1926.4 , 2387.3)** | **519445 (467655 , 577584)** | **3784.2 (3417 , 4174)** | **75.6 (66 , 86.2)** |
| **Cabo Verde** | **5948 (5343 , 6618)** | **2591 (2319 , 2875.9)** | **23095 (20688 , 25633)** | **5144.9 (4614.6 , 5691.3)** | **98.6 (86.5 , 111.1)** |
| **Chad** | **70302 (62494 , 78287)** | **2370.7 (2112.4 , 2633.6)** | **247195 (221291 , 273272)** | **3842.6 (3440 , 4243.2)** | **62.1 (53.4 , 71.1)** |
| **CÃ´te d'Ivoire** | **114986 (102048 , 129876)** | **2439.5 (2187.1 , 2704.8)** | **511925 (461316 , 565395)** | **4072.1 (3692.8 , 4459.4)** | **66.9 (57.8 , 76.1)** |
| **Gambia** | **8334 (7378 , 9375)** | **2105.4 (1885.2 , 2353.8)** | **39445 (35548 , 43755)** | **3629.3 (3276.1 , 4009.4)** | **72.4 (63.2 , 81.5)** |
| **Ghana** | **179972 (159906 , 201948)** | **2602.6 (2336.2 , 2909.1)** | **815402 (732480 , 902683)** | **4468.6 (4038.4 , 4954)** | **71.7 (62.5 , 81.8)** |
| **Guinea** | **68807 (61479 , 76631)** | **2001.8 (1800.1 , 2221)** | **214909 (193531 , 238199)** | **3522.8 (3173.9 , 3880.9)** | **76 (67.3 , 86.6)** |
| **Guinea-Bissau** | **11590 (10397 , 12812)** | **2572.7 (2325.8 , 2843.6)** | **35507 (31883 , 39679)** | **4072.9 (3658.9 , 4522.9)** | **58.3 (51.1 , 66.8)** |
| **Liberia** | **35308 (31671 , 39330)** | **3079 (2768.5 , 3412.4)** | **117182 (104808 , 130996)** | **4865.3 (4347.1 , 5419.4)** | **58 (49.9 , 66.4)** |
| **Mali** | **97273 (86661 , 108389)** | **2217.1 (1978.6 , 2458.6)** | **345125 (310852 , 381747)** | **3570.3 (3222.5 , 3952.8)** | **61 (52.4 , 70.5)** |
| **Mauritania** | **18603 (16431 , 20767)** | **1766.9 (1576.4 , 1967.3)** | **58665 (52512 , 65396)** | **2615.1 (2355.5 , 2902.6)** | **48 (39.4 , 56.7)** |
| **Niger** | **34948 (30499 , 39569)** | **1153.8 (1029.7 , 1289.1)** | **224647 (199273 , 251680)** | **2553.7 (2268.8 , 2832.8)** | **121.3 (107.3 , 137)** |
| **Nigeria** | **926547 (820482 , 1036034)** | **1997.3 (1784.6 , 2221.4)** | **2537040 (2251378 , 2846075)** | **2615.8 (2339.6 , 2897.3)** | **31 (28.1 , 34.2)** |
| **Sao Tome and Principe** | **1814 (1609 , 2028)** | **2757 (2465.2 , 3077.3)** | **5039 (4484 , 5606)** | **4264.5 (3812.1 , 4726.7)** | **54.7 (45.5 , 63.1)** |
| **Senegal** | **124324 (111468 , 138151)** | **3555.6 (3194 , 3938.1)** | **441228 (396815 , 489679)** | **5320.6 (4779 , 5915.3)** | **49.6 (42.3 , 57.8)** |
| **Sierra Leone** | **24255 (21452 , 27131)** | **1217.3 (1096.3 , 1351.7)** | **98389 (87943 , 109200)** | **2420.3 (2177.4 , 2667)** | **98.8 (88.1 , 110.7)** |
| **Togo** | **23536 (20568 , 26545)** | **1691.5 (1502.4 , 1872.9)** | **111261 (99262 , 124446)** | **2683.6 (2417.7 , 2969.1)** | **58.7 (50.6 , 68.2)** |
| **Eastern Sub-Saharan Africa** | **2048340 (1833458 , 2284045)** | **2362.4 (2135.5 , 2604.5)** | **6067734 (5427037 , 6771359)** | **3052.3 (2760.6 , 3377.6)** | **29.2 (26.5 , 32.2)** |
| **Burundi** | **66885 (59541 , 74438)** | **2480.2 (2224.4 , 2755.8)** | **173701 (155044 , 195778)** | **3118.4 (2818.9 , 3467.9)** | **25.7 (19.2 , 32.3)** |
| **Comoros** | **5160 (4613 , 5690)** | **2117.4 (1907.4 , 2326.9)** | **14791 (13388 , 16407)** | **2789.9 (2531.7 , 3081.1)** | **31.8 (24.9 , 39)** |
| **Djibouti** | **4256 (3767 , 4818)** | **2297.8 (2063.3 , 2560.5)** | **24656 (22015 , 27551)** | **3337.1 (3006.2 , 3686.8)** | **45.2 (38.7 , 52.8)** |
| **Eritrea** | **30970 (27447 , 34995)** | **2435.3 (2195.1 , 2700.3)** | **118362 (105166 , 132462)** | **3432.2 (3085.1 , 3807.9)** | **40.9 (33.8 , 48.8)** |
| **Ethiopia** | **633550 (565029 , 710554)** | **2680.8 (2409.8 , 2971.7)** | **1237819 (1093123 , 1390074)** | **2476.5 (2214.8 , 2744.6)** | **-7.6 (-10.5 , -4.7)** |
| **Kenya** | **187557 (165564 , 210825)** | **1983.4 (1769.2 , 2200.5)** | **761313 (675816 , 858738)** | **2811.9 (2518.2 , 3123.5)** | **41.8 (38.3 , 45.8)** |
| **Madagascar** | **129583 (115901 , 144548)** | **2208.5 (1989.2 , 2455)** | **411904 (367849 , 461758)** | **3014.4 (2701.4 , 3339.4)** | **36.5 (29.9 , 43.7)** |
| **Malawi** | **123811 (109847 , 139115)** | **2793.2 (2507.9 , 3101.6)** | **344197 (307607 , 385222)** | **3889 (3513.4 , 4325.1)** | **39.2 (31.7 , 47.4)** |
| **Mozambique** | **158058 (141434 , 176683)** | **2360.8 (2134.9 , 2613.6)** | **501854 (450844 , 555200)** | **3696.6 (3342.7 , 4073.8)** | **56.6 (48.6 , 65.6)** |
| **Rwanda** | **82011 (73089 , 92093)** | **2432 (2178.3 , 2699.4)** | **205511 (182980 , 228472)** | **2911.2 (2617.7 , 3219.8)** | **19.7 (13.9 , 26.5)** |
| **Somalia** | **76394 (67930 , 85728)** | **2452.1 (2200 , 2710.5)** | **295140 (263710 , 333324)** | **3416.1 (3081.9 , 3790.5)** | **39.3 (32.4 , 47.8)** |
| **South Sudan** | **61614 (54802 , 68843)** | **2273.6 (2045.4 , 2526)** | **145891 (131054 , 163534)** | **3200.8 (2882.2 , 3557.1)** | **40.8 (33.5 , 48.9)** |
| **United Republic of Tanzania** | **193236 (170728 , 216485)** | **1606.6 (1447 , 1777.1)** | **820110 (736210 , 912985)** | **2847.3 (2565.3 , 3157.3)** | **77.2 (67.5 , 87.9)** |
| **Uganda** | **205608 (183502 , 230335)** | **2779.5 (2486.3 , 3082.8)** | **711131 (633287 , 795258)** | **4065.7 (3635.8 , 4514.4)** | **46.3 (38.8 , 53.5)** |
| **Zambia** | **88145 (78284 , 98486)** | **2604.5 (2336.5 , 2890.7)** | **296505 (264897 , 331609)** | **3522.6 (3174.1 , 3902.3)** | **35.3 (27.9 , 43.1)** |
| **Central Sub-Saharan Africa** | **923871 (829052 , 1032764)** | **3488.5 (3160 , 3865.8)** | **3289301 (2945368 , 3654551)** | **4955.9 (4481.2 , 5480.5)** | **42.1 (37.2 , 47.4)** |
| **Angola** | **173920 (155375 , 194999)** | **3633.4 (3271.7 , 4019.5)** | **698842 (623583 , 779687)** | **4890.1 (4406.6 , 5410.6)** | **34.6 (27.9 , 41.8)** |
| **Central African Republic** | **51619 (46253 , 57716)** | **3692.7 (3327.8 , 4109.1)** | **151456 (135850 , 168070)** | **5412.4 (4895.8 , 5972.7)** | **46.6 (39.7 , 53.1)** |
| **Congo** | **48701 (43588 , 54197)** | **3914.9 (3528.4 , 4349.4)** | **174060 (156556 , 194713)** | **5394.7 (4873.4 , 5983.7)** | **37.8 (30.4 , 45.2)** |
| **Democratic Republic of the Congo** | **617536 (553091 , 688720)** | **3389.3 (3046 , 3754.7)** | **2159011 (1921621 , 2410202)** | **4879.1 (4391.2 , 5419.3)** | **44 (37.2 , 51.3)** |
| **Equatorial Guinea** | **8085 (7242 , 9032)** | **3615.7 (3250.6 , 4039.5)** | **33892 (30333 , 37722)** | **5331 (4804.1 , 5911.4)** | **47.4 (39.5 , 55.7)** |
| **Gabon** | **24009 (21507 , 26714)** | **3987.1 (3577.8 , 4419.6)** | **72040 (64566 , 80284)** | **5959.7 (5380.6 , 6603.2)** | **49.5 (42.4 , 57.6)** |
