# Supplementary material for: Prevalence, Deaths and Disability-Adjusted-Life-Years (DALYs) Due to Type 2 Diabetes and Its Attributable Risk Factors in 204 Countries and Territories, 1990-2019: Results From the Global Burden of Disease Study 2019
Source: Front Endocrinol (Lausanne). 2022 Feb 25;13:838027. doi: 10.3389/fendo.2022.838027 (PMC8915203; doi:10.3389/fendo.2022.838027)
Supplement: Supplementary Table 1 — Guidelines for accurate and transparent health estimates reporting (GATHER) checklist. [file DataSheet_1.zip › Supplementary Tables/Supplementary Table S5.DOC]

| **Table S5: Deaths due to type 2 diabetes in 1990 and 2019 and the percentage change in age-standardised rates (ASRs) per 100,000 by location**  **(Generated from data available from http://ghdx.healthdata.org/gbd-results-tool)** | | | | | |
| --- | --- | --- | --- | --- | --- |
|  | **1990** | | **2019** | | **Percentage change in ASRs per 100,000** |
|  | **No (95% UI)** | **ASRs per 100,000 (95% UI)** | **No (95% UI)** | **ASRs per 100,000 (95% UI)** |  |
| **Global** | **606407 (573069 , 637508)** | **16.7 (15.7 , 17.5)** | **1472934 (1371940 , 1565860)** | **18.5 (17.2 , 19.7)** | **10.8 (4.4 , 17.4)** |
| **High-income North America** | **47800 (44590 , 49516)** | **13.2 (12.4 , 13.7)** | **80273 (73844 , 84054)** | **12.3 (11.4 , 12.9)** | **-7.2 (-10.3 , -4.2)** |
| **Canada** | **3875 (3546 , 4112)** | **12.1 (11 , 12.8)** | **6857 (5962 , 7504)** | **9.1 (8 , 9.9)** | **-24.6 (-29.9 , -18.9)** |
| **Greenland** | **5 (4 , 5)** | **15.6 (13.5 , 18)** | **6 (4 , 7)** | **9 (7.2 , 10.8)** | **-42.5 (-55.9 , -27.8)** |
| **United States of America** | **43919 (40926 , 45553)** | **13.4 (12.5 , 13.9)** | **73409 (67731 , 76759)** | **12.7 (11.8 , 13.2)** | **-5.5 (-8.7 , -2.3)** |
| **Australasia** | **2496 (2336 , 2613)** | **10.9 (10.1 , 11.4)** | **4778 (4217 , 5197)** | **8.7 (7.8 , 9.5)** | **-19.8 (-25.3 , -14.2)** |
| **Australia** | **2098 (1954 , 2203)** | **11 (10.2 , 11.6)** | **4122 (3618 , 4512)** | **8.9 (7.9 , 9.7)** | **-19.5 (-25.5 , -13.3)** |
| **New Zealand** | **398 (371 , 427)** | **10.3 (9.6 , 11)** | **656 (589 , 708)** | **7.9 (7.1 , 8.5)** | **-22.9 (-29.3 , -16.1)** |
| **High-income Asia Pacific** | **14975 (14172 , 15702)** | **7.8 (7.3 , 8.2)** | **21489 (18539 , 23637)** | **4.2 (3.7 , 4.6)** | **-45.8 (-51.6 , -42)** |
| **Brunei Darussalam** | **84 (73 , 100)** | **109.9 (95.1 , 128.4)** | **163 (144 , 184)** | **72.7 (64.7 , 81.9)** | **-33.9 (-44.8 , -19.6)** |
| **Japan** | **9868 (9202 , 10239)** | **6.1 (5.6 , 6.3)** | **8539 (6915 , 9451)** | **2 (1.7 , 2.1)** | **-67.6 (-70.3 , -65.9)** |
| **Singapore** | **362 (342 , 380)** | **18.2 (17 , 19.2)** | **175 (153 , 193)** | **2.4 (2.1 , 2.6)** | **-86.9 (-88.3 , -85.5)** |
| **Republic of Korea** | **4661 (4404 , 5760)** | **17 (15.9 , 23.7)** | **12612 (10966 , 14250)** | **14.7 (12.7 , 16.6)** | **-13.5 (-42.6 , -1.4)** |
| **Western Europe** | **78563 (73396 , 81364)** | **13.1 (12.1 , 13.6)** | **93438 (82484 , 99813)** | **8.5 (7.6 , 9.1)** | **-34.6 (-37.8 , -32)** |
| **Andorra** | **3 (2 , 4)** | **7.3 (5.5 , 9.7)** | **8 (7 , 11)** | **5.5 (4.3 , 7)** | **-23.5 (-46.4 , 6.1)** |
| **Austria** | **1522 (1415 , 1601)** | **12.2 (11.3 , 12.8)** | **2284 (2012 , 2486)** | **11 (9.8 , 11.9)** | **-9.7 (-16.5 , -2.7)** |
| **Belgium** | **1736 (1593 , 1841)** | **11 (10.1 , 11.7)** | **1662 (1447 , 1817)** | **6.1 (5.4 , 6.6)** | **-44.7 (-48.8 , -40.5)** |
| **Cyprus** | **426 (330 , 499)** | **64 (49.7 , 74.6)** | **479 (398 , 555)** | **26.9 (21.9 , 31)** | **-58 (-65.3 , -48.6)** |
| **Denmark** | **772 (723 , 822)** | **9.3 (8.7 , 9.9)** | **1351 (1207 , 1474)** | **10.8 (9.8 , 11.8)** | **16.9 (6.5 , 27)** |
| **Finland** | **460 (422 , 502)** | **6.4 (5.9 , 7)** | **446 (390 , 491)** | **3.3 (3 , 3.7)** | **-48.1 (-53.8 , -42.6)** |
| **France** | **6841 (6218 , 7390)** | **7.8 (7.1 , 8.3)** | **12220 (10361 , 13537)** | **7.1 (6.2 , 7.8)** | **-8.1 (-15.9 , 0)** |
| **Germany** | **20468 (18930 , 21677)** | **15.4 (14.2 , 16.3)** | **21707 (19168 , 23699)** | **9.7 (8.6 , 10.5)** | **-37.2 (-41.7 , -32.7)** |
| **Greece** | **1134 (1062 , 1192)** | **7.5 (7 , 7.9)** | **1350 (1204 , 1465)** | **5 (4.5 , 5.4)** | **-33.3 (-38.5 , -27.9)** |
| **Iceland** | **14 (13 , 16)** | **4.8 (4.3 , 5.2)** | **24 (21 , 28)** | **3.9 (3.4 , 4.4)** | **-17.7 (-28.2 , -5.6)** |
| **Ireland** | **403 (379 , 426)** | **9.9 (9.3 , 10.5)** | **480 (418 , 527)** | **6.1 (5.3 , 6.7)** | **-38.5 (-44.5 , -32.5)** |
| **Israel** | **796 (744 , 839)** | **17.1 (15.8 , 18)** | **2593 (2272 , 2812)** | **20.6 (18.2 , 22.3)** | **20.7 (11 , 29.7)** |
| **Italy** | **17472 (16492 , 18095)** | **19 (17.8 , 19.7)** | **21119 (18048 , 22728)** | **11.9 (10.4 , 12.7)** | **-37.3 (-42.1 , -33.9)** |
| **Luxembourg** | **55 (50 , 59)** | **10.2 (9.3 , 11)** | **64 (54 , 75)** | **5.8 (4.9 , 6.7)** | **-43.1 (-50.6 , -34.4)** |
| **Malta** | **94 (86 , 101)** | **23.1 (21.2 , 24.9)** | **119 (102 , 135)** | **11.8 (10.1 , 13.4)** | **-49 (-55.3 , -41.8)** |
| **Monaco** | **2 (2 , 3)** | **3 (2.4 , 3.7)** | **4 (3 , 4)** | **3.1 (2.5 , 3.7)** | **2.1 (-20.7 , 32)** |
| **Netherlands** | **3357 (3062 , 3568)** | **16.6 (15.1 , 17.6)** | **3192 (2833 , 3495)** | **8.5 (7.6 , 9.3)** | **-48.6 (-52.4 , -44.2)** |
| **Norway** | **493 (447 , 520)** | **6.7 (6.2 , 7.1)** | **622 (545 , 669)** | **5.6 (5 , 6)** | **-16.3 (-21.2 , -11.4)** |
| **Portugal** | **2778 (2635 , 2911)** | **19.9 (18.7 , 20.9)** | **4194 (3617 , 4599)** | **14.5 (12.7 , 15.8)** | **-27.3 (-33.8 , -21.1)** |
| **San Marino** | **2 (2 , 3)** | **7 (5.9 , 8.3)** | **5 (3 , 6)** | **5.8 (3.9 , 8)** | **-17.9 (-46.2 , 16.9)** |
| **Spain** | **9407 (8591 , 9963)** | **17.2 (15.6 , 18.2)** | **10011 (8469 , 11102)** | **8 (6.9 , 8.7)** | **-53.5 (-57.3 , -49.4)** |
| **Sweden** | **1355 (1223 , 1457)** | **8.4 (7.6 , 9)** | **1982 (1738 , 2154)** | **7.9 (7 , 8.6)** | **-5.8 (-13.2 , 1.8)** |
| **Switzerland** | **1509 (1374 , 1604)** | **13.4 (12.2 , 14.3)** | **1370 (1157 , 1510)** | **6.4 (5.5 , 7)** | **-52.2 (-56.2 , -48.1)** |
| **United Kingdom** | **7397 (6914 , 7654)** | **7.9 (7.4 , 8.2)** | **6069 (5424 , 6434)** | **4.3 (3.9 , 4.5)** | **-45.8 (-48.1 , -43.6)** |
| **Southern Latin America** | **8678 (8253 , 9008)** | **19.6 (18.5 , 20.3)** | **14810 (13695 , 15721)** | **17.4 (16.1 , 18.5)** | **-11.2 (-16.2 , -5.9)** |
| **Argentina** | **6574 (6224 , 6869)** | **21.1 (19.9 , 22.1)** | **10218 (9426 , 10971)** | **18.6 (17.2 , 19.9)** | **-12.1 (-18 , -5.5)** |
| **Chile** | **1441 (1366 , 1503)** | **15.4 (14.6 , 16.1)** | **3725 (3346 , 4031)** | **15.5 (13.9 , 16.8)** | **0.6 (-7.4 , 8.7)** |
| **Uruguay** | **663 (623 , 697)** | **16.8 (15.7 , 17.7)** | **866 (780 , 939)** | **14.6 (13.4 , 15.8)** | **-12.7 (-19.5 , -5.8)** |
| **Eastern Europe** | **11319 (10772 , 11702)** | **4.1 (3.9 , 4.3)** | **21555 (19017 , 24068)** | **6.1 (5.4 , 6.8)** | **48.3 (32.3 , 65.5)** |
| **Belarus** | **542 (509 , 569)** | **4.2 (4 , 4.4)** | **357 (289 , 452)** | **2.2 (1.8 , 2.8)** | **-47.5 (-58.2 , -34.1)** |
| **Estonia** | **67 (61 , 76)** | **3.3 (3 , 3.8)** | **123 (98 , 153)** | **4.5 (3.5 , 5.6)** | **34.9 (3.7 , 69.6)** |
| **Latvia** | **164 (153 , 176)** | **4.6 (4.3 , 5)** | **322 (270 , 386)** | **7.7 (6.4 , 9.2)** | **66.9 (38.3 , 100.9)** |
| **Lithuania** | **158 (148 , 172)** | **3.5 (3.3 , 3.8)** | **218 (177 , 264)** | **3.7 (3 , 4.5)** | **5.3 (-14.7 , 28.2)** |
| **Republic of Moldova** | **290 (275 , 305)** | **6.6 (6.2 , 6.9)** | **270 (232 , 310)** | **4.6 (4 , 5.3)** | **-29.8 (-39.9 , -19)** |
| **Russian Federation** | **7485 (7066 , 7755)** | **4.2 (4 , 4.4)** | **17838 (15440 , 20296)** | **7.4 (6.4 , 8.5)** | **75.6 (53.2 , 99.8)** |
| **Ukraine** | **2614 (2488 , 2731)** | **3.7 (3.5 , 3.9)** | **2427 (2073 , 2830)** | **3.2 (2.8 , 3.8)** | **-12.4 (-25.4 , 2.7)** |
| **Central Europe** | **17033 (16317 , 17694)** | **11.7 (11.2 , 12.2)** | **26839 (23348 , 30585)** | **11.9 (10.4 , 13.6)** | **1.7 (-10.7 , 14.7)** |
| **Albania** | **77 (69 , 85)** | **4.2 (3.7 , 4.6)** | **149 (111 , 199)** | **3.4 (2.6 , 4.6)** | **-17.4 (-38 , 9.7)** |
| **Bosnia and Herzegovina** | **501 (462 , 547)** | **14.3 (13.1 , 15.6)** | **2209 (1680 , 2725)** | **37 (28.3 , 45.6)** | **159.6 (81.3 , 222.7)** |
| **Bulgaria** | **1887 (1747 , 2010)** | **15.1 (14 , 16.1)** | **2102 (1698 , 2579)** | **13.8 (11.1 , 16.9)** | **-8.9 (-26.2 , 12)** |
| **Croatia** | **717 (666 , 771)** | **11.9 (11 , 12.7)** | **1084 (868 , 1319)** | **11.3 (9.1 , 13.8)** | **-5 (-24.1 , 16.2)** |
| **Czechia** | **1670 (1546 , 1755)** | **12.1 (11.2 , 12.7)** | **3229 (2630 , 3864)** | **14.4 (11.7 , 17.2)** | **18.9 (-1.4 , 41.7)** |
| **Hungary** | **1709 (1615 , 1789)** | **11.6 (10.9 , 12.1)** | **2392 (1985 , 2876)** | **11.7 (9.7 , 14.1)** | **0.8 (-16.5 , 20.6)** |
| **Montenegro** | **69 (59 , 78)** | **11.7 (10.1 , 13.1)** | **134 (112 , 161)** | **13.7 (11.5 , 16.4)** | **17.8 (-3.9 , 47.2)** |
| **North Macedonia** | **330 (293 , 364)** | **19.4 (17.2 , 21.3)** | **1001 (799 , 1230)** | **33.6 (27 , 40.9)** | **73.3 (34.6 , 117.2)** |
| **Poland** | **5317 (5060 , 5582)** | **12.1 (11.5 , 12.7)** | **7608 (6426 , 8901)** | **10.4 (8.8 , 12.2)** | **-13.9 (-26.2 , 0)** |
| **Romania** | **1810 (1723 , 1910)** | **6.5 (6.2 , 6.9)** | **2303 (1897 , 2712)** | **5.9 (4.9 , 7)** | **-8.7 (-24.3 , 7.6)** |
| **Serbia** | **1943 (1602 , 2201)** | **18.8 (15.6 , 21.3)** | **3514 (2871 , 4271)** | **21.4 (17.6 , 25.9)** | **13.8 (-11.6 , 45)** |
| **Slovakia** | **757 (694 , 826)** | **12.6 (11.6 , 13.7)** | **767 (603 , 955)** | **8.2 (6.5 , 10.2)** | **-35 (-49.9 , -17)** |
| **Slovenia** | **247 (195 , 308)** | **10.5 (8.3 , 13)** | **346 (270 , 435)** | **7.1 (5.6 , 8.9)** | **-32.6 (-51.5 , -9.2)** |
| **Central Asia** | **4117 (3931 , 4353)** | **8.9 (8.5 , 9.4)** | **16563 (14874 , 18516)** | **23.6 (21.4 , 26.1)** | **163.8 (138.1 , 192.6)** |
| **Armenia** | **451 (425 , 479)** | **17.6 (16.6 , 18.8)** | **1182 (987 , 1379)** | **28.6 (23.9 , 33.2)** | **62.3 (36.5 , 90.2)** |
| **Azerbaijan** | **531 (486 , 587)** | **10.7 (9.8 , 11.9)** | **1953 (1599 , 2362)** | **23 (18.9 , 27.5)** | **113.8 (67 , 161.4)** |
| **Georgia** | **595 (543 , 654)** | **9.7 (8.9 , 10.6)** | **1254 (1056 , 1452)** | **20.5 (17.2 , 23.8)** | **111.2 (75.6 , 149.4)** |
| **Kazakhstan** | **824 (775 , 875)** | **6.8 (6.4 , 7.2)** | **2437 (2122 , 2787)** | **15.4 (13.5 , 17.6)** | **128.6 (96.6 , 163.7)** |
| **Kyrgyzstan** | **183 (170 , 196)** | **6.1 (5.6 , 6.5)** | **294 (256 , 336)** | **6.6 (5.7 , 7.5)** | **8.1 (-7.5 , 25)** |
| **Mongolia** | **45 (36 , 56)** | **4.4 (3.6 , 5.4)** | **113 (86 , 151)** | **5 (3.9 , 6.5)** | **14.2 (-13.3 , 50)** |
| **Tajikistan** | **327 (299 , 362)** | **11.6 (10.6 , 12.8)** | **1478 (1182 , 1846)** | **31.3 (25.2 , 38.1)** | **169 (108.6 , 232.8)** |
| **Turkmenistan** | **187 (173 , 199)** | **9.7 (9 , 10.3)** | **660 (522 , 834)** | **16.4 (13.1 , 20.6)** | **68.5 (32.9 , 110.8)** |
| **Uzbekistan** | **974 (894 , 1056)** | **8.6 (7.9 , 9.3)** | **7191 (5989 , 8488)** | **35.3 (30 , 41.2)** | **310.4 (244.4 , 381.3)** |
| **Central Latin America** | **34028 (32563 , 34915)** | **43.4 (41.2 , 44.7)** | **102719 (90492 , 115594)** | **44.6 (39.3 , 50.2)** | **2.7 (-9.1 , 15)** |
| **Colombia** | **3194 (3014 , 3335)** | **19.8 (18.4 , 20.8)** | **6892 (5446 , 8681)** | **12.8 (10.1 , 16.2)** | **-35.4 (-49.2 , -19)** |
| **Costa Rica** | **255 (236 , 272)** | **15.2 (14 , 16.2)** | **563 (441 , 710)** | **11.1 (8.6 , 13.9)** | **-27.1 (-43.3 , -8.4)** |
| **El Salvador** | **497 (456 , 561)** | **17.2 (15.7 , 19.6)** | **2182 (1666 , 2794)** | **36.3 (27.7 , 46.7)** | **111 (52.3 , 174.2)** |
| **Guatemala** | **544 (481 , 606)** | **16.6 (14.7 , 18.3)** | **6347 (5059 , 7861)** | **60.1 (48.4 , 74)** | **262.9 (185.3 , 360.8)** |
| **Honduras** | **246 (201 , 283)** | **11.9 (9.5 , 13.8)** | **1067 (863 , 1338)** | **18.7 (15.3 , 22.9)** | **56.6 (26.7 , 96.5)** |
| **Mexico** | **25670 (24585 , 26412)** | **63.9 (60.7 , 65.9)** | **72036 (62171 , 81851)** | **63.9 (55.4 , 72.6)** | **0.1 (-12 , 13.3)** |
| **Nicaragua** | **409 (375 , 449)** | **28.5 (26 , 31.6)** | **1868 (1564 , 2202)** | **46.8 (39.7 , 54.4)** | **64.6 (38 , 92.1)** |
| **Panama** | **261 (238 , 280)** | **18.4 (16.7 , 19.7)** | **1180 (927 , 1472)** | **28.2 (22.1 , 35.3)** | **53.5 (20.6 , 93.5)** |
| **Venezuela (Bolivarian Republic of)** | **2953 (2792 , 3097)** | **32.7 (30.7 , 34.4)** | **10585 (8192 , 13562)** | **37.8 (29.4 , 48.3)** | **15.6 (-10.5 , 46.2)** |
| **Andean Latin America** | **3739 (3402 , 4179)** | **19.4 (17.6 , 21.6)** | **13083 (10913 , 15442)** | **24 (20.1 , 28.3)** | **24 (1.8 , 48.1)** |
| **Bolivia (Plurinational State of)** | **1163 (933 , 1456)** | **39.2 (31.5 , 48.4)** | **3668 (2857 , 4659)** | **45.1 (35.5 , 56.8)** | **15.2 (-9.4 , 49.3)** |
| **Ecuador** | **1082 (1018 , 1174)** | **21.8 (20.4 , 23.9)** | **4830 (3861 , 6087)** | **34.5 (27.8 , 43.2)** | **58.1 (23.7 , 97.7)** |
| **Peru** | **1494 (1295 , 1729)** | **13.3 (11.5 , 15.3)** | **4585 (3449 , 6033)** | **14.4 (10.8 , 18.9)** | **8.5 (-21.7 , 48)** |
| **Caribbean** | **10256 (9540 , 10943)** | **41 (38.1 , 43.7)** | **18404 (15492 , 21815)** | **35.5 (29.9 , 42.1)** | **-13.3 (-26.1 , 1.8)** |
| **Antigua and Barbuda** | **31 (29 , 34)** | **56.9 (51.8 , 61.5)** | **54 (47 , 62)** | **58.2 (50.5 , 66.6)** | **2.3 (-12 , 18.6)** |
| **Barbados** | **211 (194 , 224)** | **68.4 (63.2 , 72.6)** | **300 (251 , 348)** | **60.7 (50.9 , 70.6)** | **-11.2 (-25 , 3.6)** |
| **Belize** | **39 (35 , 42)** | **43.1 (39 , 46.8)** | **146 (127 , 166)** | **56.3 (48.9 , 64)** | **30.6 (13.3 , 51.1)** |
| **Bermuda** | **18 (17 , 20)** | **31.3 (28.7 , 33.9)** | **23 (19 , 28)** | **17 (14.2 , 20.6)** | **-45.7 (-54.2 , -33.8)** |
| **Bahamas** | **69 (63 , 76)** | **48.5 (44 , 52.9)** | **137 (114 , 166)** | **38 (31.8 , 45.6)** | **-21.5 (-35.9 , -4.2)** |
| **Cuba** | **2143 (2014 , 2246)** | **21.2 (19.9 , 22.2)** | **1968 (1601 , 2383)** | **10 (8.2 , 12.2)** | **-52.7 (-61 , -43)** |
| **Dominica** | **49 (44 , 54)** | **65.8 (59 , 73)** | **55 (46 , 66)** | **60.7 (50.4 , 72.4)** | **-7.8 (-25.7 , 13)** |
| **Dominican Republic** | **736 (653 , 838)** | **21.2 (18.7 , 24)** | **2757 (2112 , 3593)** | **30.5 (23.5 , 39.3)** | **44 (9.2 , 88.2)** |
| **Grenada** | **51 (47 , 56)** | **67.7 (61.4 , 74.2)** | **73 (66 , 80)** | **71.4 (64.6 , 78.1)** | **5.5 (-6.5 , 18.2)** |
| **Guyana** | **285 (251 , 317)** | **79.8 (70.8 , 88.7)** | **539 (424 , 673)** | **92.8 (74.5 , 114.7)** | **16.3 (-9.3 , 45.8)** |
| **Haiti** | **2359 (1848 , 2927)** | **78.5 (61.6 , 97.3)** | **3827 (2678 , 5441)** | **61.6 (43.8 , 87)** | **-21.5 (-42.1 , 6.3)** |
| **Jamaica** | **1105 (1029 , 1169)** | **61 (56.9 , 64.4)** | **2342 (1925 , 2835)** | **75.5 (61.8 , 91.9)** | **23.9 (1.2 , 51.6)** |
| **Puerto Rico** | **1570 (1455 , 1652)** | **44.4 (40.9 , 46.7)** | **3177 (2485 , 3971)** | **40.7 (32 , 51.2)** | **-8.2 (-27.1 , 15.4)** |
| **Saint Kitts and Nevis** | **26 (23 , 28)** | **69.6 (63.8 , 75.5)** | **31 (27 , 36)** | **53.9 (46.8 , 62.3)** | **-22.6 (-33.8 , -8.6)** |
| **Saint Lucia** | **69 (64 , 74)** | **83.9 (77.5 , 89.4)** | **126 (107 , 147)** | **60.9 (51.7 , 71.3)** | **-27.4 (-39.1 , -14.6)** |
| **Saint Vincent and the Grenadines** | **59 (54 , 64)** | **85.8 (78.3 , 92.6)** | **103 (91 , 118)** | **80.4 (71.1 , 91.6)** | **-6.3 (-18.9 , 6.2)** |
| **Suriname** | **90 (81 , 97)** | **36 (32.8 , 39.1)** | **258 (216 , 307)** | **44.5 (37.4 , 52.8)** | **23.4 (2.8 , 49.2)** |
| **Trinidad and Tobago** | **974 (930 , 1021)** | **121.6 (115.7 , 127.4)** | **1790 (1373 , 2286)** | **97.8 (75.3 , 124.1)** | **-19.6 (-38.5 , 2)** |
| **United States Virgin Islands** | **30 (26 , 35)** | **40.7 (34.8 , 46.6)** | **76 (66 , 88)** | **42.6 (36.7 , 48.6)** | **4.6 (-13.4 , 28.1)** |
| **Tropical Latin America** | **26027 (24694 , 27093)** | **31.8 (29.8 , 33.3)** | **65273 (59189 , 68859)** | **28 (25.3 , 29.6)** | **-12 (-16.5 , -7.9)** |
| **Brazil** | **25556 (24248 , 26642)** | **32.1 (30 , 33.6)** | **62883 (57009 , 66255)** | **27.6 (25 , 29.2)** | **-13.9 (-18.5 , -9.7)** |
| **Paraguay** | **472 (414 , 529)** | **22.8 (19.9 , 25.6)** | **2390 (1853 , 3009)** | **45.4 (35.2 , 57.3)** | **98.7 (52.1 , 158)** |
| **East Asia** | **70497 (62737 , 79640)** | **9.1 (8.2 , 10.3)** | **182874 (157802 , 208399)** | **9.6 (8.3 , 11)** | **5.4 (-12.2 , 23.7)** |
| **China** | **64084 (56365 , 73219)** | **8.6 (7.7 , 9.8)** | **168388 (143233 , 194030)** | **9.2 (7.9 , 10.5)** | **6.3 (-13.7 , 26)** |
| **Democratic People's Republic of Korea** | **1930 (1515 , 2448)** | **13.4 (10.6 , 16.9)** | **3855 (3143 , 4725)** | **12.4 (10.1 , 15.2)** | **-7.4 (-29.3 , 20.8)** |
| **Taiwan (Province of China)** | **4482 (4249 , 4669)** | **31.4 (29.5 , 32.8)** | **10631 (8440 , 13386)** | **26.5 (21.1 , 33.5)** | **-15.5 (-32.1 , 6.2)** |
| **Southeast Asia** | **75561 (67937 , 83121)** | **32.4 (29.1 , 35.4)** | **214764 (193046 , 236296)** | **38.1 (34.1 , 41.7)** | **17.3 (3.3 , 32.6)** |
| **Cambodia** | **1285 (998 , 1580)** | **31.4 (25 , 37.9)** | **3352 (2597 , 4088)** | **31.8 (24.8 , 38.5)** | **1.3 (-21.4 , 24.9)** |
| **Indonesia** | **29125 (25556 , 32931)** | **31 (27.4 , 34.9)** | **98679 (82291 , 113404)** | **48.3 (40.5 , 54.3)** | **55.6 (28.3 , 83.9)** |
| **Lao People's Democratic Republic** | **729 (543 , 974)** | **38 (28.9 , 49)** | **1439 (1122 , 1826)** | **37.5 (29.4 , 47.2)** | **-1.1 (-27.9 , 41.7)** |
| **Malaysia** | **2238 (2015 , 2458)** | **26.6 (23.8 , 29.2)** | **3233 (2549 , 4249)** | **13.3 (10.5 , 17.5)** | **-50 (-60.8 , -30.4)** |
| **Maldives** | **24 (19 , 32)** | **32.4 (26.5 , 40.4)** | **48 (40 , 58)** | **19 (15.6 , 22.6)** | **-41.5 (-55.8 , -21.9)** |
| **Mauritius** | **318 (298 , 337)** | **44.7 (42 , 47.4)** | **1819 (1501 , 2202)** | **107.3 (88.8 , 129.4)** | **140.1 (95.9 , 189.6)** |
| **Myanmar** | **11490 (8680 , 14815)** | **53.1 (41.3 , 66.6)** | **20862 (17962 , 24265)** | **50.1 (43.6 , 57.7)** | **-5.7 (-27.5 , 25.5)** |
| **Philippines** | **8529 (7704 , 9403)** | **35.5 (31.9 , 39.1)** | **26039 (21645 , 31289)** | **37 (31.1 , 43.8)** | **4 (-14 , 27)** |
| **Sri Lanka** | **2248 (2013 , 2510)** | **25.1 (22.3 , 27.8)** | **12740 (9763 , 16396)** | **57.1 (43 , 73.1)** | **127.6 (65.7 , 197.5)** |
| **Seychelles** | **8 (7 , 9)** | **14.1 (12.5 , 15.9)** | **24 (21 , 28)** | **24.4 (21.2 , 27.7)** | **72.9 (45.1 , 103.1)** |
| **Thailand** | **8086 (6937 , 9388)** | **24.9 (21.3 , 29)** | **18338 (13632 , 23776)** | **18.2 (13.6 , 23.6)** | **-26.7 (-47.3 , 1.9)** |
| **Timor-Leste** | **53 (41 , 69)** | **21.9 (17.1 , 28)** | **176 (135 , 223)** | **24.4 (18.9 , 30.6)** | **11.6 (-16.5 , 44.7)** |
| **Viet Nam** | **11327 (9050 , 13677)** | **30.9 (24.6 , 37.2)** | **27733 (22236 , 33469)** | **34.5 (27.8 , 41.5)** | **11.6 (-13.7 , 47.9)** |
| **Oceania** | **2372 (1965 , 2912)** | **87.7 (73.8 , 107.5)** | **7766 (6264 , 9578)** | **121 (100.2 , 146.5)** | **38 (8.3 , 68.6)** |
| **American Samoa** | **16 (14 , 18)** | **80.5 (71.3 , 94.3)** | **43 (37 , 50)** | **98.1 (84.7 , 113.6)** | **21.8 (-0.5 , 46)** |
| **Cook Islands** | **14 (12 , 17)** | **121.4 (105.6 , 141.6)** | **27 (23 , 32)** | **110.4 (92.8 , 130.4)** | **-9 (-26.7 , 12.6)** |
| **Micronesia (Federated States of)** | **41 (32 , 51)** | **94.5 (75.1 , 117.1)** | **107 (77 , 151)** | **169.1 (126.8 , 226.1)** | **79 (21.1 , 144.9)** |
| **Fiji** | **508 (413 , 652)** | **158.1 (129.6 , 203.4)** | **1698 (1359 , 2086)** | **257.4 (210.3 , 309.2)** | **62.8 (20.4 , 118)** |
| **Guam** | **24 (21 , 27)** | **39.8 (33.6 , 45.5)** | **45 (38 , 54)** | **23.7 (19.9 , 28.6)** | **-40.5 (-52.4 , -20.7)** |
| **Kiribati** | **53 (43 , 65)** | **146.4 (120.1 , 175.8)** | **134 (101 , 170)** | **204 (158.3 , 252.7)** | **39.3 (1.5 , 82.5)** |
| **Marshall Islands** | **12 (10 , 15)** | **82.8 (68.8 , 99.4)** | **35 (26 , 46)** | **112.9 (86.9 , 145.9)** | **36.3 (2.3 , 75.1)** |
| **Nauru** | **4 (3 , 5)** | **112.8 (90 , 144.8)** | **6 (4 , 9)** | **162.8 (126.9 , 227)** | **44.3 (13.7 , 79.3)** |
| **Niue** | **2 (2 , 2)** | **88.3 (72.1 , 110.6)** | **3 (2 , 3)** | **122 (95.2 , 162.5)** | **38.1 (6.2 , 73)** |
| **Northern Mariana Islands** | **7 (6 , 9)** | **50.4 (43.1 , 60.4)** | **27 (22 , 31)** | **58.5 (50 , 67.7)** | **16 (-4.8 , 38.5)** |
| **Palau** | **7 (6 , 9)** | **79.1 (64 , 98.4)** | **23 (17 , 29)** | **119.2 (92.2 , 148.2)** | **50.8 (9 , 101.7)** |
| **Papua New Guinea** | **1310 (1020 , 1659)** | **77.1 (61 , 97.5)** | **4499 (3465 , 5726)** | **104 (82.5 , 130.1)** | **34.9 (3 , 71.5)** |
| **Samoa** | **58 (46 , 73)** | **71.7 (58 , 90.1)** | **121 (98 , 153)** | **89.2 (72.7 , 111.3)** | **24.4 (-4.2 , 61)** |
| **Solomon Islands** | **108 (78 , 151)** | **76.7 (56.5 , 108.4)** | **417 (336 , 513)** | **133.6 (110.6 , 162.8)** | **74.3 (17.5 , 145)** |
| **Tokelau** | **1 (1 , 1)** | **75.6 (60.6 , 92.8)** | **1 (1 , 2)** | **96.5 (75 , 123.2)** | **27.6 (-5.6 , 65.5)** |
| **Tonga** | **44 (37 , 52)** | **86.3 (72.2 , 100.9)** | **81 (64 , 100)** | **104.6 (83 , 129.1)** | **21.3 (-5 , 54.9)** |
| **Tuvalu** | **6 (5 , 7)** | **88.5 (72.6 , 107.6)** | **11 (8 , 15)** | **115.8 (88.4 , 155.1)** | **30.9 (-4.9 , 79.7)** |
| **Vanuatu** | **26 (19 , 37)** | **43.8 (32.1 , 62.1)** | **123 (92 , 165)** | **77.5 (58.3 , 103.1)** | **77.2 (26.1 , 145.8)** |
| **North Africa and Middle East** | **36365 (33087 , 40381)** | **24.8 (22.5 , 27.7)** | **95375 (84916 , 107421)** | **25.2 (22.4 , 28.2)** | **1.7 (-10.4 , 14.9)** |
| **Afghanistan** | **1855 (1206 , 2762)** | **27.8 (18.5 , 40.5)** | **4331 (2506 , 6392)** | **39.9 (23.4 , 58)** | **43.4 (3.7 , 85.1)** |
| **Algeria** | **1531 (1099 , 2117)** | **17 (12.6 , 23.5)** | **4995 (3849 , 6447)** | **18.2 (13.9 , 23.4)** | **6.9 (-19 , 41.8)** |
| **Bahrain** | **96 (81 , 113)** | **77.8 (66.2 , 91)** | **700 (554 , 878)** | **127 (102.5 , 154.6)** | **63.3 (23 , 103.2)** |
| **Egypt** | **6010 (5569 , 6529)** | **23.3 (21.4 , 25.4)** | **19233 (14457 , 25267)** | **33.7 (26 , 43.8)** | **44.8 (10.5 , 89.1)** |
| **Iran (Islamic Republic of)** | **2414 (2110 , 2761)** | **12.5 (10.7 , 14.4)** | **14191 (12014 , 15320)** | **22 (18.4 , 23.8)** | **76.4 (38.3 , 110.8)** |
| **Iraq** | **3584 (2884 , 4284)** | **49.2 (39.4 , 58.6)** | **9038 (7191 , 10968)** | **45.8 (37.1 , 54.4)** | **-7 (-28.6 , 19.8)** |
| **Jordan** | **666 (562 , 783)** | **63.9 (53.8 , 74.9)** | **2000 (1694 , 2396)** | **40.2 (34.1 , 47.5)** | **-37.1 (-50.1 , -21)** |
| **Kuwait** | **130 (117 , 145)** | **28.8 (25.4 , 32.2)** | **354 (295 , 423)** | **18.5 (15.3 , 22.2)** | **-35.8 (-45.5 , -24.7)** |
| **Lebanon** | **400 (345 , 481)** | **20.4 (17.5 , 24.4)** | **821 (588 , 1052)** | **15.9 (11.4 , 20.4)** | **-21.9 (-44.4 , 2.8)** |
| **Libya** | **238 (174 , 300)** | **14.2 (10.5 , 18)** | **834 (605 , 1106)** | **18.3 (13.3 , 24.1)** | **28.9 (-10 , 82.3)** |
| **Morocco** | **1562 (1254 , 2196)** | **13.1 (10.4 , 18.6)** | **6196 (4687 , 7779)** | **22.4 (17.2 , 28.3)** | **70.8 (31.5 , 112.6)** |
| **Palestine** | **413 (327 , 511)** | **53.9 (43 , 66.4)** | **1257 (1081 , 1443)** | **68.8 (59.5 , 78.7)** | **27.7 (0.8 , 65.3)** |
| **Oman** | **233 (179 , 301)** | **44.7 (34.2 , 57.2)** | **642 (547 , 743)** | **58.3 (50.4 , 66.5)** | **30.4 (-1.9 , 74)** |
| **Qatar** | **62 (52 , 74)** | **111.2 (94.4 , 130.9)** | **336 (253 , 438)** | **122.1 (98.9 , 151.5)** | **9.8 (-15.1 , 41.1)** |
| **Saudi Arabia** | **1268 (950 , 1683)** | **25.2 (19.2 , 33.1)** | **2797 (2211 , 3439)** | **19.5 (16.1 , 23.7)** | **-22.5 (-43.8 , 7)** |
| **Sudan** | **957 (693 , 1334)** | **11.5 (8.4 , 16.4)** | **2549 (1723 , 3552)** | **15.7 (10.6 , 21.7)** | **36 (-0.3 , 81.3)** |
| **Syrian Arab Republic** | **873 (683 , 1089)** | **18.7 (14.4 , 23.2)** | **1599 (1216 , 2139)** | **15.9 (12.3 , 20.8)** | **-14.9 (-36.6 , 20.4)** |
| **Tunisia** | **459 (371 , 616)** | **10.9 (8.7 , 15)** | **1722 (1237 , 2322)** | **14.8 (10.7 , 19.9)** | **36.1 (-0.8 , 85.4)** |
| **Turkey** | **12931 (11086 , 14894)** | **40.3 (34.6 , 47)** | **18831 (15145 , 22949)** | **22.7 (18.3 , 27.6)** | **-43.7 (-56.3 , -26.7)** |
| **United Arab Emirates** | **175 (137 , 217)** | **75.3 (58.3 , 92.7)** | **1232 (869 , 1662)** | **55.2 (41.4 , 70.7)** | **-26.7 (-51.7 , 3.3)** |
| **Yemen** | **485 (333 , 720)** | **11.7 (8.1 , 17.9)** | **1620 (1138 , 2302)** | **14.2 (10.2 , 19.8)** | **21.6 (-9.7 , 62.6)** |
| **South Asia** | **95155 (82522 , 110905)** | **22.1 (19.1 , 25.8)** | **337920 (300995 , 379757)** | **28.1 (25 , 31.6)** | **27.5 (8.2 , 48.9)** |
| **Bangladesh** | **10363 (8825 , 12113)** | **27 (22.9 , 31)** | **30639 (24633 , 37232)** | **29 (23.3 , 35.2)** | **7.6 (-15.2 , 37.8)** |
| **Bhutan** | **36 (26 , 48)** | **18.6 (13.8 , 24.2)** | **147 (112 , 189)** | **30.4 (23.4 , 38.7)** | **63.6 (22.8 , 121.1)** |
| **India** | **72125 (61629 , 85897)** | **21.1 (18.1 , 25.1)** | **258927 (224420 , 300779)** | **26.2 (22.8 , 30.3)** | **23.9 (3.1 , 49.2)** |
| **Nepal** | **802 (639 , 1005)** | **11.1 (8.8 , 13.7)** | **3245 (2563 , 3952)** | **18.7 (14.7 , 22.6)** | **68.3 (25.9 , 122.2)** |
| **Pakistan** | **11829 (7885 , 14518)** | **23.2 (15.4 , 28.4)** | **44961 (35203 , 55337)** | **49.6 (38.7 , 60.8)** | **113.7 (58 , 201.3)** |
| **Southern Sub-Saharan Africa** | **10626 (9436 , 11763)** | **42.6 (37.7 , 47.3)** | **33921 (31445 , 36569)** | **68.5 (63.2 , 73.8)** | **60.7 (44.1 , 77.7)** |
| **Botswana** | **297 (232 , 385)** | **59.5 (47.1 , 76.6)** | **1036 (768 , 1344)** | **90.8 (69.7 , 116.9)** | **52.5 (12.5 , 107.1)** |
| **Lesotho** | **449 (369 , 541)** | **50.2 (41.3 , 60)** | **1236 (903 , 1612)** | **110.9 (82.5 , 143.1)** | **120.7 (60.3 , 192.4)** |
| **Namibia** | **376 (313 , 438)** | **56.8 (47.8 , 66.3)** | **767 (605 , 967)** | **61.7 (49 , 77.7)** | **8.6 (-14.8 , 39.2)** |
| **South Africa** | **8086 (6925 , 9115)** | **42.1 (35.8 , 47.6)** | **26872 (24568 , 29032)** | **67.7 (62 , 73.1)** | **60.7 (43.5 , 78.9)** |
| **Eswatini** | **210 (165 , 256)** | **83.3 (65.6 , 100.9)** | **669 (482 , 897)** | **134.6 (97.5 , 177.3)** | **61.6 (19.4 , 120.7)** |
| **Zimbabwe** | **1209 (1058 , 1373)** | **35 (30.4 , 39.8)** | **3341 (2604 , 4127)** | **55.6 (43.9 , 68.7)** | **59.2 (22.9 , 100.6)** |
| **Western Sub-Saharan Africa** | **21970 (18985 , 25317)** | **29.5 (25.6 , 33.6)** | **55073 (47194 , 63292)** | **35.6 (30.8 , 40.2)** | **20.8 (3.4 , 40.1)** |
| **Benin** | **474 (403 , 558)** | **26.3 (22.4 , 30.8)** | **1416 (1113 , 1817)** | **34.4 (27.3 , 43.4)** | **30.7 (2.1 , 64.9)** |
| **Burkina Faso** | **1228 (1011 , 1482)** | **34.1 (28.2 , 40.3)** | **2683 (2190 , 3243)** | **34.8 (28.8 , 41.5)** | **2.2 (-16.5 , 25.9)** |
| **Cameroon** | **1324 (1028 , 1662)** | **35.4 (27.6 , 44.6)** | **4871 (3741 , 6407)** | **49.2 (38.3 , 62.7)** | **38.9 (2.1 , 86.9)** |
| **Cabo Verde** | **20 (17 , 28)** | **8.5 (7.2 , 11.6)** | **128 (108 , 150)** | **31.3 (26.3 , 36.5)** | **270.2 (109.5 , 369.6)** |
| **Chad** | **601 (472 , 737)** | **23.6 (18.7 , 28.8)** | **1579 (1230 , 2020)** | **33.1 (26.1 , 41.5)** | **40.2 (13.4 , 72.4)** |
| **CÃ´te d'Ivoire** | **1017 (831 , 1207)** | **32.1 (26.9 , 37.5)** | **3242 (2527 , 4062)** | **38.4 (30.8 , 47)** | **19.7 (-6.2 , 50.8)** |
| **Gambia** | **70 (53 , 88)** | **24.2 (19.1 , 30.1)** | **307 (231 , 389)** | **37 (28 , 46.2)** | **52.6 (12 , 104.2)** |
| **Ghana** | **1501 (1221 , 1830)** | **28.9 (24.1 , 34.6)** | **5856 (4787 , 7219)** | **42.7 (35.4 , 51.1)** | **47.9 (13.3 , 86.2)** |
| **Guinea** | **820 (666 , 987)** | **27.6 (22.3 , 33.2)** | **1850 (1443 , 2289)** | **37.7 (29.5 , 46.1)** | **36.5 (2.8 , 76.8)** |
| **Guinea-Bissau** | **146 (112 , 191)** | **40.2 (31.6 , 51.7)** | **295 (222 , 377)** | **48.6 (37.3 , 61)** | **20.8 (-7.8 , 60.8)** |
| **Liberia** | **287 (238 , 339)** | **29.3 (24.8 , 34.5)** | **587 (441 , 765)** | **34.6 (26.4 , 44.4)** | **17.9 (-11.7 , 56.6)** |
| **Mali** | **1008 (828 , 1214)** | **28.6 (23.4 , 34.5)** | **2528 (2024 , 3108)** | **34.6 (27.7 , 41.6)** | **21.1 (-5.4 , 52.3)** |
| **Mauritania** | **301 (250 , 363)** | **33.8 (28.4 , 40.2)** | **596 (458 , 762)** | **33.1 (25.8 , 41.6)** | **-2.1 (-23.5 , 23.7)** |
| **Niger** | **571 (445 , 704)** | **24.9 (19.6 , 30.6)** | **1926 (1486 , 2415)** | **30.7 (24.1 , 37.5)** | **23.2 (-0.9 , 54.1)** |
| **Nigeria** | **11125 (9094 , 13521)** | **29.8 (24.6 , 35.6)** | **22797 (18033 , 28412)** | **32.8 (26.6 , 40)** | **10.3 (-14 , 40.1)** |
| **Sao Tome and Principe** | **6 (5 , 8)** | **11.2 (9.4 , 13)** | **13 (11 , 15)** | **14.8 (12.2 , 17.3)** | **31.6 (4.9 , 66.5)** |
| **Senegal** | **793 (635 , 951)** | **28.5 (23.2 , 34.2)** | **2430 (1877 , 3047)** | **37.9 (29.9 , 46.8)** | **32.6 (3.9 , 70.2)** |
| **Sierra Leone** | **413 (335 , 499)** | **23.9 (19.6 , 28.8)** | **1006 (775 , 1291)** | **32.7 (25.6 , 41.1)** | **36.5 (5.9 , 76.7)** |
| **Togo** | **266 (217 , 322)** | **25.8 (21.3 , 31)** | **963 (728 , 1248)** | **32.8 (25.4 , 41.9)** | **27.4 (-1 , 61.8)** |
| **Eastern Sub-Saharan Africa** | **26753 (23411 , 30665)** | **41.2 (36.2 , 46.7)** | **49121 (43551 , 55699)** | **36.5 (32.5 , 40.9)** | **-11.4 (-25.6 , 1.2)** |
| **Burundi** | **946 (735 , 1251)** | **44.7 (35.3 , 58.6)** | **1329 (1032 , 1700)** | **35.7 (28.4 , 44.5)** | **-20.1 (-39.4 , 5.1)** |
| **Comoros** | **73 (47 , 92)** | **37.4 (26 , 46.4)** | **152 (126 , 189)** | **35.1 (29.4 , 43.4)** | **-6 (-27.1 , 37.8)** |
| **Djibouti** | **36 (27 , 48)** | **33.4 (26.1 , 43.2)** | **192 (148 , 258)** | **41.9 (33.9 , 53.5)** | **25.4 (-2 , 63.3)** |
| **Eritrea** | **320 (226 , 446)** | **37.3 (26.5 , 52.4)** | **958 (720 , 1277)** | **44.7 (34.3 , 58.3)** | **19.8 (-14.3 , 65.8)** |
| **Ethiopia** | **9797 (7971 , 12669)** | **55.1 (45.2 , 68.9)** | **11853 (10174 , 13797)** | **34.5 (29.6 , 39.9)** | **-37.5 (-54.9 , -19.8)** |
| **Kenya** | **1883 (1669 , 2166)** | **27.3 (24.2 , 31.2)** | **5738 (4776 , 6901)** | **31.9 (26.9 , 37.4)** | **17 (1.8 , 35.3)** |
| **Madagascar** | **1350 (1111 , 1609)** | **30.2 (24.9 , 36)** | **2601 (1975 , 3357)** | **30 (23.2 , 38.4)** | **-0.8 (-24.6 , 29)** |
| **Malawi** | **1287 (1092 , 1497)** | **40.1 (34.1 , 46.4)** | **2285 (1891 , 2735)** | **37.2 (31.3 , 43.7)** | **-7.3 (-24.5 , 13.8)** |
| **Mozambique** | **1823 (1510 , 2161)** | **36.6 (30.5 , 42.9)** | **4245 (3352 , 5361)** | **45.5 (36.7 , 56.7)** | **24.2 (-4 , 59.5)** |
| **Rwanda** | **1262 (987 , 1609)** | **49.4 (38.9 , 62.6)** | **1763 (1345 , 2303)** | **36.5 (28 , 47.3)** | **-26.1 (-43 , -5.5)** |
| **Somalia** | **925 (693 , 1215)** | **43.1 (33.3 , 55.3)** | **2308 (1743 , 2990)** | **42.7 (33.3 , 54.1)** | **-0.9 (-23.6 , 30.8)** |
| **South Sudan** | **724 (564 , 918)** | **35 (27.7 , 44.3)** | **1045 (765 , 1373)** | **33.7 (25.4 , 43.1)** | **-3.8 (-27.6 , 25.6)** |
| **United Republic of Tanzania** | **3217 (2735 , 3740)** | **35.1 (29.9 , 40.4)** | **7621 (6127 , 9170)** | **36.5 (29.9 , 43.8)** | **4 (-16.6 , 29.6)** |
| **Uganda** | **2072 (1597 , 2713)** | **37.7 (29.5 , 49)** | **4632 (3565 , 5897)** | **39.8 (31.1 , 50.3)** | **5.6 (-15.6 , 31.6)** |
| **Zambia** | **1019 (857 , 1193)** | **42.2 (35.8 , 49.3)** | **2361 (1907 , 2906)** | **42.6 (35.4 , 51.4)** | **1 (-20 , 27.7)** |
| **Central Sub-Saharan Africa** | **8077 (6915 , 9445)** | **43 (37.2 , 49.2)** | **16894 (13940 , 20678)** | **38.3 (32.1 , 46.2)** | **-10.9 (-27.6 , 8.9)** |
| **Angola** | **1372 (1062 , 1720)** | **41 (32.8 , 50)** | **3691 (2970 , 4610)** | **41.2 (34.3 , 50.4)** | **0.5 (-24.9 , 37.2)** |
| **Central African Republic** | **509 (413 , 617)** | **48.4 (39.5 , 57.8)** | **925 (693 , 1221)** | **48.8 (38.1 , 62.6)** | **0.9 (-23 , 32.7)** |
| **Congo** | **532 (437 , 636)** | **57.2 (47 , 68.5)** | **1094 (868 , 1376)** | **51.4 (42 , 62.5)** | **-10.1 (-30 , 16)** |
| **Democratic Republic of the Congo** | **5276 (4353 , 6396)** | **41.5 (35.1 , 48.9)** | **10386 (8251 , 12973)** | **34.8 (28.2 , 42.7)** | **-16.3 (-35.5 , 6.6)** |
| **Equatorial Guinea** | **78 (58 , 98)** | **43.4 (32.9 , 54.1)** | **210 (151 , 290)** | **54.3 (40.3 , 72.2)** | **25.2 (-13.2 , 81.3)** |
| **Gabon** | **310 (236 , 414)** | **60.8 (46.6 , 81)** | **588 (458 , 731)** | **65.2 (51.3 , 79.5)** | **7.3 (-22.3 , 42.2)** |
