# Supplementary material for: Prevalence, Deaths and Disability-Adjusted-Life-Years (DALYs) Due to Type 2 Diabetes and Its Attributable Risk Factors in 204 Countries and Territories, 1990-2019: Results From the Global Burden of Disease Study 2019
Source: Front Endocrinol (Lausanne). 2022 Feb 25;13:838027. doi: 10.3389/fendo.2022.838027 (PMC8915203; doi:10.3389/fendo.2022.838027)
Supplement: Supplementary Table 1 — Guidelines for accurate and transparent health estimates reporting (GATHER) checklist. [file DataSheet_1.zip › Supplementary Tables/Supplementary Table S6.DOC]

| **Table S6: DALYs due to type 2 diabetes in 1990 and 2019 for both sexes and percentage change in age-standardised rates (ASRs) per 100,000 by location**  **(Generated from data available from http://ghdx.healthdata.org/gbd-results-tool)** | | | | | |
| --- | --- | --- | --- | --- | --- |
|  | **1990** | | **2019** | | **Percentage change in ASRs per 100,000** |
|  | **No (95% UI)** | **ASRs per 100,000 (95% UI)** | **No (95% UI)** | **ASRs per 100,000 (95% UI)** |  |
| **Global** | **25478098 (21701411 , 29776367)** | **628.3 (537.2 , 730.9)** | **66299751 (55477042 , 79005166)** | **801.5 (670.6 , 954.4)** | **27.6 (22 , 33)** |
| **High-income North America** | **1993371 (1669346 , 2372413)** | **589.8 (492.5 , 705.4)** | **4392421 (3527813 , 5434957)** | **748.2 (596 , 931.1)** | **26.9 (19.4 , 33.6)** |
| **Canada** | **105857 (92058 , 122239)** | **326.1 (284 , 376.4)** | **264599 (207303 , 330044)** | **399.7 (310.6 , 503.4)** | **22.5 (9 , 36.5)** |
| **Greenland** | **159 (136 , 186)** | **440.8 (380.6 , 512.6)** | **332 (259 , 419)** | **466.1 (364.8 , 585.3)** | **5.7 (-14.2 , 26.4)** |
| **United States of America** | **1887310 (1578351 , 2254702)** | **618.8 (515.6 , 740.8)** | **4127419 (3314503 , 5112093)** | **790 (628.6 , 981.5)** | **27.7 (20.4 , 34.3)** |
| **Australasia** | **84064 (71091 , 99258)** | **362.1 (306.1 , 426.6)** | **198505 (152964 , 250250)** | **419.6 (320.7 , 531.7)** | **15.9 (4.6 , 27.8)** |
| **Australia** | **70349 (59188 , 83500)** | **363.1 (305.5 , 430.3)** | **169764 (129793 , 216151)** | **424.8 (321.7 , 543.9)** | **17 (5 , 29.9)** |
| **New Zealand** | **13716 (11882 , 15876)** | **358.5 (310.1 , 414.7)** | **28742 (23108 , 35183)** | **392.2 (313.3 , 483.1)** | **9.4 (-1.2 , 19.5)** |
| **High-income Asia Pacific** | **781892 (636377 , 956471)** | **383 (312.7 , 469.1)** | **1439312 (1080380 , 1851528)** | **383.2 (285.3 , 495.5)** | **0 (-8.9 , 8)** |
| **Brunei Darussalam** | **3035 (2593 , 3607)** | **2988 (2579.3 , 3512.7)** | **7549 (6240 , 9114)** | **2343.2 (1978 , 2775.8)** | **-21.6 (-32.2 , -9.9)** |
| **Japan** | **552719 (440112 , 688889)** | **325.9 (259.7 , 405.2)** | **868353 (635821 , 1156922)** | **313.8 (226.3 , 421)** | **-3.7 (-13.2 , 4.7)** |
| **Singapore** | **18713 (15232 , 22912)** | **796.2 (649.2 , 967.3)** | **38066 (26373 , 52496)** | **473.4 (327.8 , 650.5)** | **-40.5 (-50.1 , -31)** |
| **Republic of Korea** | **207425 (176501 , 243951)** | **642.7 (548.8 , 769)** | **525344 (410940 , 652314)** | **596.6 (466.5 , 740.6)** | **-7.2 (-19 , 1.9)** |
| **Western Europe** | **2606182 (2182532 , 3105499)** | **458.4 (381.2 , 551.6)** | **4248596 (3267567 , 5403489)** | **515.8 (389.5 , 663.9)** | **12.5 (1.8 , 21.3)** |
| **Andorra** | **157 (120 , 204)** | **285.7 (219.8 , 367)** | **532 (393 , 706)** | **387.4 (283.9 , 514.2)** | **35.6 (13.6 , 54.8)** |
| **Austria** | **44436 (37716 , 52756)** | **378.8 (319.4 , 453.1)** | **82714 (65386 , 104086)** | **487 (378.2 , 623.1)** | **28.6 (16.8 , 40.2)** |
| **Belgium** | **60043 (48165 , 74142)** | **398.6 (318 , 496.4)** | **93818 (69034 , 123339)** | **452 (327.2 , 600.3)** | **13.4 (1.5 , 25)** |
| **Cyprus** | **10094 (8507 , 11891)** | **1282.7 (1081.3 , 1499.3)** | **15273 (12285 , 18868)** | **794.8 (639.9 , 979.3)** | **-38 (-47.2 , -28.1)** |
| **Denmark** | **23130 (20063 , 26774)** | **303.8 (261.2 , 353.7)** | **44297 (36523 , 53849)** | **417.5 (338.8 , 512.5)** | **37.4 (25.3 , 48.8)** |
| **Finland** | **25967 (19978 , 33117)** | **376.1 (288.5 , 478.6)** | **50288 (35951 , 67597)** | **489.4 (343.8 , 664.1)** | **30.1 (16 , 42.1)** |
| **France** | **184658 (156666 , 216536)** | **225.6 (190.7 , 266.3)** | **356114 (289358 , 435072)** | **278.2 (220.6 , 345.7)** | **23.3 (12.7 , 34.2)** |
| **Germany** | **695999 (574869 , 847430)** | **558.5 (457.1 , 685.7)** | **1032453 (786681 , 1323075)** | **602.2 (453.2 , 783.7)** | **7.8 (-3 , 17.8)** |
| **Greece** | **51431 (40126 , 64853)** | **342.1 (265.3 , 429.2)** | **93511 (68148 , 123137)** | **456.6 (329.6 , 610.3)** | **33.5 (20.3 , 45.6)** |
| **Iceland** | **672 (521 , 870)** | **240 (185.4 , 311.2)** | **1948 (1417 , 2623)** | **377.8 (273.1 , 509.8)** | **57.4 (43.3 , 70.1)** |
| **Ireland** | **11238 (9678 , 13067)** | **275.5 (236.1 , 322.8)** | **28039 (20613 , 36991)** | **388.7 (284.4 , 515.5)** | **41.1 (19.1 , 62)** |
| **Israel** | **24713 (20983 , 29072)** | **514 (436.5 , 604)** | **76580 (62796 , 92954)** | **674.8 (549.6 , 825.6)** | **31.3 (22.7 , 39)** |
| **Italy** | **514775 (443901 , 598154)** | **583.4 (499.4 , 680.7)** | **774156 (616354 , 953118)** | **586.6 (455.5 , 742.8)** | **0.6 (-9.1 , 9.1)** |
| **Luxembourg** | **1611 (1363 , 1909)** | **299.5 (251.9 , 356.8)** | **5345 (3861 , 7113)** | **556.2 (399.4 , 741.6)** | **85.7 (55.1 , 115)** |
| **Malta** | **2952 (2492 , 3513)** | **693.4 (584.8 , 824.4)** | **5412 (4195 , 6922)** | **629.4 (483.9 , 806)** | **-9.2 (-19 , 0.1)** |
| **Monaco** | **131 (98 , 170)** | **205.1 (151.5 , 269.2)** | **287 (204 , 387)** | **350.1 (247.1 , 472.4)** | **70.6 (55 , 85.6)** |
| **Netherlands** | **97553 (82691 , 114160)** | **494.5 (419 , 579.3)** | **127870 (98495 , 162894)** | **401 (304.7 , 516.6)** | **-18.9 (-28.4 , -7.9)** |
| **Norway** | **23290 (18382 , 29089)** | **370.4 (290 , 466.9)** | **38572 (29094 , 49844)** | **438.5 (327.3 , 572.1)** | **18.4 (11.5 , 23.2)** |
| **Portugal** | **91611 (78379 , 108054)** | **654.8 (558.4 , 774.1)** | **153284 (120020 , 192196)** | **684.5 (524.6 , 868.4)** | **4.5 (-6.4 , 14.6)** |
| **San Marino** | **94 (73 , 120)** | **288.5 (224 , 367.5)** | **244 (181 , 321)** | **415.2 (303 , 550.9)** | **43.9 (24.4 , 62.1)** |
| **Spain** | **331060 (268529 , 406520)** | **606 (490.7 , 743.5)** | **492079 (362602 , 651362)** | **553.1 (398 , 741.3)** | **-8.7 (-19.8 , 2.1)** |
| **Sweden** | **46277 (38159 , 55790)** | **328.5 (266.8 , 397.9)** | **77184 (60939 , 96268)** | **408 (313.6 , 518.1)** | **24.2 (14.3 , 33.5)** |
| **Switzerland** | **44500 (36954 , 54126)** | **431.6 (355.4 , 528.9)** | **65706 (49296 , 86005)** | **405.9 (300.5 , 546.3)** | **-5.9 (-17 , 4.6)** |
| **United Kingdom** | **317629 (256414 , 392496)** | **370.2 (295.3 , 461.2)** | **629186 (457035 , 830862)** | **589.2 (419.7 , 787.8)** | **59.1 (41.3 , 73.8)** |
| **Southern Latin America** | **281353 (245372 , 325507)** | **606.3 (528.9 , 700.2)** | **593920 (484526 , 720792)** | **722.4 (588.2 , 878.5)** | **19.1 (9.6 , 27.8)** |
| **Argentina** | **207836 (182197 , 239082)** | **640.4 (561.9 , 737.3)** | **395929 (326728 , 479159)** | **746.3 (614 , 908.9)** | **16.5 (7.2 , 25.5)** |
| **Chile** | **57767 (48393 , 68804)** | **573.9 (482.7 , 683.1)** | **170570 (132942 , 213283)** | **711.6 (555.8 , 890.8)** | **24 (12.3 , 34.8)** |
| **Uruguay** | **15739 (14450 , 17243)** | **401.3 (368.2 , 439.2)** | **27391 (22887 , 32699)** | **529.1 (440.2 , 637.3)** | **31.8 (16.6 , 48.4)** |
| **Eastern Europe** | **778635 (615020 , 961480)** | **281.5 (221.9 , 347.8)** | **1253990 (989655 , 1562223)** | **376 (295.1 , 468.2)** | **33.6 (28.1 , 39.2)** |
| **Belarus** | **38291 (29522 , 48522)** | **298.9 (229.9 , 379)** | **41994 (29620 , 57038)** | **278.7 (194.9 , 378.1)** | **-6.8 (-16.8 , 3)** |
| **Estonia** | **5827 (4343 , 7555)** | **291.4 (217.3 , 379.7)** | **9682 (7068 , 12797)** | **421.8 (305.5 , 562.9)** | **44.8 (33 , 56.6)** |
| **Latvia** | **10938 (8458 , 13951)** | **314.7 (242.3 , 400.4)** | **17206 (13287 , 21928)** | **491.7 (375.8 , 637)** | **56.3 (42.1 , 70.5)** |
| **Lithuania** | **12127 (9230 , 15618)** | **273.6 (208.2 , 352.1)** | **16972 (12596 , 22121)** | **339.7 (252 , 447.5)** | **24.2 (14.5 , 34.2)** |
| **Republic of Moldova** | **20130 (15882 , 25349)** | **443.2 (349.9 , 557.9)** | **26840 (19519 , 35423)** | **478.9 (346.4 , 633.7)** | **8.1 (-2.5 , 17.4)** |
| **Russian Federation** | **485699 (387256 , 599336)** | **270.4 (215.1 , 333.2)** | **893782 (711033 , 1105598)** | **387.9 (307 , 480.3)** | **43.5 (36.1 , 51.9)** |
| **Ukraine** | **205623 (160561 , 259796)** | **296.9 (231.7 , 374.4)** | **247515 (187735 , 319580)** | **351 (266.8 , 451.7)** | **18.2 (8.8 , 27.5)** |
| **Central Europe** | **857125 (694759 , 1037954)** | **580.4 (470.4 , 703.7)** | **1479319 (1147040 , 1854628)** | **730.2 (559 , 923.1)** | **25.8 (17 , 32.8)** |
| **Albania** | **5621 (4260 , 7327)** | **262.5 (200.4 , 344.1)** | **14435 (10357 , 19510)** | **348.7 (249.3 , 469.8)** | **32.9 (20.1 , 44.5)** |
| **Bosnia and Herzegovina** | **24729 (20005 , 30223)** | **602.2 (489.4 , 728.2)** | **76453 (60311 , 95077)** | **1293.9 (1026.2 , 1598.4)** | **114.9 (84.4 , 144.1)** |
| **Bulgaria** | **80050 (66415 , 95413)** | **638.4 (527.5 , 764.8)** | **98745 (76662 , 124008)** | **729.6 (564.1 , 924.5)** | **14.3 (1.2 , 26.6)** |
| **Croatia** | **36085 (28752 , 45057)** | **566.9 (451.9 , 705.9)** | **57459 (43377 , 74487)** | **698.2 (522.4 , 909.5)** | **23.2 (10.8 , 35.2)** |
| **Czechia** | **90553 (70430 , 114256)** | **666.8 (516.5 , 842.4)** | **200118 (149259 , 259901)** | **1016.5 (754 , 1325.5)** | **52.4 (39.7 , 64.5)** |
| **Hungary** | **84040 (67166 , 104247)** | **579.7 (461.2 , 718.4)** | **133853 (102925 , 171001)** | **744.8 (568.6 , 962)** | **28.5 (17.1 , 40.4)** |
| **Montenegro** | **3789 (2928 , 4738)** | **606.2 (471.3 , 755.2)** | **8191 (6210 , 10483)** | **848.8 (642.3 , 1090.1)** | **40 (28.5 , 52.3)** |
| **North Macedonia** | **14295 (11583 , 17310)** | **758.9 (621 , 918)** | **40464 (31979 , 50490)** | **1255.9 (994.8 , 1561.5)** | **65.5 (46.3 , 86)** |
| **Poland** | **277933 (227942 , 336395)** | **634.8 (520.2 , 768.2)** | **463607 (353175 , 588122)** | **708.2 (535.5 , 902.5)** | **11.6 (2 , 18.9)** |
| **Romania** | **109066 (86392 , 136071)** | **387.9 (306.5 , 483.9)** | **165625 (125857 , 214104)** | **482.7 (364.6 , 625)** | **24.4 (12.7 , 35.8)** |
| **Serbia** | **86013 (69936 , 104071)** | **756 (617.4 , 911)** | **149270 (118066 , 185389)** | **962.8 (749.6 , 1206.4)** | **27.4 (12.4 , 42.7)** |
| **Slovakia** | **32847 (26623 , 39939)** | **549.3 (444.3 , 667.4)** | **50209 (37670 , 65507)** | **561 (419.5 , 734.7)** | **2.1 (-10.3 , 14)** |
| **Slovenia** | **12103 (9365 , 15402)** | **499.4 (385.6 , 633.9)** | **20889 (15061 , 27254)** | **524.6 (370.8 , 690.1)** | **5.1 (-9 , 17.7)** |
| **Central Asia** | **226090 (184508 , 273319)** | **460.4 (375.3 , 556.8)** | **809249 (665764 , 976700)** | **1013.8 (841.9 , 1219.1)** | **120.2 (106.9 , 137.6)** |
| **Armenia** | **18733 (16085 , 21991)** | **665.1 (571.6 , 779.6)** | **44262 (36382 , 53198)** | **1064.6 (874.1 , 1278)** | **60.1 (43.6 , 76.9)** |
| **Azerbaijan** | **26943 (22219 , 32389)** | **503.4 (416.6 , 603.1)** | **99253 (79601 , 121571)** | **971.3 (781.1 , 1180.5)** | **93 (71.1 , 116.7)** |
| **Georgia** | **30091 (24380 , 36481)** | **479.4 (387.7 , 583.3)** | **57555 (45882 , 71084)** | **1012.3 (801.6 , 1256.1)** | **111.2 (90.7 , 131.7)** |
| **Kazakhstan** | **62004 (47868 , 79230)** | **470.3 (363.2 , 596)** | **153586 (118906 , 194947)** | **859.6 (670 , 1081.7)** | **82.8 (68.9 , 98.4)** |
| **Kyrgyzstan** | **10764 (8665 , 13201)** | **342.4 (275.8 , 420.2)** | **22591 (17351 , 28804)** | **446.6 (345 , 565.8)** | **30.5 (19 , 41.1)** |
| **Mongolia** | **2589 (2008 , 3223)** | **227.6 (176 , 282.2)** | **8681 (6547 , 11322)** | **316 (240.3 , 409.4)** | **38.8 (20.3 , 58.5)** |
| **Tajikistan** | **15740 (13317 , 18591)** | **526.6 (446.4 , 617.6)** | **70906 (57027 , 87318)** | **1223.3 (994.8 , 1494.6)** | **132.3 (98 , 168.8)** |
| **Turkmenistan** | **9873 (8242 , 11817)** | **472.6 (395.1 , 565.8)** | **36954 (29245 , 46847)** | **842.2 (669.4 , 1059.3)** | **78.2 (57.4 , 102.7)** |
| **Uzbekistan** | **49352 (41290 , 58442)** | **407.1 (341.8 , 480.2)** | **315461 (262691 , 378519)** | **1301.7 (1093.5 , 1551.5)** | **219.8 (184.5 , 263.1)** |
| **Central Latin America** | **1387852 (1209539 , 1588299)** | **1575.6 (1375.8 , 1799.4)** | **4206491 (3578322 , 4997810)** | **1746.5 (1485.3 , 2073.9)** | **10.8 (2.9 , 18.8)** |
| **Colombia** | **173517 (139966 , 210510)** | **935 (758.4 , 1139.5)** | **465299 (351443 , 596714)** | **881.6 (666.3 , 1132.5)** | **-5.7 (-16.6 , 4.6)** |
| **Costa Rica** | **14407 (11549 , 17814)** | **797.2 (642.1 , 984.6)** | **45854 (34105 , 60169)** | **884.5 (658 , 1159.4)** | **11 (-0.8 , 22.8)** |
| **El Salvador** | **23271 (19323 , 27614)** | **755.9 (627.6 , 892.8)** | **88055 (69429 , 108503)** | **1501.1 (1182.1 , 1851.7)** | **98.6 (66.6 , 134.7)** |
| **Guatemala** | **30001 (24469 , 36556)** | **759.8 (618.5 , 921.4)** | **253586 (204734 , 306356)** | **2172.3 (1750.2 , 2620.2)** | **185.9 (142 , 240.6)** |
| **Honduras** | **18261 (14073 , 22894)** | **801.9 (615.6 , 1006.5)** | **76663 (58566 , 97767)** | **1174.1 (898.8 , 1492.3)** | **46.4 (33.2 , 62.3)** |
| **Mexico** | **978244 (867745 , 1102004)** | **2167.7 (1927.5 , 2439)** | **2716393 (2316224 , 3202412)** | **2261.1 (1935.9 , 2664.1)** | **4.3 (-4.7 , 13.4)** |
| **Nicaragua** | **18338 (15423 , 21868)** | **1122.2 (948.3 , 1336.5)** | **77017 (62457 , 94493)** | **1664.3 (1366.6 , 2032.4)** | **48.3 (32.1 , 66.7)** |
| **Panama** | **12246 (9910 , 15036)** | **803.1 (654.1 , 984.5)** | **52453 (41188 , 65283)** | **1262.9 (991.5 , 1573.4)** | **57.3 (39.8 , 77.5)** |
| **Venezuela (Bolivarian Republic of)** | **119566 (102902 , 138943)** | **1181 (1021.8 , 1372.1)** | **431171 (339934 , 530901)** | **1456.7 (1152.1 , 1792.3)** | **23.3 (5.6 , 43.7)** |
| **Andean Latin America** | **137743 (118249 , 160688)** | **653.6 (562.9 , 763.7)** | **493570 (408117 , 592310)** | **873.5 (722.3 , 1045.3)** | **33.6 (17.1 , 49.3)** |
| **Bolivia (Plurinational State of)** | **37882 (31456 , 46644)** | **1143.6 (954.1 , 1403.8)** | **119118 (94785 , 147129)** | **1329.9 (1059.8 , 1638.9)** | **16.3 (-4.3 , 41.6)** |
| **Ecuador** | **40983 (35494 , 47596)** | **745.9 (646.3 , 865.7)** | **177086 (143589 , 216421)** | **1162.5 (945.2 , 1418.2)** | **55.8 (34.3 , 81)** |
| **Peru** | **58879 (48812 , 70163)** | **480.4 (397.1 , 571.2)** | **197366 (155841 , 245412)** | **612.8 (484.3 , 761.3)** | **27.5 (6.9 , 52.8)** |
| **Caribbean** | **374011 (325010 , 431612)** | **1407.9 (1222.5 , 1622.5)** | **766883 (633840 , 930743)** | **1483.1 (1226 , 1800.5)** | **5.3 (-6 , 16.5)** |
| **Antigua and Barbuda** | **894 (793 , 1014)** | **1715.8 (1519 , 1946.9)** | **1953 (1613 , 2327)** | **1905.9 (1573.7 , 2245.3)** | **11.1 (-0.8 , 23)** |
| **Barbados** | **5339 (4745 , 5968)** | **1876 (1659.2 , 2111.3)** | **8865 (7330 , 10542)** | **1860.3 (1540.4 , 2213.4)** | **-0.8 (-12.7 , 11)** |
| **Belize** | **1195 (1050 , 1370)** | **1253.5 (1100.8 , 1424.9)** | **5442 (4637 , 6381)** | **1848.4 (1584 , 2154.9)** | **47.5 (32.6 , 64.1)** |
| **Bermuda** | **595 (510 , 687)** | **948.2 (814.7 , 1092)** | **967 (767 , 1204)** | **795.2 (626.7 , 990)** | **-16.1 (-26.9 , -5.5)** |
| **Bahamas** | **2502 (2168 , 2891)** | **1551.4 (1350.9 , 1780.8)** | **6273 (5022 , 7788)** | **1551.1 (1250.2 , 1918.2)** | **0 (-13.1 , 12.9)** |
| **Cuba** | **101903 (83215 , 123539)** | **986.6 (804.4 , 1196.7)** | **159686 (118661 , 209676)** | **885.5 (655.7 , 1164.9)** | **-10.2 (-21 , 0)** |
| **Dominica** | **1318 (1152 , 1506)** | **1899.3 (1645.8 , 2173.3)** | **1912 (1560 , 2323)** | **2164.5 (1767 , 2632.9)** | **14 (-1.8 , 30.8)** |
| **Dominican Republic** | **28404 (24170 , 32944)** | **700.5 (594.5 , 811.9)** | **107868 (85148 , 133560)** | **1119.1 (885 , 1385.1)** | **59.7 (31.7 , 92.7)** |
| **Grenada** | **1410 (1242 , 1595)** | **2036.9 (1788 , 2306.3)** | **2617 (2229 , 3075)** | **2313.4 (1978.1 , 2701.3)** | **13.6 (2.8 , 23.7)** |
| **Guyana** | **10339 (8957 , 11909)** | **2519.4 (2199.7 , 2878)** | **20601 (16537 , 24961)** | **3099.4 (2501.4 , 3746)** | **23 (1.6 , 48.2)** |
| **Haiti** | **83470 (66526 , 102521)** | **2368 (1897.5 , 2870.6)** | **164464 (123331 , 213890)** | **2178.4 (1654.3 , 2860.6)** | **-8 (-25.2 , 12.8)** |
| **Jamaica** | **30282 (27309 , 33656)** | **1721 (1554.2 , 1914.1)** | **67418 (55515 , 80861)** | **2261 (1858.4 , 2714.3)** | **31.4 (13.3 , 51.5)** |
| **Puerto Rico** | **53537 (45529 , 62898)** | **1481.5 (1260 , 1735.8)** | **109969 (88272 , 135650)** | **1706.3 (1363.3 , 2117.2)** | **15.2 (1.4 , 30.3)** |
| **Saint Kitts and Nevis** | **745 (654 , 846)** | **2068 (1826.5 , 2358.9)** | **1290 (1049 , 1540)** | **1886.1 (1557.5 , 2241.1)** | **-8.8 (-19.8 , 3.2)** |
| **Saint Lucia** | **2243 (1955 , 2551)** | **2538.2 (2214.9 , 2878.6)** | **4896 (3998 , 5928)** | **2269.5 (1861.8 , 2742.1)** | **-10.6 (-20.6 , -0.5)** |
| **Saint Vincent and the Grenadines** | **1735 (1538 , 1958)** | **2407.7 (2135.3 , 2720.2)** | **3531 (3007 , 4140)** | **2610.4 (2224.4 , 3059.6)** | **8.4 (-2.9 , 19.8)** |
| **Suriname** | **3711 (3138 , 4383)** | **1369.3 (1159.6 , 1616.7)** | **12188 (9908 , 14833)** | **1977.1 (1611.8 , 2404.3)** | **44.4 (29.4 , 61)** |
| **Trinidad and Tobago** | **30802 (27912 , 34013)** | **3619 (3286.9 , 3987.2)** | **58064 (46008 , 71994)** | **3100.1 (2462.6 , 3828.1)** | **-14.3 (-29.7 , 2.7)** |
| **United States Virgin Islands** | **1128 (947 , 1322)** | **1287.3 (1088.7 , 1502.5)** | **2900 (2380 , 3468)** | **1635 (1343.9 , 1964.2)** | **27 (10.8 , 44.4)** |
| **Tropical Latin America** | **1033036 (904574 , 1179390)** | **1082.2 (952.2 , 1232.6)** | **2492517 (2113099 , 2920318)** | **1020.4 (868 , 1194.5)** | **-5.7 (-10.1 , -1.9)** |
| **Brazil** | **1016120 (889834 , 1159971)** | **1090.6 (959.5 , 1243.9)** | **2414757 (2046263 , 2836619)** | **1012 (859.2 , 1187.5)** | **-7.2 (-11.6 , -3.4)** |
| **Paraguay** | **16916 (14261 , 19913)** | **749.2 (632.2 , 876.1)** | **77760 (62463 , 94520)** | **1385.3 (1113.1 , 1681.8)** | **84.9 (54.7 , 122.9)** |
| **East Asia** | **4159484 (3341658 , 5121387)** | **447.2 (361.6 , 546.9)** | **10164521 (8071056 , 12540885)** | **487.6 (387.8 , 602.7)** | **9.1 (0.3 , 17.5)** |
| **China** | **3914033 (3129815 , 4846143)** | **435.8 (351 , 536.3)** | **9603363 (7588721 , 11904723)** | **476.5 (376.4 , 589.5)** | **9.3 (0.1 , 18.3)** |
| **Democratic People's Republic of Korea** | **88336 (68879 , 109669)** | **519.4 (411.7 , 641.8)** | **198410 (155156 , 252245)** | **611 (478.9 , 772.9)** | **17.6 (-0.8 , 37.4)** |
| **Taiwan (Province of China)** | **157114 (136842 , 181288)** | **966.5 (848.4 , 1107.6)** | **362749 (289656 , 450998)** | **925.7 (737.4 , 1155)** | **-4.2 (-16.5 , 9.5)** |
| **Southeast Asia** | **2743837 (2407787 , 3112894)** | **1002.9 (885.2 , 1132.4)** | **8079981 (7008927 , 9255221)** | **1273.4 (1103.9 , 1452.4)** | **27 (14.9 , 40)** |
| **Cambodia** | **45541 (36246 , 55318)** | **929.8 (752.6 , 1117.7)** | **142498 (113436 , 177726)** | **1158.9 (928.7 , 1436.5)** | **24.6 (1.5 , 51.4)** |
| **Indonesia** | **1085181 (941531 , 1234983)** | **982.5 (856.9 , 1110.4)** | **3556369 (3005869 , 4057138)** | **1494 (1272.6 , 1692.4)** | **52.1 (30.1 , 75.9)** |
| **Lao People's Democratic Republic** | **27731 (21417 , 35963)** | **1234.3 (966.9 , 1574)** | **63214 (49753 , 78640)** | **1363.5 (1089.6 , 1690.2)** | **10.5 (-14.8 , 43.4)** |
| **Malaysia** | **92599 (78765 , 108114)** | **956.7 (818.6 , 1112.2)** | **227262 (173032 , 292445)** | **819.8 (625.6 , 1048)** | **-14.3 (-27.2 , -1)** |
| **Maldives** | **926 (754 , 1181)** | **985 (815 , 1219.5)** | **2559 (2010 , 3232)** | **785.3 (625.8 , 989.1)** | **-20.3 (-36.2 , -3.9)** |
| **Mauritius** | **12139 (10765 , 13855)** | **1563.5 (1389.9 , 1781.8)** | **57569 (47491 , 68082)** | **3239.6 (2678.4 , 3824.7)** | **107.2 (78.1 , 139.9)** |
| **Myanmar** | **398330 (305981 , 506959)** | **1599.7 (1249.3 , 1993.5)** | **756044 (627538 , 914435)** | **1587.1 (1329.7 , 1890.3)** | **-0.8 (-22 , 26)** |
| **Philippines** | **330700 (291676 , 372698)** | **1012.9 (890.6 , 1145)** | **977808 (833785 , 1148974)** | **1195.5 (1024.2 , 1403.1)** | **18 (2.8 , 35.8)** |
| **Sri Lanka** | **95732 (80176 , 113756)** | **884.9 (744.8 , 1045.6)** | **475544 (376233 , 588438)** | **1881.2 (1499.6 , 2323.2)** | **112.6 (77.1 , 152.8)** |
| **Seychelles** | **372 (296 , 460)** | **657.3 (525.1 , 813.7)** | **1508 (1159 , 1916)** | **1345.6 (1036.1 , 1705.6)** | **104.7 (88.4 , 121.4)** |
| **Thailand** | **309662 (261795 , 364249)** | **825.6 (705 , 966.8)** | **833465 (651859 , 1033448)** | **818.5 (640 , 1017.9)** | **-0.9 (-18 , 19.5)** |
| **Timor-Leste** | **2232 (1762 , 2799)** | **699.4 (562.9 , 861.4)** | **7856 (6013 , 9912)** | **947.1 (733.8 , 1188.4)** | **35.4 (8.9 , 62.3)** |
| **Viet Nam** | **339046 (274585 , 411607)** | **844 (685.1 , 1018)** | **967700 (774604 , 1176850)** | **1058.5 (849.1 , 1279.7)** | **25.4 (2.5 , 53.4)** |
| **Oceania** | **86843 (73005 , 103170)** | **2592.7 (2186.1 , 3065.5)** | **298125 (243971 , 356983)** | **3703.4 (3060 , 4399.3)** | **42.8 (17.7 , 70.2)** |
| **American Samoa** | **677 (570 , 802)** | **2704.4 (2302.7 , 3174.4)** | **1868 (1541 , 2247)** | **3725.5 (3080.3 , 4463.2)** | **37.8 (20.4 , 56)** |
| **Cook Islands** | **421 (361 , 492)** | **3221.9 (2782.1 , 3737.1)** | **810 (687 , 954)** | **3341.8 (2821.7 , 3960)** | **3.7 (-14.1 , 24)** |
| **Micronesia (Federated States of)** | **1366 (1085 , 1695)** | **2718.2 (2192.3 , 3344.8)** | **3824 (2802 , 5283)** | **4896.3 (3682.2 , 6620.4)** | **80.1 (27.5 , 134.5)** |
| **Fiji** | **17200 (14182 , 21044)** | **4357.6 (3605.7 , 5323.4)** | **54197 (44219 , 65145)** | **6884.3 (5667.8 , 8214.8)** | **58 (20.7 , 104.5)** |
| **Guam** | **994 (835 , 1174)** | **1213.5 (1032.6 , 1424.2)** | **2274 (1814 , 2837)** | **1195.5 (951.9 , 1488.8)** | **-1.5 (-15.3 , 14.6)** |
| **Kiribati** | **1875 (1546 , 2231)** | **4432.9 (3665.6 , 5242)** | **4930 (3854 , 6148)** | **6161.4 (4876.7 , 7611.3)** | **39 (4.8 , 76.4)** |
| **Marshall Islands** | **508 (421 , 614)** | **2804.1 (2342.6 , 3381.6)** | **1685 (1315 , 2146)** | **4191.3 (3300.7 , 5261.9)** | **49.5 (25.4 , 77)** |
| **Nauru** | **140 (110 , 191)** | **3130.8 (2510.3 , 4072.7)** | **244 (188 , 355)** | **4673.7 (3709.5 , 6470.2)** | **49.3 (22.1 , 80.8)** |
| **Niue** | **57 (47 , 69)** | **2665.8 (2205.3 , 3264.1)** | **85 (68 , 104)** | **3960 (3166.8 , 4853.5)** | **48.6 (20.8 , 78)** |
| **Northern Mariana Islands** | **348 (284 , 419)** | **1583.2 (1323.4 , 1873.4)** | **1171 (972 , 1421)** | **2076.3 (1736.7 , 2488.5)** | **31.1 (12.1 , 49.6)** |
| **Palau** | **246 (200 , 299)** | **2367.7 (1937 , 2862)** | **868 (691 , 1070)** | **3760.8 (3008.5 , 4602.5)** | **58.8 (24 , 99.7)** |
| **Papua New Guinea** | **49420 (39470 , 60632)** | **2331.4 (1874.9 , 2849.2)** | **182916 (145542 , 224595)** | **3260.5 (2613 , 3989.3)** | **39.9 (11.4 , 72.7)** |
| **Samoa** | **1947 (1572 , 2404)** | **2138.7 (1738.4 , 2629.4)** | **4419 (3606 , 5441)** | **2887.1 (2369.9 , 3536.5)** | **35 (8.3 , 67.5)** |
| **Solomon Islands** | **4159 (3156 , 5681)** | **2513.1 (1927.6 , 3388.8)** | **16920 (13815 , 20780)** | **4391.7 (3647.6 , 5319.3)** | **74.7 (24.6 , 133.7)** |
| **Tokelau** | **30 (24 , 36)** | **2250.7 (1812.8 , 2744.2)** | **41 (32 , 51)** | **3024.7 (2420.9 , 3766.6)** | **34.4 (5.1 , 66.7)** |
| **Tonga** | **1408 (1195 , 1639)** | **2433.6 (2073.4 , 2830.9)** | **2493 (2041 , 3017)** | **3112.6 (2552.6 , 3768.9)** | **27.9 (5.8 , 56.2)** |
| **Tuvalu** | **187 (155 , 230)** | **2597.9 (2159.1 , 3169.2)** | **375 (293 , 492)** | **3551.1 (2794.1 , 4647.2)** | **36.7 (7.3 , 76.6)** |
| **Vanuatu** | **1056 (808 , 1425)** | **1466 (1131.1 , 1965.7)** | **4927 (3817 , 6294)** | **2641.7 (2061.8 , 3351.2)** | **80.2 (39.9 , 130.3)** |
| **North Africa and Middle East** | **1429598 (1229348 , 1675907)** | **808.3 (696.9 , 944.3)** | **4806918 (3927069 , 5857180)** | **1060.8 (872.1 , 1279.1)** | **31.2 (18.3 , 42.2)** |
| **Afghanistan** | **73967 (52412 , 102293)** | **997.1 (713.4 , 1373.5)** | **222032 (160451 , 295439)** | **1567.9 (1136.3 , 2073)** | **57.3 (26.8 , 88.1)** |
| **Algeria** | **79090 (58771 , 102412)** | **641.4 (485.4 , 828.7)** | **329574 (249708 , 430895)** | **951 (727.9 , 1234.6)** | **48.3 (26.4 , 71.6)** |
| **Bahrain** | **3654 (3058 , 4311)** | **2067.6 (1751.7 , 2415.8)** | **31385 (25064 , 39006)** | **3232.5 (2622.4 , 3929.3)** | **56.3 (28.4 , 83.8)** |
| **Egypt** | **219146 (193502 , 248721)** | **718.2 (637.2 , 811.6)** | **835076 (661795 , 1022770)** | **1224.7 (973 , 1492.8)** | **70.5 (43.2 , 102.9)** |
| **Iran (Islamic Republic of)** | **138646 (111692 , 170125)** | **516.5 (420.7 , 628.2)** | **716457 (578136 , 882592)** | **958.1 (776.6 , 1170.2)** | **85.5 (67 , 99.8)** |
| **Iraq** | **128267 (107542 , 151909)** | **1544 (1292.5 , 1824.7)** | **400206 (319445 , 489469)** | **1625 (1314.8 , 1979.3)** | **5.2 (-13.9 , 25.2)** |
| **Jordan** | **21551 (18283 , 25268)** | **1624.7 (1380.1 , 1899.1)** | **86617 (70835 , 107107)** | **1292.9 (1069.4 , 1569.3)** | **-20.4 (-33.5 , -5.5)** |
| **Kuwait** | **7481 (5929 , 9359)** | **1072.4 (877.3 , 1311)** | **35526 (25657 , 47276)** | **1136.3 (841.6 , 1480)** | **6 (-5.9 , 17.2)** |
| **Lebanon** | **18443 (14745 , 22808)** | **801.8 (649.5 , 990.4)** | **51734 (38476 , 67616)** | **991.2 (737.8 , 1295)** | **23.6 (5.2 , 39.6)** |
| **Libya** | **13403 (10317 , 16938)** | **686.8 (531.8 , 866.7)** | **62338 (46425 , 81547)** | **1138.8 (859.7 , 1475.2)** | **65.8 (43.5 , 92.1)** |
| **Morocco** | **77215 (61491 , 97915)** | **546.7 (436.7 , 695.7)** | **325869 (252352 , 408280)** | **1008.6 (786.9 , 1261.8)** | **84.5 (60.9 , 106.9)** |
| **Palestine** | **12033 (9685 , 14775)** | **1398.9 (1131.1 , 1700.6)** | **44332 (37713 , 52723)** | **1907.9 (1642.6 , 2233.1)** | **36.4 (13.4 , 65.9)** |
| **Oman** | **8539 (6732 , 10396)** | **1248.3 (992 , 1505.9)** | **29392 (23840 , 36087)** | **1618.5 (1363.6 , 1912.1)** | **29.6 (4.1 , 58.3)** |
| **Qatar** | **2580 (2096 , 3128)** | **2528.1 (2147.5 , 2948.9)** | **28694 (21143 , 37665)** | **2975.3 (2401.2 , 3673.1)** | **17.7 (-2.4 , 40.8)** |
| **Saudi Arabia** | **58772 (46154 , 72873)** | **902.3 (710.1 , 1117)** | **245548 (187706 , 316976)** | **1064.5 (829.7 , 1348.6)** | **18 (-5.1 , 42.3)** |
| **Sudan** | **49524 (37355 , 64065)** | **510.3 (386.5 , 656.2)** | **172228 (126964 , 221856)** | **855.6 (643.6 , 1090.2)** | **67.7 (43.6 , 92.6)** |
| **Syrian Arab Republic** | **41771 (33397 , 51291)** | **740.1 (594.8 , 903)** | **108820 (81226 , 142143)** | **853.2 (646.5 , 1103.2)** | **15.3 (-3.1 , 34.8)** |
| **Tunisia** | **29009 (22160 , 37306)** | **564 (434.2 , 720.3)** | **120897 (90701 , 157871)** | **943.8 (713.8 , 1233.8)** | **67.4 (48 , 86.7)** |
| **Turkey** | **413748 (351292 , 484046)** | **1141.6 (976.1 , 1333.6)** | **770690 (619599 , 956089)** | **869.9 (702.3 , 1073.5)** | **-23.8 (-36.5 , -9.4)** |
| **United Arab Emirates** | **7697 (6225 , 9332)** | **1884.2 (1558.7 , 2246.7)** | **87940 (66705 , 113103)** | **1865.2 (1468 , 2332.4)** | **-1 (-23.8 , 23.8)** |
| **Yemen** | **24099 (18086 , 32145)** | **467.3 (354.5 , 626.4)** | **96676 (72068 , 127175)** | **676.5 (512.3 , 878.7)** | **44.8 (21.8 , 68.9)** |
| **South Asia** | **4284286 (3559600 , 5062547)** | **732 (616.5 , 856.6)** | **15119427 (12449068 , 17998394)** | **1049.7 (869.3 , 1244.9)** | **43.4 (30 , 57.1)** |
| **Bangladesh** | **374388 (310284 , 445313)** | **763.1 (642.3 , 893.1)** | **1095929 (886461 , 1350363)** | **845.1 (688.1 , 1036.2)** | **10.8 (-6.1 , 29.6)** |
| **Bhutan** | **1635 (1253 , 2038)** | **622.9 (484.1 , 767.6)** | **5528 (4380 , 6949)** | **968.3 (768.9 , 1201.6)** | **55.5 (27.7 , 88.7)** |
| **India** | **3386363 (2802934 , 4043664)** | **721.2 (602.5 , 854.7)** | **12012998 (9793464 , 14477029)** | **1027.9 (843.3 , 1231)** | **42.5 (27.5 , 58.5)** |
| **Nepal** | **46430 (35821 , 59091)** | **465.5 (364.9 , 579.2)** | **175155 (134744 , 224250)** | **776.4 (608.7 , 980.9)** | **66.8 (43.6 , 90)** |
| **Pakistan** | **475470 (369269 , 574436)** | **803.6 (621.8 , 961.1)** | **1829817 (1480683 , 2215526)** | **1554.5 (1270.1 , 1863.1)** | **93.5 (60.7 , 133.3)** |
| **Southern Sub-Saharan Africa** | **346784 (306129 , 386638)** | **1226.5 (1085.3 , 1368.3)** | **1063378 (946947 , 1191241)** | **1878 (1679.9 , 2098.8)** | **53.1 (41.6 , 65.1)** |
| **Botswana** | **9010 (7144 , 11425)** | **1549.1 (1243.2 , 1950.2)** | **32917 (25036 , 42045)** | **2377.2 (1843 , 3018.3)** | **53.5 (18.5 , 97.4)** |
| **Lesotho** | **13055 (10878 , 15528)** | **1316.3 (1101.3 , 1553.5)** | **36426 (27251 , 47003)** | **2817.6 (2120.9 , 3606.8)** | **114.1 (61.9 , 177)** |
| **Namibia** | **10967 (9187 , 12796)** | **1497.5 (1255.9 , 1736.9)** | **22814 (17878 , 28160)** | **1634.9 (1293.8 , 2008.8)** | **9.2 (-11.5 , 35.5)** |
| **South Africa** | **264253 (232289 , 296710)** | **1228 (1074.7 , 1378.8)** | **826466 (732937 , 928953)** | **1843.9 (1642.7 , 2065.5)** | **50.2 (38.5 , 61.8)** |
| **Eswatini** | **6151 (4957 , 7379)** | **2088.1 (1683.8 , 2485.4)** | **19715 (14760 , 25596)** | **3347 (2545.6 , 4277.4)** | **60.3 (22.4 , 113.2)** |
| **Zimbabwe** | **43347 (37290 , 49975)** | **1036.4 (894 , 1191)** | **125041 (100340 , 152084)** | **1720.6 (1382.1 , 2083.4)** | **66 (37.9 , 95.3)** |
| **Western Sub-Saharan Africa** | **702960 (603438 , 813067)** | **794.3 (686.6 , 911.4)** | **1911168 (1613993 , 2238675)** | **994.8 (851 , 1148.4)** | **25.2 (9.9 , 41)** |
| **Benin** | **15403 (12957 , 17965)** | **761.7 (641.6 , 884.4)** | **54013 (43137 , 67309)** | **1066 (852.9 , 1312.4)** | **40 (16.8 , 66.9)** |
| **Burkina Faso** | **40621 (33552 , 48358)** | **925.9 (776.3 , 1088.3)** | **102746 (84232 , 125159)** | **1067.2 (883.6 , 1286.3)** | **15.3 (-2.6 , 35)** |
| **Cameroon** | **41694 (33601 , 51061)** | **920.3 (746.3 , 1124.7)** | **166249 (129974 , 212737)** | **1341 (1069.6 , 1686.1)** | **45.7 (12.1 , 83.9)** |
| **Cabo Verde** | **954 (752 , 1195)** | **416.7 (327.3 , 520.1)** | **4464 (3656 , 5393)** | **1042.2 (857.4 , 1260.5)** | **150.1 (97.7 , 186.1)** |
| **Chad** | **20095 (16287 , 24235)** | **705.5 (571.9 , 844.5)** | **60320 (47192 , 74486)** | **1029.3 (813.6 , 1266.3)** | **45.9 (24.2 , 70.7)** |
| **CÃ´te d'Ivoire** | **37092 (31004 , 44114)** | **880.8 (747.1 , 1033.6)** | **126030 (100322 , 155404)** | **1136.2 (924.7 , 1372.4)** | **29 (5.9 , 54.5)** |
| **Gambia** | **2424 (1906 , 2978)** | **676 (536.8 , 824)** | **10348 (8144 , 12706)** | **1057.6 (832.7 , 1297.6)** | **56.4 (23.8 , 98.9)** |
| **Ghana** | **53998 (43977 , 65160)** | **834.9 (689.9 , 989)** | **215388 (176366 , 262523)** | **1287.8 (1065 , 1573.5)** | **54.2 (25.8 , 84.8)** |
| **Guinea** | **25190 (20918 , 29987)** | **754.2 (623.8 , 895)** | **62152 (49947 , 76559)** | **1096.3 (883.5 , 1351.9)** | **45.4 (17.3 , 78.2)** |
| **Guinea-Bissau** | **4872 (3809 , 6209)** | **1141 (900 , 1430.7)** | **10876 (8626 , 13595)** | **1405.9 (1124.9 , 1749.1)** | **23.2 (-1.9 , 53.6)** |
| **Liberia** | **9784 (8069 , 11611)** | **878.8 (732.5 , 1038.9)** | **24336 (18739 , 30651)** | **1128.7 (883.1 , 1412.6)** | **28.4 (5.3 , 58)** |
| **Mali** | **33870 (28012 , 40547)** | **803.2 (666.7 , 952.5)** | **90848 (72556 , 110458)** | **1024.9 (833.8 , 1229.5)** | **27.6 (5.5 , 53.1)** |
| **Mauritania** | **8753 (7297 , 10341)** | **865.6 (722.1 , 1016.4)** | **17935 (13929 , 22120)** | **861.5 (671.5 , 1057.8)** | **-0.5 (-20.2 , 21.7)** |
| **Niger** | **17926 (14259 , 21666)** | **623.9 (499.7 , 749.6)** | **67484 (53527 , 83908)** | **845.7 (674.6 , 1040.3)** | **35.5 (11.2 , 64.5)** |
| **Nigeria** | **340098 (280339 , 406894)** | **775 (646.3 , 920.8)** | **736863 (585174 , 899652)** | **851.2 (691.2 , 1029.1)** | **9.8 (-10.1 , 34.3)** |
| **Sao Tome and Principe** | **311 (248 , 387)** | **483.4 (386.4 , 600.7)** | **748 (586 , 944)** | **687.8 (541.8 , 859.2)** | **42.3 (25.3 , 60.4)** |
| **Senegal** | **29799 (24359 , 35698)** | **906.9 (748 , 1081)** | **94064 (73522 , 115896)** | **1229.3 (975.7 , 1505)** | **35.5 (13.9 , 61)** |
| **Sierra Leone** | **11342 (9193 , 13615)** | **592.3 (481.1 , 708.2)** | **32394 (25370 , 40627)** | **874.6 (694.1 , 1090.4)** | **47.7 (17.7 , 84.5)** |
| **Togo** | **8711 (7225 , 10411)** | **680.3 (567.9 , 808.1)** | **33883 (26579 , 42550)** | **902.6 (719.7 , 1131.3)** | **32.7 (8.6 , 61.7)** |
| **Eastern Sub-Saharan Africa** | **883109 (771483 , 1021266)** | **1122.5 (985.2 , 1286.5)** | **1748901 (1499615 , 2025207)** | **1026.8 (889.8 , 1182.4)** | **-8.5 (-22 , 2.6)** |
| **Burundi** | **29994 (23615 , 39135)** | **1230.3 (976.2 , 1593.5)** | **49968 (39708 , 61714)** | **1035.6 (830.8 , 1261.3)** | **-15.8 (-33.6 , 6.6)** |
| **Comoros** | **2171 (1423 , 2704)** | **968 (661.6 , 1196.7)** | **4717 (3865 , 5775)** | **958.8 (793.9 , 1164.6)** | **-1 (-22.2 , 43.8)** |
| **Djibouti** | **1374 (1073 , 1761)** | **909.3 (721 , 1147.9)** | **7232 (5533 , 9284)** | **1153 (915.2 , 1441.3)** | **26.8 (2.5 , 59.7)** |
| **Eritrea** | **12256 (9390 , 16509)** | **1096.2 (839.9 , 1450.6)** | **36688 (28447 , 47125)** | **1267.8 (1001.3 , 1610.6)** | **15.7 (-9.8 , 47.8)** |
| **Ethiopia** | **330790 (272887 , 418617)** | **1531.9 (1276.6 , 1908.9)** | **381716 (324570 , 441100)** | **903.2 (777.5 , 1033.1)** | **-41 (-55.8 , -27.7)** |
| **Kenya** | **61068 (52939 , 70674)** | **729.1 (638.4 , 835.5)** | **211955 (177064 , 252994)** | **912.4 (771.7 , 1070.7)** | **25.2 (11.7 , 41.1)** |
| **Madagascar** | **45989 (38863 , 54563)** | **853.6 (719.6 , 1006.3)** | **106531 (84964 , 130978)** | **906.8 (729.5 , 1105.7)** | **6.2 (-13.5 , 29.4)** |
| **Malawi** | **43786 (37028 , 50483)** | **1100.9 (941.1 , 1262.2)** | **86326 (71221 , 104612)** | **1131.5 (941.5 , 1358.1)** | **2.8 (-13.4 , 21.1)** |
| **Mozambique** | **60888 (51245 , 71837)** | **986.5 (836.1 , 1157.8)** | **155909 (125708 , 193184)** | **1322.1 (1081.5 , 1611.1)** | **34 (8.6 , 63.7)** |
| **Rwanda** | **40490 (33011 , 49825)** | **1330.3 (1086.5 , 1632.3)** | **60962 (47793 , 76895)** | **991.4 (795.3 , 1253.8)** | **-25.5 (-41.6 , -7.8)** |
| **Somalia** | **32972 (25454 , 42618)** | **1198.9 (946.9 , 1518.7)** | **89778 (70163 , 113913)** | **1251.9 (996.6 , 1563.1)** | **4.4 (-16.1 , 31.9)** |
| **South Sudan** | **22713 (17960 , 28441)** | **935.2 (747.9 , 1160.9)** | **38240 (29499 , 47995)** | **963.6 (748.3 , 1185.7)** | **3 (-17.6 , 28.5)** |
| **United Republic of Tanzania** | **95502 (81887 , 110116)** | **865.2 (746.2 , 987.4)** | **253614 (207347 , 308278)** | **1000.7 (833.2 , 1203.1)** | **15.7 (-2.9 , 38.6)** |
| **Uganda** | **68358 (54788 , 86913)** | **1034.7 (831.1 , 1311.1)** | **176393 (141577 , 220214)** | **1187 (968.3 , 1456.1)** | **14.7 (-4.1 , 36.9)** |
| **Zambia** | **34110 (29009 , 39591)** | **1138.9 (970.5 , 1319.4)** | **87475 (71863 , 107034)** | **1217.6 (1012 , 1467.6)** | **6.9 (-10.9 , 29.4)** |
| **Central Sub-Saharan Africa** | **299841 (254266 , 351059)** | **1248.9 (1071.9 , 1453.3)** | **732558 (597363 , 901737)** | **1265.1 (1049.1 , 1533.2)** | **1.3 (-14.3 , 18.3)** |
| **Angola** | **53708 (42486 , 65864)** | **1261.1 (1014.7 , 1533.6)** | **159152 (128094 , 195516)** | **1306.9 (1068.7 , 1593.3)** | **3.6 (-18 , 32.5)** |
| **Central African Republic** | **19112 (15937 , 23342)** | **1485.2 (1244.4 , 1773)** | **40124 (31251 , 51382)** | **1628.1 (1287.3 , 2050.8)** | **9.6 (-12.4 , 35)** |
| **Congo** | **18759 (15646 , 22129)** | **1650.4 (1389.2 , 1943.7)** | **43655 (34902 , 54371)** | **1554.9 (1267.4 , 1899.6)** | **-5.8 (-24.1 , 16.3)** |
| **Democratic Republic of the Congo** | **195540 (161132 , 235804)** | **1185.2 (989.2 , 1406.1)** | **461010 (368885 , 571785)** | **1185.9 (959.6 , 1446)** | **0.1 (-18.7 , 20.1)** |
| **Equatorial Guinea** | **2845 (2232 , 3514)** | **1341.2 (1062 , 1644.1)** | **7945 (5936 , 10419)** | **1542.3 (1176.9 , 1986.5)** | **15 (-15.1 , 53.9)** |
| **Gabon** | **9878 (7766 , 12567)** | **1713.5 (1350.1 , 2169.4)** | **20673 (16597 , 25313)** | **1889.1 (1534.6 , 2301.9)** | **10.3 (-16.5 , 39)** |
